# Synthesis and Magnetic Properties of Bis-Halobenzene Decamethyldysprosocenium Cations

**DOI:** 10.1021/acs.inorgchem.3c04106

**Published:** 2024-02-21

**Authors:** Sophie C. Corner, Gemma K. Gransbury, Iñigo J. Vitorica-Yrezabal, George F. S. Whitehead, Nicholas F. Chilton, David P. Mills

**Affiliations:** Department of Chemistry, The University of Manchester, Oxford Road, Manchester M13 9PL, U.K.

## Abstract

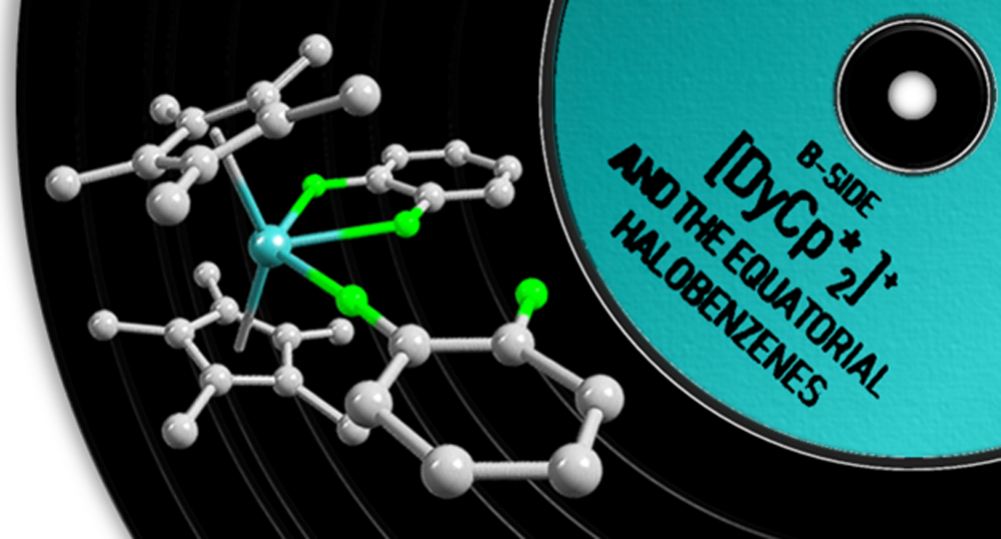

The decamethyldysprosocenium cation, [Dy(Cp*)_2_]^+^ (Cp* = {C_5_Me_5_}), was a target single-molecule magnet (SMM) prior to the isolation of larger dysprosocenium cations, which have recently shown magnetic memory effects up to 80 K. However, the relatively short Dy···Cp*_centroid_ distances of [Dy(Cp*)_2_]^+^, together with the reduced resonance of its vibrational modes with electronic states compared to larger dysprosocenium cations, could lead to more favorable SMM behavior. Here, we report the synthesis and magnetic properties of a series of solvated adducts containing bis-halobenzene decamethyldysprosocenium cations, namely [Dy(Cp*)_2_(PhX-κ-*X*)_2_][Al{OC(CF_3_)_3_}_4_] (X = F or Cl) and [Dy(Cp*)_2_(C_6_H_4_F_2_-κ^2^-*F*,*F*)(C_6_H_4_F_2_-κ-*F*)][Al{OC(CF_3_)_3_}_4_]. These complexes were prepared by the sequential reaction of [Dy(Cp*)_2_(μ-BH_4_)]_∞_ with allylmagnesium chloride and [NEt_3_H][Al{OC(CF_3_)_3_}_4_], followed by recrystallization from parent halobenzenes. The complexes were characterized by powder and single crystal X-ray diffraction, NMR and ATR-IR spectroscopy, elemental analysis, and SQUID magnetometry; experimental data were rationalized by a combination of density functional theory and *ab initio* calculations. We find that bis-halobenzene adducts of the [Dy(Cp*)_2_]^+^ cation exhibit highly bent Cp*···Dy···Cp* angles; these cations are also susceptible to decomposition by C–X (X = F, Cl, Br) activation and displacement of halobenzenes by O-donor ligands. The effective energy barrier to reversal of magnetization measured for [Dy(Cp*)_2_(PhF-κ-*F*)_2_][Al{OC(CF_3_)_3_}_4_] (930(6) cm^–1^) sets a new record for SMMs containing {Dy(Cp*)_2_} fragments, though all SMM parameters are lower than would be predicted for an isolated [Dy(Cp*)_2_]^+^ cation, as expected due to transverse ligand fields introduced by halobenzenes and the large deviation of the Cp*···Dy···Cp* angle from linearity promoting magnetic relaxation.

## Abstract 2

We report the synthesis and characterization of a family of single-molecule magnets, each containing decamethyldysprosocenium cations bound by two halobenzenes.

## Introduction

Single-molecule magnets (SMMs) exhibit retention of magnetization under certain conditions and therefore have potential applications in high density binary data storage.^1−3^ However, the viability of even the best-performing SMMs in such applications is currently limited as the desired magnetic properties have not yet been observed at temperatures that are suitable for practical usage.^4−15^ Some of the most promising high-temperature SMMs that have been studied so far are those involving dysprosocenium cations and related derivatives;^4−8^ the remarkable magnetic properties of this family of complexes have been attributed to the incorporation of substituted cyclopentadienyl (Cp^R^) ligands, which (i) provide the steric bulk required to achieve axial ligand fields, enhancing the magnetic anisotropy of the Dy^3+^ cation and hindering Orbach relaxation, and (ii) are rigid and thus impede molecular vibrations, suppressing Raman relaxation.^4,16,17^

Further stabilization of the ground *m*_*J*_ state of Dy^3+^ in dysprosocenium cations, and concurrent destabilization of the least magnetic *m*_*J*_ state, could be achieved by increasing the strength of the axial ligand field through the reduction of Dy···Cp^R^ distances by using smaller Cp^R^ ligands. The isolation of the decamethyldysprosocenium cation, [Dy(Cp*)_2_]^+^ (Cp* = {C_5_Me_5_}), has therefore long been a desirable target for the SMM community.^17,18^ The effective energy barrier to relaxation of magnetization (*U*_eff_) value of the hypothetical [Dy(Cp*)_2_]^+^ cation was calculated to be above 1000 cm^–1^ by Gao and co-workers in 2016.^18^ Recent work by Reta et al. concluded that although the linearity of the [Dy(Cp*)_2_]^+^ cation and therefore its *U*_eff_ value would be reduced compared to the [Dy(C_5_^i^Pr_5_)(Cp*)]^+^ cation,^8^ the vibrational modes may be shifted off-resonance with electronic states that could lead to comparatively slower relaxation rates.^17^ However, it has been shown that the relatively small steric bulk of the Cp* ligand (*cf*. C_5_^i^Pr_5_ and Cp^ttt^, C_5_H_2_^t^Bu_3_-1,2,4) is not sufficient to block equatorial ligand interactions and allow the isolation of the [Dy(Cp*)_2_]^+^ cation, even with weak donor ligands and weakly coordinating anions (WCAs).^18−40^ All SMMs containing {Dy(Cp*)_2_} fragments that have been reported to date have shown suppressed SMM properties compared to isolated dysprosocenium cations.^18,29^

Following our recent reports of the heteroleptic dysprosocenium complex [Dy(Cp^ttt^)(Cp*)][Al{OC(CF_3_)_3_}_4_]^15^ and its monohalobenzene-solvated adducts,^41^ we resolved to apply these methods to {Dy(Cp*)_2_} analogues. Here, we report the synthesis of the putative WCA-bound complexes “[{Ln(Cp*)_2_}{Al[OC(CF_3_)_3_]_4_}]” (Ln = Y, Dy), together with a family of bis-halobenzene-bound {Dy(Cp*)_2_}^+^ cations with the same WCA derived from this starting material. We find that the reduced steric bulk of the bis-Cp* ligand environment enables {Dy(Cp*)_2_} fragments with significantly bent Cp*···Dy···Cp* angles, permitting a greater number of transverse ligand interactions and increasing the propensity of the resultant cations to promote C–X activation and form decomposition products. The bis-halobenzene adducts of {Dy(Cp*)_2_}^+^ are characterized by single crystal (SCXRD) and powder X-ray Diffraction (PXRD), elemental analysis, ATR-IR spectroscopy, elemental analysis, and SQUID magnetometry. The magnetic data are rationalized by complete active space self-consistent field spin–orbit (CASSCF-SO) calculations. We find that coordination of two haloarenes to {Dy(Cp*)_2_} moieties promotes magnetic relaxation via structural distortions and an increased transverse field, but we observe a *U*_eff_ value of 930(6) cm^–1^ for [Dy(Cp*)_2_(PhF-κ-*F*)_2_][Al{OC(CF_3_)_3_}_4_] that sets a new benchmark for {Dy(Cp*)_2_}-containing SMMs.

## Results

### Synthesis

**Scheme 1:**
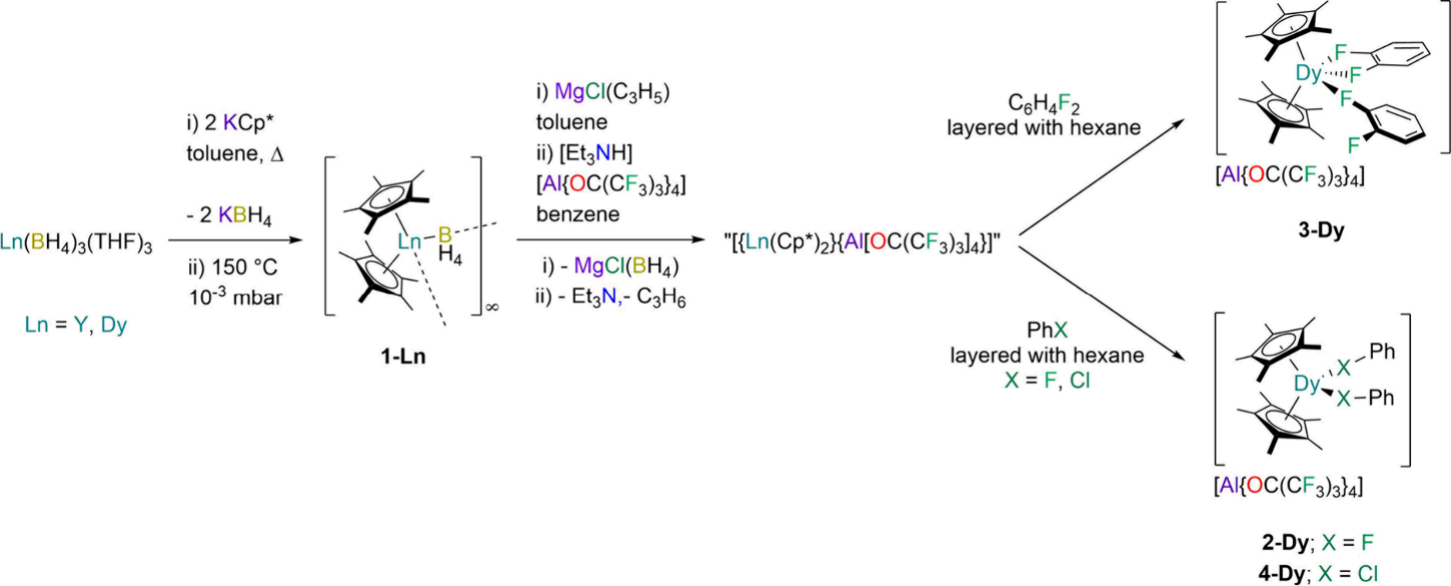
Synthesis of **1-Ln**, “[{Ln(Cp*)_2_}{Al[OC(CF_3_)_3_]_4_}]”, **2-Dy**, **3-Dy**, and **4-Dy**

The heteroleptic complexes [Ln(Cp*)_2_(μ-BH_4_)]_∞_ (**1-Ln**; Ln = Y, Dy) were obtained in moderate yields (*ca*. 55%) by the separate salt metathesis reactions of [Ln(BH_4_)_3_(THF)_3_] with two equivalents of KCp* in toluene at reflux, followed by desolvation of crude “[Ln(Cp*)_2_(BH_4_)(THF)_*x*_]” by heating under vacuum (150 °C, 10^–3^ mbar) for 6 h and recrystallization from toluene (Scheme 1). Crystals of **1-Ln** suitable for SCXRD analysis were obtained from saturated methylcyclohexane solutions. The separate salt metathesis reactions of **1-Ln** with MgCl(C_3_H_5_) in toluene were assumed to form the allyl complexes “[Ln(Cp*)_2_(C_3_H_5_)]”, by adapting previously reported protocols;^19,42^ these crude materials were treated with [NEt_3_H][Al{OC(CF_3_)_3_}_4_] (0.95 equiv) in benzene, and after workup, yellow powders of putative “[{Ln(Cp*)_2_}{Al[OC(CF_3_)_3_]_4_}]” were obtained in moderate yields (52–63% based on **1-Ln**; Scheme 1). As with the previously reported analog “[{Ln(Cp^ttt^)(Cp*)}{Al[OC(CF_3_)_3_]_4_}]”,^15^ elemental analysis, combined with solution state studies, revealed that the triethylamine side-product persisted in samples of “[{Ln(Cp*)_2_}{Al[OC(CF_3_)_3_]_4_}]” despite multiple washes with benzene and *n*-hexane (see the ESI for full synthetic details). Portions of “[{Dy(Cp*)_2_}{Al[OC(CF_3_)_3_]_4_}]” were separately dissolved in a range of halobenzenes to give the solvated adducts [Dy(Cp*)_2_(PhF-κ-*F*)_2_][Al{OC(CF_3_)_3_}_4_] (**2-Dy**), [Dy(Cp*)_2_(C_6_H_4_F_2_-κ^2^-*F*,*F*)(C_6_H_4_F_2_-κ-*F*)][Al{OC(CF_3_)_3_}_4_] (**3-Dy**), and [Dy(Cp*)_2_(PhCl-κ-*Cl*)_2_][Al{OC(CF_3_)_3_}_4_] (**4-Dy**) as yellow crystals in almost quantitative yields, following workup and recrystallization. Crystals of [Dy(Cp*)_2_(PhBr-κ-*Br*)_2_][Al{OC(CF_3_)_3_}_4_] (**5-Dy**) were observed in samples of “[{Dy(Cp*)_2_}{Al[OC(CF_3_)_3_]_4_}]” recrystallized from PhBr, but these crops were contaminated with byproducts due to facile decomposition (see below).

The attempted recrystallizations of separate samples of “[{Dy(Cp*)_2_}{Al[OC(CF_3_)_3_]_4_}]” in benzene at 6 °C and either toluene or α,α,α-trifluorotoluene at −35 °C, all layered with *n*-hexane, consistently resulted in the formation of crystals of the dinuclear complex [{Dy(Cp*)_2_}_2_(μ-F)][Al{OC(CF_3_)_3_}_4_] (**6-Dy**). Presumably this occurs by an sp^3^ C–F bond activation promoted by the highly Lewis acidic Dy^3+^ center, involving either the [Al{OC(CF_3_)_3_}_4_]^−^ anion or the α,α,α-trifluorotoluene solvent. Further evidence of residual triethylamine was presented by the isolation of crystals of [NEt_3_(CF_2_C_6_H_5_)][Al{OC(CF_3_)_3_}_4_] (**7**) from a recrystallization attempt using α,α,α-trifluorotoluene. Additionally, C–Br bond activation was observed when “[{Dy(Cp*)_2_}{Al[OC(CF_3_)_3_]_4_}]” was dissolved in bromobenzene; crystals of [{Dy(Cp*)_2_}_2_(μ-Br)][Al{OC(CF_3_)_3_}_4_] (**8-Dy**) and [{Dy(Cp*)_2_(PhBr-κ-Br)}_2_(μ-Br)][Al{OC(CF_3_)_3_}_4_] (**9-Dy**) were consistently identified in batches of **5-Dy**, and these impurities precluded the isolation of pure **5-Dy** for further characterization data to be obtained.

During various attempts to isolate **2-Dy**, **3-Dy**, **4-Dy**, and **5-Dy**, the unintentional introduction of trace amounts of coordinating solvents led to the formation of oxygen-bound adducts that could be identified by SCXRD, including [Dy(Cp*)_2_(DME)][Al{OC(CF_3_)_3_}_4_] (**10-Dy**) and [Dy(Cp*)_2_(PhBr-κ-*Br*)(THF)][Al{OC(CF_3_)_3_}_4_] (**11-Dy**). A small portion of “[{Dy(Cp*)_2_}{Al[OC(CF_3_)_3_]_4_}]” dissolved in THF, layered with *n*-hexane, and stored at −30 °C gave several crystals of [Dy(Cp*)_2_(THF)_2_][Al{OC(CF_3_)_3_}_4_] (**12-Dy**); the two bound THF molecules are akin to the closely related analog [Dy(Cp^ttt^)(Cp*)(THF)_2_][Al{OC(CF_3_)_3_}_4_]^41^ and the comparable group 3 complex [Sc(Cp*)_2_(THF)_2_][BPh_4_].^43^

Several small-scale reactions were performed with **1-Y** to allow scoping studies with a diamagnetic material before transferring the methodology to **1-Dy**. The reaction of **1-Y** with [CPh_3_][Al{OC(CF_3_)_3_}_4_] in fluorobenzene and subsequent layering with *n*-hexane gave several crystals of [Y(Cp*)_2_(PhF-κ-*F*)_2_][Al{OC(CF_3_)_3_}_4_] (**2-Y**). However, the attempted direct synthesis of **2-Dy** by analogous procedures gave crystals of [{Dy(Cp*)_2_(PhF-κ-*F*)}_2_(μ-BH_4_)][Al{OC(CF_3_)_3_}_4_] (**13-Dy**) together with those of **2-Dy**; thus, this methodology was not transferred to other halobenzenes. The use of a different WCA was also investigated by reacting **1-Y** with [(Et_3_Si)_2_(μ-H)][B(C_6_F_5_)_4_] in fluorobenzene. Several crystals of [Y(Cp*)_2_{B(C_6_F_5_)_4_-κ-*F*}(PhF-κ-*F*)] (**14-Y**) formed after layering the reaction mixture with *n*-hexane; SCXRD revealed that the WCA coordinates to the Y center in the solid state, and thus, no further syntheses were attempted with [(Et_3_Si)_2_(μ-H)][B(C_6_F_5_)_4_].

### NMR Spectroscopy

NMR spectra were collected on **1-Ln**, “[{Ln(Cp*)_2_}{Al[OC(CF_3_)_3_]_4_}]”, **2-Dy**, **3-Dy**, and **4-Dy** (see the ESI for all NMR spectra). The ^1^H NMR spectrum of **1-Y** contains a singlet at 1.93 ppm with a relative integral of 30H corresponding to the Cp* ring protons. A broad quartet with an integral of 4H centered at 0.09 ppm was assigned to the BH_4_^–^ group; the ^1^*J*_BH_ coupling constant of 85.9 Hz is as expected for a diamagnetic complex,^44^ and no ^89^Y–^1^H coupling could be resolved. The ^13^C{^1^H} NMR spectrum of **1-Y** confirms the presence of only two carbon environments for the Cp* ligands, with peaks at 12.1 and 118.5 ppm; there are no signals that correspond to residual THF in either the ^1^H or ^13^C{^1^H} NMR spectrum. The ^11^B NMR spectrum of **1-Y** exhibits a pentet at −24.12 ppm (^1^*J*_BH_ = 85.9 Hz), indicating a single boron environment in solution at room temperature. The paramagnetism of **1-Dy** precluded the assignment of its NMR spectra.

For “[{Y(Cp*)_2_}{Al[OC(CF_3_)_3_]_4_}]”, multinuclear NMR experiments were performed after dissolving the sample in fluorobenzene, forming **2-Y***in situ*. Analysis of the ^1^H and ^13^C{^1^H} NMR spectra reveals the presence of approximately 0.75 equiv of triethylamine in addition to the expected Cp* signals. In the ^19^F NMR spectrum of this sample, the signals resulting from the [Al{OC(CF_3_)_3_}_4_]^−^ anion and fluorobenzene are in the expected regions (−75.3 and −113.9 ppm, respectively),^45,46^ representative of transient coordination in solution. For complexes **2-Dy**, **3-Dy**, and **4-Dy**, ^1^H and ^19^F NMR spectra were collected in the respective halobenzene; “[{Dy(Cp*)_2_}{Al[OC(CF_3_)_3_]_4_}]” was dissolved in fluorobenzene and presented comparable results to **2-Dy**; in the ^19^F NMR spectrum, the signals of the [Al{OC(CF_3_)_3_}_4_]^−^ anion are paramagnetically broadened and shifted when compared to **2-Y** (**2-Dy**: −78.9 ppm, υ_1/2_ ∼ 140 Hz; **3-Dy**: −72.8 ppm, υ_1/2_ ∼ 120 Hz; **4-Dy**: −76.6 ppm, υ_1/2_ ∼ 220 Hz). Similar paramagnetic effects are observed for the fluorobenzene and *ortho*-difluorobenzene solvent signals in **2-Dy** (−131.1 ppm, υ_1/2_ ∼ 2470 Hz) and **3-Dy** (−153.8 ppm, υ_1/2_ ∼ 1410 Hz). Both factors are indicative of fluxional coordination of the halobenzene and WCA to the Dy^3+^ center in solution.

### Bulk Solid-State Characterization

Owing to the difficulties of determining bulk purities or structural features for paramagnetic complexes in solution, solid-state characterization techniques were employed. Elemental analysis was performed on complexes **1-Ln**, “[{Ln(Cp*)_2_}{Al[OC(CF_3_)_3_]_4_}]”, **2-Dy**, **3-Dy**, and **4-Dy** (see the ESI for full details of supporting characterization data), confirming trace amounts of nitrogen in samples of “[{Ln(Cp*)_2_}{Al[OC(CF_3_)_3_]_4_}]”. The halobenzene-containing complexes **2-Dy**, **3-Dy**, and **4-Dy** did not contain detectable nitrogen; however, consistently low carbon values were observed, which we attribute to carbide formation as this is a common issue for fluorine-rich compounds.^47^ PXRD was performed on microcrystalline samples of **2-Dy**, **3-Dy**, and **4-Dy** to determine phase purities. The observed data, processed with Pawley refinement,^48^ are in excellent agreement with those calculated from the SCXRD data (Figures S18–S23).

For **1-Ln**, the ATR-IR spectra feature aliphatic C–H stretches within the expected wavenumber range (3000–2800 cm^–1^). Additionally, one broad band is present in the region corresponding to a bridging bidentate BH_4_^–^ group (*ca*. 2250 cm^–1^); no peaks for terminal B–H stretches are observed.^49^ DFT calculations were performed on the diamagnetic Y analogs of the cations of **2-Dy**, **3-Dy**, and **4-Dy** ([Y(Cp*)_2_(PhF-κ-*F*)_2_]^+^, **2′-Y**; [Y(Cp*)_2_(C_6_H_4_F_2_-κ^2^-*F*,*F*)(C_6_H_4_F_2_-κ-*F*)]^+^, **3′-Y**; [Y(Cp*)_2_(PhCl-κ-*Cl*)_2_]^+^, **4′-Y**) to predict their vibrational modes (calculations at the PBE0-D4/def2-TZVP level; see the ESI for full details). The computational results are in good agreement with the measured ATR-IR spectra, allowing for the assignment of the pertinent peaks unique to each compound; significantly, C–F stretches are observed at 781 and 1124 cm^–1^ for **2-Dy** and at 752 and 1089 cm^–1^ for **3-Dy**, and the C–Cl stretches of **4-Dy** are at 686 and 1060 cm^–1^ (see the ESI for recorded and simulated spectra). The remainder of the discernible peaks in the ATR-IR spectra of **2-Dy**, **3-Dy**, and **4-Dy** are in accord with those observed for “[{Dy(Cp*)_2_}{Al[OC(CF_3_)_3_]_4_}]”, indicating that their bulk structural features are similar.

### Single Crystal X-ray Diffraction

**Figure 1:**
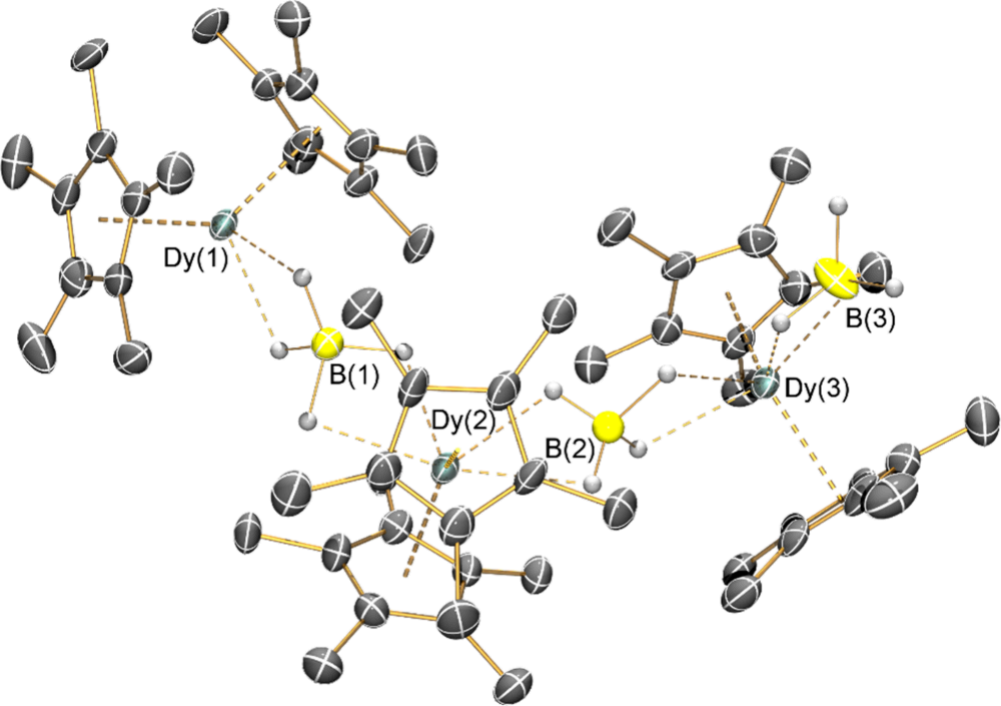
SCXRD structure of a portion of polymeric **1-Dy** with select atom labeling (Dy: cyan, C: gray, B: yellow, H: white). Displacement ellipsoids are set at a 50% probability level; lattice solvent and hydrogen atoms are omitted for clarity, with the exception of those belonging to the BH_4_^–^ groups. Selected mean distances and angles: Dy···Cp*_centroid_ 2.379(2) Å, Dy···B 2.954(3) Å, Cp*_centroid_···Dy···Cp*_centroid_ 133.44(8)°, B···Dy···B 104.3(2)°.

The solid-state structures of **1-Ln**, **2-Ln**, **3-Dy**, **4-Dy**, **5-Dy**, **6-Dy**, **7**, **8-Dy**, **9-Dy**, **10-Dy**, **11-Dy**, **12-Dy**, **13-Dy**, and **14-Y** were determined by SCXRD (see the ESI for full crystallographic details and depictions of all complexes). Half a molecule of chlorobenzene was found in the intermolecular spaces in the solid state structure of **4-Dy**, while 0.5 eq. and 0.33 eq. of molecules of methylcyclohexane were respectively found in the crystal lattices of **1-Y** and **1-Dy**. Complexes **1-Ln** are structurally analogous, and thus, only **1-Dy** is shown here (Figure 1); both complexes form single chain helical 1D coordination polymers, comprising three independent {Ln(Cp*)_2_} units with bridging BH_4_^–^ groups bound in κ^2^-coordination modes to the two Ln centers, consistent with the IR spectral data (see above). The mean Dy···B distance in **1-Dy** is 2.954(3) Å, the average B···Dy···B angle is 104.3(2)°, the mean Dy···Cp* distances are 2.379(2) Å, and the mean Cp*···Dy···Cp* angle is 133.44(8)°.

**Figure 2:**
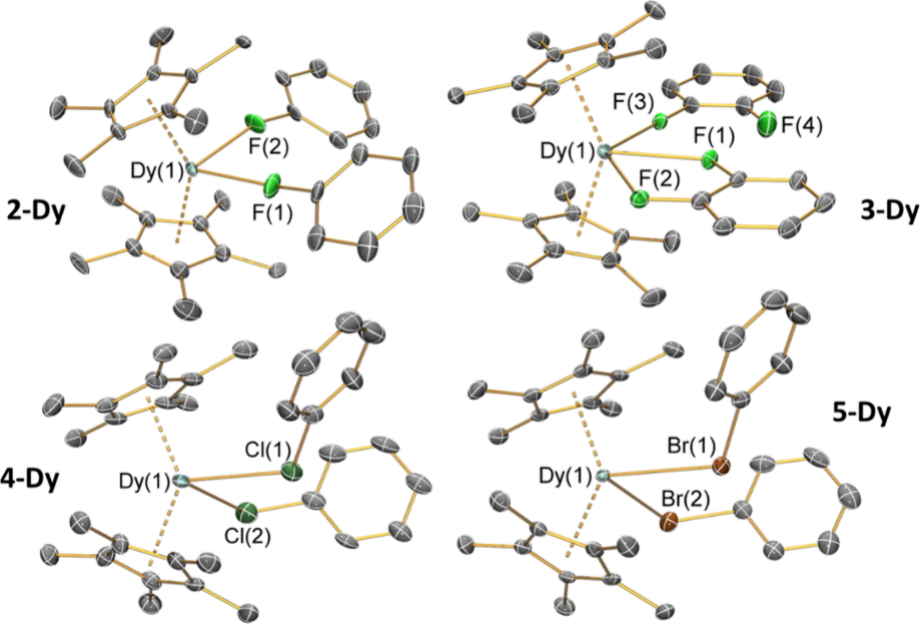
SCXRD structures of **2-Dy**, **3-Dy**, **4-Dy**, and **5-Dy** with selected atom labeling (Dy: cyan, C: gray, F: green, Cl: dark green, Br: brown). Displacement ellipsoids are set at 30% (**2-Dy** and **3-Dy**) and 50% (**4-Dy** and **5-Dy**) probability levels. Hydrogen atoms, the lattice solvent molecule, and [Al{OC(CF_3_)_3_}_4_]^−^ anions have been omitted. Selected bond lengths and angles are compiled in Table 1.

The solid state structures of **2-Dy**, **4-Dy**, and **5-Dy** (Figure 2; **2-Y** crystallized in a different space group to **2-Dy** but the bulk structural features of these complexes are comparable) reveal that in each case two halobenzenes are κ^1^-coordinated to the {Dy(Cp*)_2_} fragments via lone pairs of the halogen atoms, in a similar manner to that previously seen for [Sc(Cp*)_2_(PhF-κ-*F*)_2_][BPh_4_].^43^ In contrast, for **3-Dy**, one *ortho-*difluorobenzene molecule binds in a κ^2^-coordination mode, with the other κ^1^-coordinated through a single fluorine (Figure 2). For **3-Dy**, there are four ion pairs within the P1 unit cell; thus, the atomic coordinates of the two least disordered ion pairs were used for comparative purposes and for subsequent calculations. Increasing the size of the halogen in the coordinated solvent leads to increased mean Dy–X distances [**2-Dy**: 2.358(12) Å; **4-Dy**: 2.920(3) Å; **5-Dy**: 3.064(3) Å] as expected;^41^ subtracting the covalent radii of the halogens (F: 0.64 Å; Cl: 0.99 Å; Br: 1.14 Å)^50^ from these distances gives a value of *ca*. 1.93 Å for both X = Cl and Br. The Dy–F distances observed for **3-Dy** [Dy–F(1): 2.497(3) Å; Dy–F(2): 2.609(4) Å; Dy–F(3): 2.492(5) Å] are longer than those seen in **2-Dy**, due to increased steric buttressing in the former complex. The orientations of the halobenzenes vary according to the halogen, with Dy–X–C_ipso_ angles of 166.1(10)° and 162.3(9)° for **2-Dy**, 115.0(2)° and 117.56(7)° for **4-Dy**, and 111.77(7)° and 117.56(7)° for **5-Dy**; the corresponding angles for **3-Dy** [175.1(3)° and 175.9(2)°] are closer to linearity. The C–X bond lengths observed in the halobenzene adducts [**2-Dy**: 1.42(3) Å; **4-Dy**: 1.760(6) Å; **5-Dy**: 1.924(4) Å] are longer than those seen for the unbound solvents [PhF: 1.36(2) Å; PhCl: 1.737(5) Å; PhBr 1.899(12) Å].^51^ For **3-Dy**, the C–F bond length of the uncoordinated F atom [1.347(10) Å] is statistically equivalent to the coordinated counterparts [mean 1.375(5) Å] (see Table 1).

**Table 1:** Selected Bond Distances and Angles of **2-Dy**, **3-Dy**, **4-Dy**, and **5-Dy**

	**2-Dy**^a^	**3-Dy**^a^	**4-Dy**	**5-Dy**
Dy···Cp*_centroid_	2.2763(8) Å	2.321(2) Å	2.317(4) Å	2.3241(11) Å
Dy···Cp*_centroid_	2.2957(7) Å	2.339(2) Å	2.324(4) Å	2.3267(11) Å
Cp*_centroid_···Dy···Cp*_centroid_	142.49(4)°	143.34(8)°	138.88(13)°	139.06(4)°
Dy–X(1)	2.346(9) Å	2.497(3) Å	2.935(2) Å	3.0761(4) Å
Dy–X(2)	2.369(8) Å	2.609(4) Å	2.904(2) Å	3.0512(6) Å
Dy–F(3)	-	2.492(5) Å	-	-
X(1)–C	1.42(2) Å	1.373(6) Å	1.788(8) Å	1.923(3) Å
X(2)–C	1.42(2) Å	1.373(8) Å	1.746(13) Å	1.925(2) Å
F(3)–C	-	1.378(8) Å	-	-
F(4)–C	-	1.347(10) Å	-	-
X(1)–Dy–X(2)	75.7(4)°	-	78.45(6)°	78.73(2)°
F(1)–Dy–F(3)	-	68.0(2)°	-	-
Dy–X_1_–C_ipso_	166.1(10)°	-	115.0(2)°	111.77(7)°
Dy–X_2_–C_ipso_	162.3(9)°	-	121.3(3)°	117.56(7)°
Dy–F_1/2_–C_ipso_	-	175.1(3)°	-	-
Dy–F_3_–C_ipso_	-	175.9(2)°	-	-

a Metrical parameters are for the least disordered molecules in the unit cell.

The orientation, denticity, and size of the coordinated halobenzenes also influences the arrangement of the Cp* ligands; the ligands in **2-Dy**, **4-Dy**, and **5-Dy** adopt a staggered conformation, whereas for **3-Dy**, the rings are near-eclipsed (Figure S26). Complex **2-Dy** exhibits the shortest Dy···Cp*_centroid_ distance [2.286(2) Å], while the analogous distances in **3–5-Dy** are all statistically equivalent [**3-Dy**: 2.330(3) Å; **4-Dy**: 2.321(6) Å; **5-Dy**: 2.325(2) Å]. From consideration of the Cp*_centroid_···Dy···Cp*_centroid_ angles, **3-Dy** is the most obtuse [143.34(8)°], followed by **2-Dy** [142.49(4)°], then statistically equivalent **4-Dy** [138.88(13)°] and **5-Dy** [139.06(4)°].

### Magnetism

**Figure 3:**
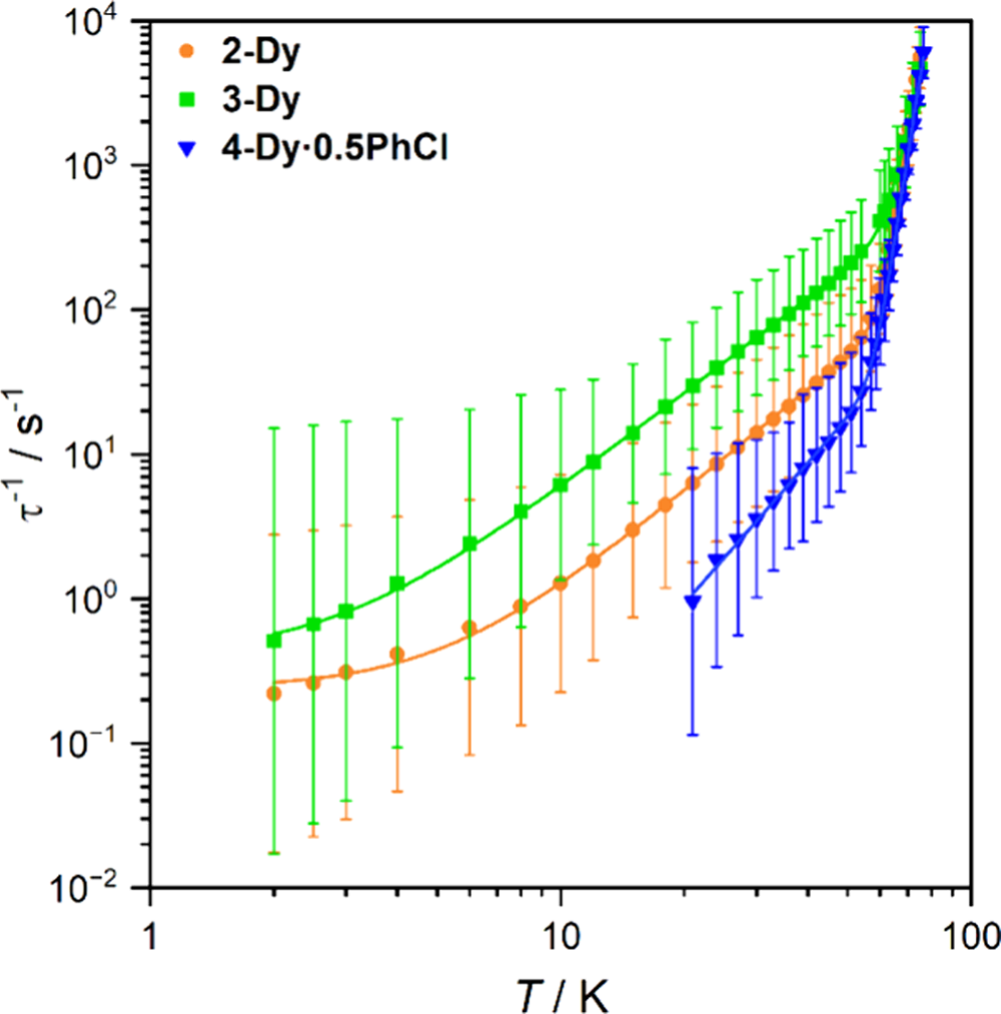
Relaxation profiles for **2-Dy, 3-Dy**, and **4-Dy**. Error bars represent one ESD in the distribution of rates.

The magnetic properties of “[{Dy(Cp*)_2_}{Al[OC(CF_3_)_3_]_4_}]”, **2-Dy**, **3-Dy**, and **4-Dy** were probed by SQUID magnetometry (see Figure 3 for relaxation profiles, Table 2 for selected SMM parameters, and the ESI for other magnetic data); due to the amorphous nature and unknown purity of “[{Dy(Cp*)_2_}{Al[OC(CF_3_)_3_]_4_}]”, the resultant data are not discussed here (see the ESI for details). The magnetic susceptibility products (χ*T*) at 300 K, measured in a 0.1 T dc field, are 13.3, 14.0, and 14.1 cm^3^ K mol^–1^ for **2-Dy**, **3-Dy**, and **4-Dy**, respectively; some deviation from the free-ion Dy(III) value (^6^H_15/2_, χ*T* = 14.2 cm^3^ K mol^–1^)^52^ is expected due to splitting of the *m*_*J*_ states by the crystal field. These values are comparable to those calculated using *ab initio* methods (see below and the ESI for full details). The susceptibilities decrease monotonically with temperature owing to the thermal depopulation of excited *m*_*J*_ states but rapidly decrease below 7 K for **3-Dy** and 22 K for **2-Dy** and **4-Dy**.

**Table 2:** Selected SMM Parameters for **2–4-Dy**

	**2-Dy**	**3-Dy**	**4-Dy**
*U*_eff_/cm^–1^	930(6)	776(11)	921(6)
*A* (τ_0_ = 10^*A*^/s)	–11.48(5)	–10.07(10)	–11.27(6)
*R* (*C* = 10^*R*^/s^–1^ K^–*n*^)	–2.34(3)	–1.44(3)	–4.24(9)
*n*	2.37(2)	2.20(2)	3.23(6)
*Q* (τ_QTM_ = 10^*Q*^/s)	0.62(2)	0.39(4)	-
*T*_H_/K	8	4	10

Magnetization values at 2 K and 5 T were measured for **2-Dy** (4.99 N_A_μ_B_), **3-Dy** (5.09 N_A_μ_B_), and **4-Dy** (5.20 N_A_μ_B_) (Figures S54, S56, and S58), which are all close to the expected value of 5.00 N_A_μ_B_ for a pure *m*_*J*_ = ±15/2 ground state.^53^ Magnetization vs. applied dc field measurements over a range of temperatures revealed butterfly shaped hysteresis loops that are open at zero-field and close with increasing temperatures (Figures S69–S74); using a sweep rate of 22 Oe s^–1^, the maximum temperatures at which the loops are open (*T*_H_) are 8 K, 4 K, and 10 K for **2-Dy**, **3-Dy**, and **4-Dy**, respectively.

Ac susceptibilty measurements (0.1–1000 Hz) were performed to investigate the dynamic magnetic behavior of **2-Dy**, **3-Dy**, and **4-Dy** at zero dc field. Peaks observed in the out-of-phase susceptibilities (χ_M_″) are representative of slow magnetic relaxation; the temperature ranges of these peaks are 6–75 K for **2-Dy**, 2–75 K for **3-Dy**, and 21–76.5 K for **4-Dy**. The collected ac data were fit to the generalized Debye model in CC-FIT2^54,55^ to determine relaxation rates and their distributions. Under low temperature regimes, we also observe minor components of fast relaxation processes; these data cannot be modeled reliably and have been excluded when fitting the major relaxation process (see the ESI for details). At high temperatures (*ca*. > 50 K), Orbach relaxation processes dominate, with extracted *U*_eff_ values of 930(6) cm^–1^ for **2-Dy**, 776(11) cm^–1^ for **3-Dy**, and 921(6) cm^–1^ for **4-Dy**; the respective τ_0_^–1^ prefactors are 10^11.48(5)^, 10^10.07(10)^, and 10^11.27(6)^ s^–1^ (Table 2, Figure 3 and Figures S94–S96). Raman relaxation, with a *CT*^*n*^ power law dependency, predominates for the remaining measurable behavior [**2-Dy**: *C* = 10^–2.34(3)^ s^–1^ K^–*n*^, *n* = 2.37(2); **3-Dy**: *C* = 10^–1.44(3)^ s^–1^ K^–*n*^, *n* = 2.20(2); **4-Dy**: *C* = 10^–4.24(9)^ s^–1^ K^–*n*^, *n* = 3.23(6)]; quantum tunnelling of the magnetization (QTM) components can be fit for **2-Dy** and **3-Dy** [10^0.62(2)^ s and 10^0.39(4)^ s, respectively] but are too slow to observe for **4-Dy**.

### Ab Initio Calculations

The electronic structures of [Dy(Cp*)_2_(PhF-κ-*F*)_2_]^+^ (**2′-Dy**), [Dy(Cp*)_2_(C_6_H_4_F_2_-κ^2^-*F*,*F*)(C_6_H_4_F_2_-κ-*F*)]^+^ (**3′-Dy**), [Dy(Cp*)_2_(PhCl-κ-*Cl*)_2_]^+^ (**4′-Dy**), and [Dy(Cp*)_2_(PhBr-κ-*Br*)_2_]^+^ (**5′-Dy)** were investigated by CASSCF-SO calculations using OpenMOLCAS^56^ and coordinates from single crystal XRD structures (see the ESI for full details). While the magnetic properties for **5-Dy** could not be measured, calculations are provided for completeness. Calculations were also performed on the theoretical complex [Dy(Cp*)_2_][Al{OC(CF_3_)_3_}_4_] for comparative purposes; the coordinates were obtained from DFT geometry optimization of the diamagnetic Y analogue (calculations at the PBE0-D4/def2-TZVP level; see the ESI for full details), which was initially constructed using the solid state atomic coordinates of **4-Dy**. The shortest Dy···F distance of the optimized structure is relatively long, at 4.012 Å, which is reflected in the high calculated *U*_eff_ value of 1284 cm^–1^.

For **2′-Dy**, **3′-Dy**, **4′-Dy**, and **5′-Dy**, the principal ground state magnetic axis (*g*_*z*_) traverses the two Cp* rings. The ground state Kramers doublets are highly axial with Ising-like *g*-values for **2′-Dy**, **3′-Dy**, **4′-Dy**, and **5′-Dy** of *g*_*x*_ ∼ *g*_*y*_ ∼ 0, *g*_*z*_ ∼ 19.8, suggesting dominant *m*_*J*_ = ±15/2 contributions (Tables S20–S23). Axial Cp*-Dy-Cp* geometries and weak equatorial solvent coordination are sufficient to give highly pure (98–100%) *m*_*J*_ states across the first three excited states for **2′-Dy**, **4′-Dy**, and **5′-Dy**. At the fourth excited state (*m*_*J*_ = ±7/2), the purity is lost to give calculated *U*_calc_ values of 766 cm^–1^, 825, and 821 cm^–1^. A better agreement with experiment is provided by Orbach relaxation via the sixth or fifth excited state for **2-Dy** and **4-Dy**, respectively [**2′-Dy**: 881 cm^–1^, **4′-Dy**: 889 cm^–1^]. For **3′-Dy**, high *m*_*J*_ purity is retained up to the fourth excited state (96% *m*_*J*_ = ±7/2); however, the transverse *g*-factors are significant here (*g*_*x*_ = 2.4, *g*_*y*_ = 2.9, and *g*_*z*_ = 8.7), suggesting Orbach relaxation is still likely to not proceed higher than this doublet, and hence *U*_calc_ = 837 cm^–1^, in reasonable agreement with experiment (*U*_eff_ = 776(11) cm^–1^). As observed for halobenzene adducts of {Dy(Cp^ttt^)(Cp*)}^+^_,_^41^ the effect of *o*-C_6_H_4_F_2_ coordination is well-reproduced by CASSCF-SO, while calculations overestimate the disruption of the axiality on monohalobenzene coordination, resulting in underpredicting experimental *U*_eff_ values.

As Gao and co-workers previously reported that the most favorable magnetic properties for the [Dy(Cp*)_2_(X)(THF)] (X = F, Cl, Br, I) family was for X = I,^18^ DFT optimization and subsequent CASSCF-SO calculations were performed on the theoretical [Dy(Cp*)_2_(PhI-κ-*I*)_2_]^+^ cation (**15′-Dy**), starting from the molecular structure of **4′-Dy** (see the ESI for details). The optimized structure exhibits mean Dy···Cp*_centroid_ distances of 2.321 Å, a Cp*_centroid_···Dy···Cp*_centroid_ angle of 141.15°, and a mean Dy–I distance of 3.194 Å (covalent radii of I = 1.33 Å);^50^ however, note that gas phase DFT calculations typically give reduced bond lengths.^17^ Calculations on **15′-Dy** predict a reduced *U*_eff_ of 778 cm^–1^ compared to **5′-Dy**. Owing to the significant C–Br bond cleavage that occurs when synthesizing **5-Dy**, and the increased susceptibility of C–I bonds to activation,^51^ it is unlikely that [Dy(Cp*)_2_(PhI-κ-*I*)_2_][Al{OC(CF_3_)_3_}_4_] can be isolated by the methods used herein.

## Discussion

For axial Dy^3+^ complexes, the exclusion of equatorially coordinating ligands is inherently challenging due to the relatively large size of the highly Lewis acidic Dy^3+^ ions, the lack of directionality in Ln bonding regimes, and the tendency to maximize contacts with hard ligand donor atoms.^57^ The difficulties encountered in the isolation of axial dysprosocenium cations arise from these factors. Despite the careful exclusion of unwanted donor solvents, the {Dy(Cp*)_2_}^+^ cation demonstrates a tendency for C–X bond activation, which precluded the crystallization of solvent-free [{Dy(Cp*)_2_}{Al[OC(CF_3_)_3_]_4_}]. Similar sp^3^ C–F bond cleavage has previously been observed for this WCA^15,58^ and for the related group 4 M^3+^ complex [Ti(Cp*)_2_][BPh_4_], which forms [Ti(Cp*)_2_(F)] when dissolved in α,α,α-trifluorotoluene or when [B{C_6_H_3_(CF_3_)_2_-3,5}_4_]^−^ is introduced as a WCA.^59^ While rare earth-induced C–Br bond activation reactions are scarce, C–X bond dissociation energies decrease with increasing halogen size,^60,61^ accounting for the increased propensity of its occurrence for **5-Dy**. Evidence of C–X activation is also present in the solid state structures of **2-Dy**, **4-Dy**, and **5-Dy** through the elongation of C–X bond distances when compared to the free solvent molecule.^51^ This effect is diminished for **3-Dy**, with the C–F bond of the uncoordinated F atom being statistically comparable to the coordinated counterparts; this is likely a result of the bidentate nature of the halobenzene lowering the extent of activation for individual C–F bonds.

The reduced steric requirements of the ligand field of {Dy(Cp*)_2_}^+^ compared to {Dy(Cp^ttt^)(Cp*)}^+^ led to two halobenzenes coordinating equatorially in **2-Dy**, **4-Dy**, and **5-Dy**, validating our previous predictions made with the *AtomAccess* program.^15,41^ Crystals of [Y(Cp^ttt^)(Cp*)(PhF-κ-*F*)_2_][Al{OC(CF_3_)_3_}_4_] were previously shown to form in fluorobenzene solutions of “[Y(Cp^ttt^)(Cp*){Al[OC(CF_3_)_3_]_4_}]” at room temperature;^41^ this indicates that crystallization conditions can influence the coordination number. The mixed denticity of the halobenzene ligands in **3-Dy** is attributed to coordinative saturation. In common with the [Ln(Cp^ttt^)(Cp*)(PhX-κ-*X*)][Al{OC(CF_3_)_3_}_4_] series,^41^ for **2-Dy**, **4-Dy**, and **5-Dy**, an increased bending of the Dy–X–C_ipso_ angle is observed with halogen size; this can be attributed to the anisotropic electrostatic potential distribution around the halide atoms.^41,51^ The variance in angle is greatest between PhF and PhCl, which is likely due to the much longer Dy–X distances for the heavier halogen. The increased steric hindrance and less Lewis basic fluorine atoms of *o*-C_6_H_4_F_2_ in **3-Dy** compared to the donor atoms of PhF in **2-Dy** leads to the observed increase in Dy–F distances, while the steric influence of the ligand field leads to near-linear Dy–F–C_ipso_ angles in the former complex.

In contrast with {Dy(Cp^ttt^)(Cp*)}-containing complexes, variations in the coordinated halobenzenes in **2-Dy**, **3-Dy**, **4-Dy**, and **5-Dy** induce distortion of their {Dy(Cp*)_2_} cores; this is likely a result of the increased steric buttressing caused by two coordinated solvent molecules in the latter series rather than one in the former. Magnetostructural correlations can usually be drawn from the comparative axialities of crystal fields, but this analysis is not straightforward for **2-Dy**, **3-Dy**, **4-Dy**, and **5-Dy**. The shortest Dy···Cp*_centroid_ distances are observed for **2-Dy**, due to a combination of the solvent coordinating more linearly than in **4-Dy** and **5-Dy** and PhF being less bulky than *o*-C_6_H_4_F_2_ in **3-Dy**; the Cp*_centroid_···Dy···Cp*_centroid_ angle of **2-Dy** is also more obtuse than **4-Dy** and **5-Dy**. Both features should more favorably stabilize an oblate ground state, but these factors are likely offset by the relative proximity of the bound PhF molecules and the expected increased strength of Dy–F vs. Dy–Cl and Dy–Br interactions. The slightly faster Orbach rates for PhF coordination compared to PhCl (Figure S104), as observed for {Dy(Cp^ttt^)(Cp*)}-halobenzene adducts, indicate that the strength of the Dy–X interaction dominates, although here the ESDs of the rates overlap and the *U*_eff_ values are within error.^41^ The presence of three transverse interactions for **3-Dy** is expected to diminish SMM parameters compared to complexes with two monodentate halobenzenes, and this is reflected in its increased calculated transverse *g*-values and lower *U*_eff_ value; these are compensated by a smaller τ_0_ value, leading to similar Orbach rates for **2-Dy**, **3-Dy**, and **4-Dy** (Figure S104).

The predominant relaxation mechanism at intermediate temperature regimes involves Raman processes, invariably promoted by vibrations experienced within the SMMs. Low value Raman exponents such as those observed for **2-Dy**, **3-Dy**, and **4-Dy** have previously been correlated with the absence of equatorial interactions within the rigid {Dy(Cp^R^)_2_} motif.^16^ The variation in Raman rates for **2-Dy**, **3-Dy**, and **4-Dy** is reflected in the prefactor *C*: **4-Dy** < **2-Dy** < **3-Dy**. Faster Raman rates with additional equatorial interactions in **3-Dy** are consistent with conclusions from {Dy(Cp^ttt^)(Cp*)}-haloarene adducts,^41^ while the divergence of **2-Dy** and **4-Dy** rates is not well understood. The impact of variations in transverse field interactions is demonstrated by the differences in QTM tunneling time (τ_QTM_). The resultant QTM rate order of **3-Dy** > **2-Dy** > **4-Dy** is identical to the trend in Raman rates and can be ascribed to the increased transverse interactions in **3-Dy** and tentatively to the longer Dy–X distances in **4-Dy**.

The hysteresis data collected for the halobenzene-bound complexes each display archetypal steps at zero-field, indicative of QTM. The data collected for **2-Dy** (*U*_eff_ = 930(6) cm^–1^, *T*_H_ = 8 K) **3-Dy** (*U*_eff_ = 776(11) cm^–1^, *T*_H_ = 4 K), and **4-Dy** (*U*_eff_ = 921(6) cm^–1^, *T*_H_ = 10 K) are comparable to previously reported {Dy(Cp*)_2_}-containing complexes with equatorial interactions from neutral species (*cf*. [Dy(Cp*)_2_(NH_3_)_2_][BPh_4_]: *U*_eff_ = 546 cm^–1^, *T*_H_ = 5.2 K;^25^ [Dy(Cp*)_2_(μ-OC)W(Cp)(CO)(μ-CO)]_∞_: *U*_eff_ = 557 cm^–1^, *T*_H_ = 1.9 K;^31^ [Dy(Cp*)_2_(μ-OC)FeCp(μ-OC)]_2_: *U*_eff_ = 662 cm^–1^, *T*_H_ = 6.2 K;^23^ [{Dy(Cp*)_2_(μ-Me_3_AlNEt_3_)}_2_][Al{OC(CF_3_)_3_}_4_]_2_: *U*_eff_ = 860 cm^–1^, *T*_H_ = 12 K).^29^ By comparing rates of all reported {Dy(Cp*)_2_}-containing complexes (Figure S105), we find a rough correlation between the charge on equatorial ligands and the Orbach rates. Orbach rates for **2-Dy**, **3-Dy**, and **4-Dy** are slower than these previously reported complexes, while [{Dy(Cp*)_2_(μ-Me_3_AlNEt_3_)}_2_][Al{OC(CF_3_)_3_}_4_]_2_ shows the slowest Raman rates.^29^ The slow Orbach rates for **2-Dy**, **3-Dy**, and **4-Dy** are consistent with their Dy···Cp*_centroid_ distances being shorter than similar complexes with equatorially coordinated neutral ligands, while the Cp*_centroid_···Dy···Cp*_centroid_ angles are comparable, e.g.: [Dy(Cp*)_2_(NH_3_)_2_][BPh_4_] (2.350(3) Å and 140.25(6)°);^25^ [{Dy(Cp*)_2_(μ-Me_3_AlNEt_3_)}_2_][Al{OC(CF_3_)_3_}_4_]_2_ (2.343(3) Å and 138.03(13)°).^29^

It was expected that increasing the number of halobenzene ligands would uniformly make {Dy(Cp*)_2_}-adducts worse-performing SMMs than their {Dy(Cp^ttt^)(Cp*)}^+^ counterparts; however, this is not the case. Complex **3-Dy** shows an improved *T*_H_ value and slightly slower rates (Figure S106) when compared to [Dy(Cp^ttt^)(Cp*)(C_6_H_4_F_2_-κ^2^-*F*,*F*)][Al{OC(CF_3_)_3_}_4_] (*U*_eff_ = 850(13) cm^–1^, where the hysteresis loop is closed around zero field at *T* = 1.8 K).^41^ Comparable rates are attributed to similar {Dy(Cp^R^)_2_}-core geometries for **3-Dy** and the *o*-C_2_H_4_F_2_ adduct of {Dy(Cp^ttt^)(Cp*)}^+^ (Dy···Cp^ttt^_centroid_ = 2.331(3) Å, Dy···Cp*_centroid_ = 2.345(2) Å, Cp^ttt^_centroid_···Dy···Cp*_centroid_ = 144.4(4)°). Complexes **2-Dy**, **4-Dy**, and **5-Dy** are significantly more bent than their corresponding {Dy(Cp^ttt^)(Cp*)}^+^ analogs [Dy(Cp^ttt^)(Cp*)(C_6_H_5_X-κ-*X*)][Al{OC(CF_3_)_3_}_4_] (X = F: 2.307(2) Å, 2.315(2) Å, 147.18(6)°; X = Cl: 2.313(1) Å, 2.315(2) Å, 147.33(5)°; X = Br: 2.317(2) Å, 2.33(2) Å, 149.3(3)°). The bent geometries and increased transverse interactions result in complexes **2-Dy** and **4-Dy** showing the expected faster rates (Figure S106) and reduced *T*_H_ when compared to [Dy(Cp^ttt^)(Cp*)(C_6_H_5_X-κ-*X*)][Al{OC(CF_3_)_3_}_4_] (X = F: *U*_eff_ = 1100(9) cm^–1^, *T*_H_ = 22 K; X = Cl: *U*_eff_ = 1125(12) cm^–1^, *T*_H_ = 24 K).^41^ There is also no evidence of phonon bottleneck behavior in **2-Dy**, **3-Dy**, and **4-Dy**, as was previously observed for [Dy(Cp^ttt^)(Cp*)(PhX-κ-*X*)][Al{OC(CF_3_)_3_}_4_] (X = F, Cl, Br).^41^

Weak equatorial interactions in **2-Dy**, **3-Dy**, and **4-Dy** disrupt the rigidity of the ligand framework and reduce the axiality of the complex to promote relaxation compared to the theoretical [Dy(Cp*)_2_]^+^ cation.^4,62,63^ Calculations on the theoretical complex [Dy(Cp*)_2_][Al{OC(CF_3_)_3_}_4_] predict higher ground state purity (99.4% *m*_J_ = ± 15/2) than **2′-Dy**, **3′-Dy**, **4′-Dy**, and **5′-Dy** and a larger *U*_calc_ = 1284 cm^–1^ (based on state purity). The predicted *U*_eff_ is higher than previously reported for [Dy(Cp*)_2_]^+^ (ca. 1000–1100 cm^–1^),^17,18^ which can be attributed to different geometries used and methods of determining *U*_calc_. In contrast, samples of “[{Dy(Cp*)_2_}{Al[OC(CF_3_)_3_]_4_}]” exhibit rapid QTM and Raman processes that are much faster than **2-Dy**, **3-Dy**, and **4-Dy** and have no observable Orbach relaxation (Figure S106). The hysteresis loops are open at 4 K, but at zero-field, there is no remanent magnetization (coercive field < 50 Oe) in a similar motif to that observed for [Dy(Cp*)_2_{μ-(Ph)_2_BPh_2_}].^21^ Given the anticipated magnetic properties of the isolated [Dy(Cp*)_2_]^+^ cation,^17,18^ it can be concluded with relative confidence that “[{Dy(Cp*)_2_}{Al[OC(CF_3_)_3_]_4_}]” does not contain a separated ion pair and that triethylamine and/or the WCA forms equatorial interactions with the {Dy(Cp*)_2_} fragment. This introduces both static and dynamic disruptions to the uniaxial anisotropy, mixing the ground states and leading to efficient QTM and Raman processes,^64,65^ which contrast with the long relaxation times expected for the free [Dy(Cp*)_2_]^+^ cation.^17^

## Conclusion

We have prepared bis-halobenzene adducts of the {Dy(Cp*)_2_}^+^ fragment, and we have studied their magnetic properties to provide comparisons with the putative [Dy(Cp*)_2_]^+^ cation, which is a longstanding target SMM. We find that C–X (X = F, Cl, Br) bond activation and displacement of halobenzenes by O-donor ligands provide facile decomposition routes for decamethyldysprosocenium cation bis-halobenzene adducts. The SMM parameters measured for [Dy(Cp*)_2_(PhX-κ-*X*)_2_][Al{OC(CF_3_)_3_}_4_] are similar to other complexes containing {Dy(Cp*)_2_}^+^ fragments and weakly coordinating equatorial ligands, though the *U*_eff_ value of 930(6) cm^–1^ measured for the fluorobenzene analog sets a new record for this class of complex. The coordination of two halobenzene molecules to the {Dy(Cp*)_2_}^+^ fragment gives cations with highly bent Cp*···Dy···Cp* angles and significant transverse fields, leading to reduced purity of *m*_J_ states and promoting all relaxation mechanisms. These data confirm that for the [Dy(Cp*)_2_]^+^ cation to be isolated and free of equatorial ligand interactions, a more weakly coordinating counteranion needs to be employed or a significantly different experimental approach needs to be followed, e.g. encapsulation of [Dy(Cp*)_2_]^+^ in an appropriate host–guest matrix.

## Data Availability

Research data files supporting this publication are available from FigShare at 10.6084/m9.figshare.24582711.

## Supporting Information Available

Additional experimental details for physical and computational data (PDF)

## Accession Codes

CCDC 2303545–2303560 contain the supplementary crystallographic data for this paper. These data can be obtained free of charge via www.ccdc.cam.ac.uk/data_request/cif, or by emailing data_request@ccdc.cam.ac.uk, or by contacting The Cambridge Crystallographic Data Centre, 12 Union Road, Cambridge, CB2 1EZ, UK; fax: + 44 1223 336033.
