# Supplementary material for: Synthesis and Magnetic Properties of Bis-Halobenzene Decamethyldysprosocenium Cations
Source: Inorg Chem. 2024 Feb 21;63(21):9562–71. doi: 10.1021/acs.inorgchem.3c04106 (PMC11134500; doi:10.1021/acs.inorgchem.3c04106)
Supplement: Supplementary file 1 — ic3c04106_si_001.pdf [file ic3c04106_si_001.pdf]

Supplementary Information for:

Synthesis and Magnetic Properties of Bis-Halobenzene Decamethyldysprosocenium Cations

Sophie C. Corner, Gemma K. Gransbury, Iñigo J. Vitorica-Yrezabal, George F. S. Whitehead, Nicholas F. Chilton and David P. Mills**

Department of Chemistry, The University of Manchester, Oxford Road, Manchester, M13 9PL, U.K.

Contents

1. General Methods S2

2. Synthesis S3

3. NMR Spectroscopy S9

4. Powder X-ray Diffraction S18

5. Single Crystal X-ray Diffraction S22

6. Infrared Spectroscopy S44

7. DFT Calculations S50

8. Magnetic Measurements S58

9. CASSCF-SO Calculations S94

10. References S109

1. General Methods

All manipulations were performed under argon with rigorous exclusion of oxygen and water using Schlenk line and glovebox techniques. Methylcyclohexane and *n*-hexane were dried by refluxing over potassium or sodium/potassium alloy and were stored over potassium mirrors. Fluorobenzene, *ortho*-difluorobenzene, chlorobenzene, bromobenzene and α,α,α-trifluorotoluene were dried by stirring with CaH_2 overnight and were stored over 4 Å molecular sieves. Toluene was dried over a column charged with alumina and was stored over a potassium mirror. Anhydrous benzene was purchased and was stored over 4 Å molecular sieves. Tetrahydrofuran (THF) was dried over a column charged with alumina and stored over 4 Å molecular sieves. All solvents were degassed before use. For NMR spectroscopy C_4D_8O was dried by refluxing over K, and was vacuum transferred and degassed by three freeze-pump-thaw cycles before use. The reagents $[Y(BH_4)_3(THF)_3]$,[1] $[Dy(BH_4)_3(THF)_3]$,[1] KCp*,[2] $[HNEt_3][Al\{OC(CF_3)_3\}_4]$,[3] $[CPh_3][Al\{OC(CF_3)_3\}_4]$[3] and $[CPh_3][B(C_6F_5)_4]$[4] were synthesized according to literature procedures; all other reagents were purchased from commercial sources and were used as received. ^{1}H (400 and 500 MHz), $^{13}C\{^{1}H\}$ (126 MHz) and ^{11}B (128 MHz**)** NMR spectra were obtained on a Bruker Avance III 400 or 500 MHz spectrometer at 298 K and were referenced to the solvent used, or to external TMS (^{1}H, ^{13}C), H_3BO_3/D_2O (^{11}B). ATR-IR spectra were recorded on a Bruker Alpha spectrometer with Platinum-ATR module. Elemental analysis was carried out by Mr Martin Jennings and Mrs Anne Davies at the Microanalytical service, Department of Chemistry, the University of Manchester.

2. Synthesis

[Y(Cp*)$_2$(*μ*-BH$_4$)]$_\infty$ (1-Y). Toluene (15 mL) was added to a mixture of [Y(BH$_4$)$_3$(THF)$_3$] (0.700 g, 2.0 mmol) and KCp* (0.700 g, 4.0 mmol). The reaction mixture was heated to reflux and stirred for 48 h to afford a colorless solution and a white precipitate. The solution was isolated *via* filtration and the solvent was removed under vacuum to give a light-yellow, semi-crystalline solid. The crude product was heated (150 °C) under vacuum for 5 h to remove the residual solvent; a yellow solid sublimed out to afford a white powder. Hot toluene (200 mL) was used to extract the product **1-Y** which formed as a colorless, microcrystalline material at room temperature (0.420 g, 1.1 mmol, 56%). Crystals of **1-Y** containing 0.5 eq. lattice methylcyclohexane suitable for XRD analysis were obtained by dissolving a small amount of product (100 mg) in hot methylcyclohexane (20 mL) and cooling slowly to room temperature over the course of 1 h. Anal. Calcd for $C_{20}H_{34}BY$: C, 64.19; H, 9.16. Found: C, 63.44; H, 8.73. ^{1}H NMR (400.09 MHz, C_4D_8O, 298 K): δ = 1.93 (s, 30H, Cp-C(C*H*$_3$)), –0.09 (br q, 4H, $^1J_{BH}$ = 85.9 Hz, B*H*$_4$). ^{13}C{^{1}H} NMR (100.60 MHz, C_4D_8O, 298 K): δ = 118.5 (Cp-*C*(CH$_3$)), 12.1 (Cp-C(*C*H$_3$)). ^{11}B{^{1}H} NMR (128.38 MHz, C_4D_8O, 298 K): δ = –24.10 (*B*H$_4$). ^{11}B NMR (128.38 MHz, C_4D_8O, 298 K): δ = –24.12 (p, $^1J_{BH}$ = 85.9 Hz, *B*H$_4$). FTIR (ATR, microcrystalline): $\tilde{\nu}$ = 2970 (w, C–H stretch), 2939 (w, C–H stretch), 2894 (m, C–H stretch), 2857 (w, C–H stretch), 2265 (br. s, B–H stretch), 1490 (m), 1435 (m), 1252 (w), 1167 (br. s), 832 (m), 730 (s) cm^{-1}.

[Dy(Cp*)$_2$(*μ*-BH$_4$)]$_\infty$ (1-Dy). Complex **1-Dy** was synthesized and isolated using analogous reaction and work-up procedures to **1-Y** from [Dy(BH$_4$)$_3$(THF)$_3$] (1.694 g, 4.0 mmol) and KCp* (1.397 g, 8.0 mmol) refluxed in toluene (40 mL). Complex **1-Dy** formed as a yellow microcrystalline powder (0.998 g, 2.2 mmol, 55%). Crystals of **1-Dy** suitable for XRD analysis containing 0.33 eq. lattice methylcyclohexane were obtained by dissolving a small amount of product (100 mg) in hot methylcyclohexane (20 mL) and cooling slowly to room temperature

over the course of 4 h. Anal. Calcd for $C_{20}H_{34}BDy$: C, 53.64; H, 7.65. Found: C, 54.06; H, 7.42. μ_{eff} product = 11.39 μ_B (Evans method, C_4D_8O, 298 K). The paramagnetism of **1-Dy** precluded the assignment of its ^{1}H, $^{13}C\{^{1}H\}$ and $^{11}B\{^{1}H\}$ NMR spectra. FTIR (ATR, microcrystalline): $\tilde{\nu}$ = 2980 (w, C–H stretch), 2892 (m, C–H stretch), 2855 (w, C–H stretch), 2244 (br. s, B–H stretch), 1490 (m), 1428 (m), 1375 (w), 1165 (br. s), 1019 (m), 730 (s), 693 (m) cm^{-1}.

"[{Y(Cp*)₂}{Al[OC(CF₃)₃]₄}]". Complex **1-Y** (0.374 g, 1.0 mmol) was suspended in toluene (15 mL) and $MgCl(C_3H_5)$ (0.6 mL, 1.2 mmol, 2.0 M solution in THF) was added to form an orange solution; the reaction mixture was stirred for 3 h. Volatiles were removed *in vacuo* and a 10:1 *n*-hexane:dioxane solution (2 x 30 mL) was added to triturate; volatiles were then removed *in vacuo*. The crude product was extracted with *n*-hexane (2 x 30 mL), and was dried *in vacuo* to obtain "$[Y(Cp^*)_2(C_3H_5)]$" as a yellow-orange foam (0.349 g, 0.87 mmol, 87%). $[NEt_3H][Al\{OC(CF_3)_3\}_4]$ (0.895 g, 0.84 mmol, 0.95 equivalents) and benzene (40 mL) were added and the reaction mixture was stirred overnight; an orange oil in a light yellow solution was obtained. After filtration, the oil was dried *in vacuo* and washed with benzene (2 x 30 mL) and *n*-hexane (2 x 40 mL); the crude products were dried under vacuum at each stage of the washing process in attempts to remove trimethylamine or azeotropic solutions thereof. Volatiles were removed under vacuum to afford "$[\{Y(Cp^*)_2\}\{Al[OC(CF_3)_3]_4\}]$" as a light yellow powder (0.725 g, 0.52 mmol, 62%, 52% global yield). Elemental analysis and NMR spectroscopy experiments indicated that the product is contaminated with triethylamine in approximately 1:0.75 proportions. Anal. Calcd for $C_{40.5}H_{41.25}AlF_{36}N_{0.75}O_4Y$: C, 34.69; H, 2.97; N, 0.75. Found: C, 33.10; H, 3.02; N, 0.71. ^{1}H NMR (400.09 MHz, C_6H_5F with a CD_2Cl_2 insert, 298 K): δ = 2.40 (q, 4.5H, NCH_2CH_3), 1.71 (s, 30H, Cp-C(CH_3)), 0.89 (t, 6.75H, NCH_2CH_3). $^{13}C\{^{1}H\}$ NMR (100.60 MHz, C_6H_5F with a CD_2Cl_2 insert, 298 K):δ = 120.0 (Cp-*C*(CH_3)), 46.1 (N*C*H_2CH_3), 10.1 (NCH_2*C*H_3), 9.5 (Cp-C(*C*H_3)). ^{19}F NMR (376.46 MHz, C_6H_5F with a

CD_2Cl_2 insert, 298 K): δ = –75.3 ([Al{OC(C*F*$_3$)$_3$}$_4$]$^-$), –113.6 (C_6H_5*F*). FTIR (ATR, microcrystalline): $\tilde{\nu}$ = 2916 (w, C–H stretch), 2869 (w, C–H stretch), 1457 (w), 1385 (w), 1354 (s, C–O stretch), 1294 (s), 1272 (s, C–F stretch), 1237 (s), 1208 (s), 1165 (s), 1087 (w), 1021 (m), 970 (s), 859 (m), 828 (s, Al–O stretch), 725 (s), 561 (s), 536 (s), 442 (s) cm^{-1}.

"[{Dy(Cp*)$_2$}{Al[OC(CF$_3$)$_3$]$_4$}]". The title complex was prepared by following analogous synthetic and work-up procedures to "[{Y(Cp*)$_2$}{Al[OC(CF$_3$)$_3$]$_4$}]" from **1-Dy** (0.716 g, 1.6 mmol) and MgCl(C_3H_5) (0.8 mL, 1.8 mmol, 2.0 M solution in THF) to afford "[Dy(Cp*)$_2$(C_3H_5)]" as a yellow-orange foam (0.703 g, 1.48 mmol, 93%), this was reacted *in situ* with [NEt$_3$H][Al{OC(CF$_3$)$_3$}$_4$] (1.538 g, 1.41 mmol, 0.95 equivalents). Complex "[{Dy(Cp*)$_2$}{Al[OC(CF$_3$)$_3$]$_4$}]" was obtained as a yellow powder (1.479 g, 1.00 mmol, 71%, 63% global yield). Characterization data is given assuming the presence of 0.75 equivalents of triethylamine as a contaminant. Anal. Calcd for $C_{40.5}H_{41.25}AlDyF_{36}N_{0.75}O_4$: C, 32.96; H, 2.82; N, 0.71. Found: C, 31.22; H, 2.79; N, 0.62. The paramagnetism of "[{Dy(Cp*)$_2$}{Al[OC(CF$_3$)$_3$]$_4$}]" precluded the assignment of its ^{1}H and ^{13}C{^{1}H} spectra. ^{19}F NMR (376.46 MHz, C_6H_5F, 298 K): δ = –78.7 ($\upsilon_{1/2}$ ~ 160 Hz, [Al{OC(C*F*$_3$)$_3$}$_4$]$^-$), –134.9 ($\upsilon_{1/2}$ ~ 2840 Hz, C_6H_5*F*). FTIR (ATR, microcrystalline): $\tilde{\nu}$ = 2914 (w, C–H stretch), 2867 (w, C–H stretch), 1457 (w), 1389 (w), 1350 (s, C–O stretch), 1294 (s), 1272 (s, C–F stretch), 1235 (s), 1212 (s), 1163 (s), 1083 (w), 1019 (w), 970 (s), 853 (w), 832 (s, Al–O stretch), 723 (s), 559 (s), 534 (s), 442 (s) cm^{-1}.

[Dy(Cp*)$_2$(PhF-κ-*F*)$_2$][Al{OC(CF$_3$)$_3$}$_4$] (2-Dy). For **2-Dy**, fluorobenzene (2 mL) was used to dissolve "[{Dy(Cp*)$_2$}{Al[OC(CF$_3$)$_3$]$_4$}]" (0.560 g, 0.38 mmol); the solution was filtered and layered with *n*-hexane (20 mL); yellow crystals of **2-Dy** formed after storage at –30 °C for one week (0.605 g, 0.38 mmol, 99%). Anal. Calcd for $C_{48}H_{40}AlDyF_{38}O_4$: C, 36.21; H, 2.53. Found: C, 32.95; H, 2.30. The paramagnetism of **2-Dy** precluded the assignment of its ^{1}H and ^{13}C{^{1}H} NMR spectra. ^{19}F NMR (376.46 MHz, C_6H_5F, 298 K): δ = –78.9 ($\upsilon_{1/2}$ ~ 140 Hz,

[Al{OC(C*F*$_3$)$_3$}$_4$]$^-$), –131.1 ($\upsilon_{1/2}$ ~ 2470 Hz, C_6H_5*F*). FTIR (ATR, microcrystalline): $\tilde{\nu}$ = 2961 (w, C–H stretch), 2916 (w, C–H stretch), 2865 (w, C–H stretch), 1580 (w), 1486 (s, aromatic C=C bend), 1350 (s, C–O stretch), 1274 (s, C–F stretch), 1212 (s), 1165 (s), 1124 (s, C–F stretch), 970 (s), 830 (s, Al–O stretch), 781 (s, C–F stretch), 746 (s), 725 (s), 561 (s), 536 (s), 470 (w), 442 (s) cm^{-1}.

[Y(Cp*)$_2$(PhF-κ-*F*)$_2$][Al{OC(CF$_3$)$_3$}$_4$] (2-Y). Fluorobenzene (0.5 mL) was added to a mix of **1-Y** (19 mg, 0.05 mmol) and [CPh$_3$][Al{OC(CF$_3$)$_3$}$_4$] (60 mg, 0.05 mmol), layering with *n*-hexane (3 mL) led to a small amount of crystals of **2-Y**. Due to the low yield of **2-Y** no further characterization data could be obtained.

[Dy(Cp*)$_2$(C$_6$H$_4$F$_2$-κ^2-*F,F*)(C$_6$H$_4$F$_2$-κ-*F*)][Al{OC(CF$_3$)$_3$}$_4$] (3-Dy). For **3-Dy**, *ortho*-difluorobenzene (2 mL) was used to dissolve "[{Dy(Cp*)$_2$}{Al[OC(CF$_3$)$_3$]$_4$}]" (0.560 g, 0.38 mmol) and the solution was filtered and layered with *n*-hexane (20 mL); yellow crystals of **3-Dy** formed after storage at –30 °C for one week (0.612 g, 0.38 mmol, 94%). Anal. Calcd for $C_{48}H_{38}AlDyF_{40}O_4$: C, 35.41; H, 2.35. Found: C, 31.81; H, 2.24. The paramagnetism of **3-Dy** precluded the assignment of its ^{1}H and ^{13}C{^{1}H} NMR spectra. ^{19}F NMR (376.46 MHz, $C_6H_4F_2$, 298 K): δ = –72.8 ($\upsilon_{1/2}$ ~ 120 Hz, [Al{OC(C*F*$_3$)$_3$}$_4$]$^-$), –153.8 ($\upsilon_{1/2}$ ~ 1410 Hz, C$_6$H$_4$*F*$_2$). FTIR (ATR, microcrystalline): $\tilde{\nu}$ = 2918 (w, C–H stretch), 2871 (w, C–H stretch), 1623 (w), 1494 (s, aromatic C=C bend), 1350 (s, C–O stretch), 1270 (s, C–F stretch), 1212 (s), 1167 (s), 1089 (m, C–F stretch), 970 (s), 834 (s, Al–O stretch), 754 (s, C–F stretch), 725 (s), 561 (s), 536 (s), 442 (s) cm^{-1}.

[Dy(Cp*)$_2$(PhCl-κ-*Cl*)$_2$][Al{OC(CF$_3$)$_3$}$_4$] (4-Dy). For **4-Dy**, chlorobenzene (2 mL) was used to dissolve "[{Dy(Cp*)$_2$}{Al[OC(CF$_3$)$_3$]$_4$}]" (0.560 g, 0.38 mmol), the solution was filtered and layered with *n*-hexane (20 mL); yellow crystals of **4-Dy** containing 0.5 eq. lattice chlorobenzene formed after storage at –30 °C for one week (0.572 g, 0.34 mmol, 89%). Anal.

Calcd for $C_{48}H_{40}AlCl_2DyF_{36}O_4$: C, 35.47; H, 2.48. Found: C. 30.14; H, 2.25. The paramagnetism of **4-Dy** precluded the assignment of its ^{1}H and ^{13}C{^{1}H} NMR spectra. ^{19}F NMR (376.46 MHz, C_6H_5Cl, 298 K): δ = –76.6 ($\upsilon_{1/2}$ ~ 220 Hz, $[Al\{OC(C\mathit{F}_3)_3\}_4]^-$). FTIR (ATR, microcrystalline): $\tilde{\nu}$ = 2982 (w, C–H stretch), 2912 (w, C–H stretch), 2869 (w, C–H stretch), 1574 (w), 1476 (m, aromatic C=C bend), 1350 (s, C–O stretch), 1274 (s, C–F stretch), 1212 (s), 1167 (s), 1060 (w, C–Cl stretch), 1019 (m), 970 (s), 834 (s, Al–O stretch), 746 (s), 725 (s), 686 (s, C–Cl stretch), 561 (s), 536 (s), 442 (s) cm^{-1}.

$[Dy(Cp^*)_2(PhBr\text{-}\kappa\text{-}\mathit{Br})_2][Al\{OC(CF_3)_3\}_4]$ (5-Dy). For **5-Dy**, bromobenzene (2 mL) was used to dissolve "$[\{Dy(Cp^*)_2\}\{Al[OC(CF_3)_3]_4\}]$", the solution was filtered and layered with *n*-hexane (20 mL); yellow crystals of **5-Dy** formed after storage at –30 °C for one week. It was found that crystals of the side products $[\{Dy(Cp^*)_2\}_2(\mu\text{-Br})][Al\{OC(CF_3)_3\}_4]$ (**8-Dy**) and $[\{Dy(Cp^*)_2(PhBr\text{-}\kappa\text{-}\mathit{Br})\}_2(\mu\text{-Br})][Al\{OC(CF_3)_3\}_4]$ (**9-Dy**) or $[Dy(Cp^*)_2(Ph\text{-}\kappa\text{-}\mathit{Br})(THF)][Al\{OC(CF_3)_3\}_4]$ (**11-Dy)** were also present in multiple recrystallization attempts. Due to the presence of multiple products within these samples, further characterization of **5-Dy** was not possible.

$[\{Dy(Cp^*)_2\}_2(\mu\text{-F})][Al\{OC(CF_3)_3\}_4]$ (6-Dy). Complex **6-Dy** was obtained as yellow crystals following the addition of benzene (2 mL), toluene (2 mL) or α,α,α-trifluorotoluene (2 mL) to "$[\{Dy(Cp^*)_2\}\{Al[OC(CF_3)_3]_4\}]$" and subsequent layering with *n*-hexane (20 mL). Due to the contamination of the product formed from the C–F activation of the $[Al\{OC(CF_3)_3\}_4]^-$ anion within the sample, further characterization was not possible. On one attempt, several crystals of $[NEt_3(CF_2C_6H_5)][Al\{OC(CF_3)_3\}_4]$ (**7**) were also obtained from this mixture. Due to the low yield of **7** no further characterization data could be obtained.

$[Dy(Cp^*)_2(DME)][Al\{OC(CF_3)_3\}_4]$ (10-Dy). Several yellow crystals of the complex **10-Dy** formed concurrently with **2-Dy** in a preliminary recrystallization. It was found that a trace

amount of DME was present in the *n*-hexane used to layer the fluorobenzene. Due to the low yield of **10-Dy** no further characterization data could be obtained.

[Dy(Cp*)$_2$(THF)$_2$][Al{OC(CF$_3$)$_3$}$_4$] (12-Dy). THF (1 mL) was used to extract a small portion of "[{Dy(Cp*)$_2$}{Al[OC(CF$_3$)$_3$]$_4$}]" (< 50 mg); the resulting yellow solution was layered with *n*-hexane (10 mL) and stored at –30 °C to give several yellow crystals of **12-Dy**. Due to the low yield of **12-Dy** no further characterization data could be obtained.

[{Dy(Cp*)$_2$(PhF-κ-*F*)}$_2$(*μ*-BH$_4$)][Al{OC(CF$_3$)$_3$}$_4$] (13-Dy). Fluorobenzene (10 mL) was added to a mixture of **1-Dy** (0.224g, 0.5 mmol) and [CPh$_3$][Al{OC(CF$_3$)$_3$}$_4$] (0.603 g, 0.5 mmol). The reaction mixture was stirred for 16 h to form a yellow solution and white precipitate. After solids were removed *via* filtration, the solution was concentrated (*ca*. 2 mL) and layered with *n*-hexane (20 mL). Yellow crystals of **2-Dy** and **13-Dy** formed concurrently after one week at room temperature. Due to the presence of multiple complexes within the sample, further characterization was not possible.

[Y(Cp*)$_2${B(C$_6$F$_5$)$_4$-κ-*F*}(PhF-κ-*F*)] (14-Y). Fluorobenzene (10 mL) was added to **1-Y** (0.212 g, 0.5 mmol) and [(Et$_3$Si)$_2$(*μ*-H)][B(C$_6$F$_5$)$_4$] (0.456 g, 0.5 mmol) and the mixture was stirred for 16 h. Volatiles were removed *in vacuo*, the crude product was washed with *n*-hexane (2 x 10 mL) and fluorobenzene (2 mL) was added to re-dissolve the product. After filtration to remove residual solids, the solution was layered with *n*-hexane (15 mL) and stored at room temperature to afford several colorless crystals of **14-Y**. Due to the low yield of **14-Y** no further characterization data could be obtained.

3. NMR Spectroscopy

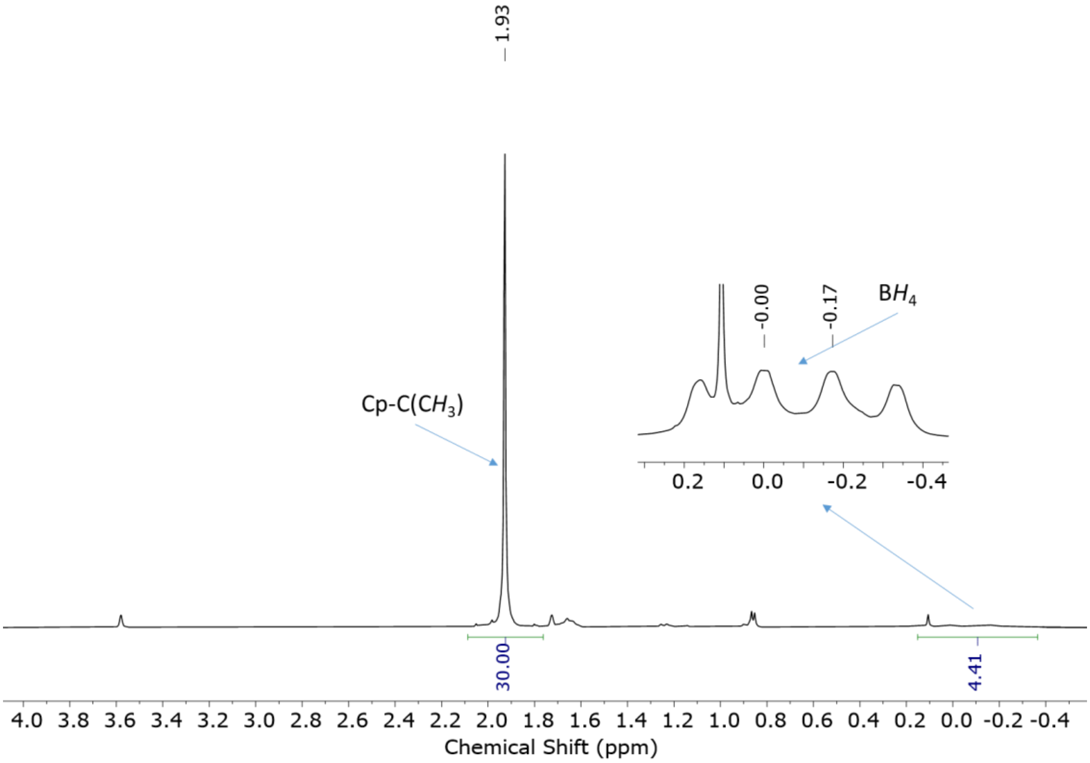

Figure S1. ^{1}H NMR spectrum of **1-Y** (400 MHz) in C_4D_8O.

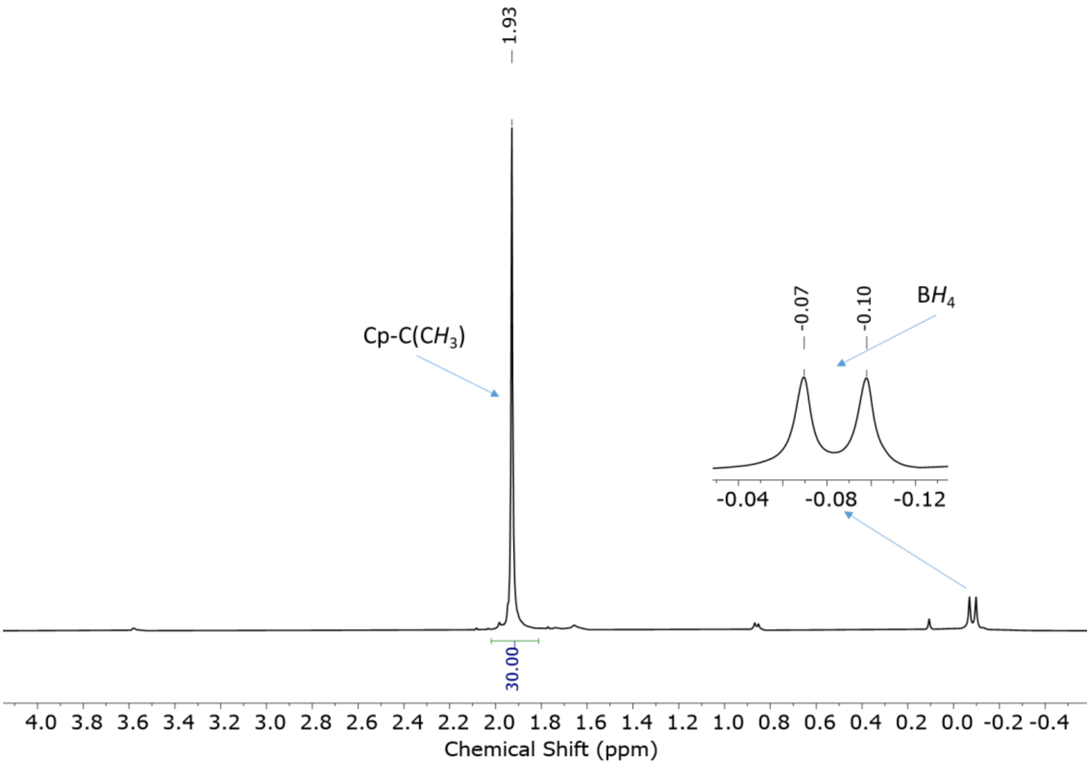

Figure S2. $^{1}H\{^{11}B\}$ NMR spectrum of **1-Y** (400 MHz) in C_4D_8O.

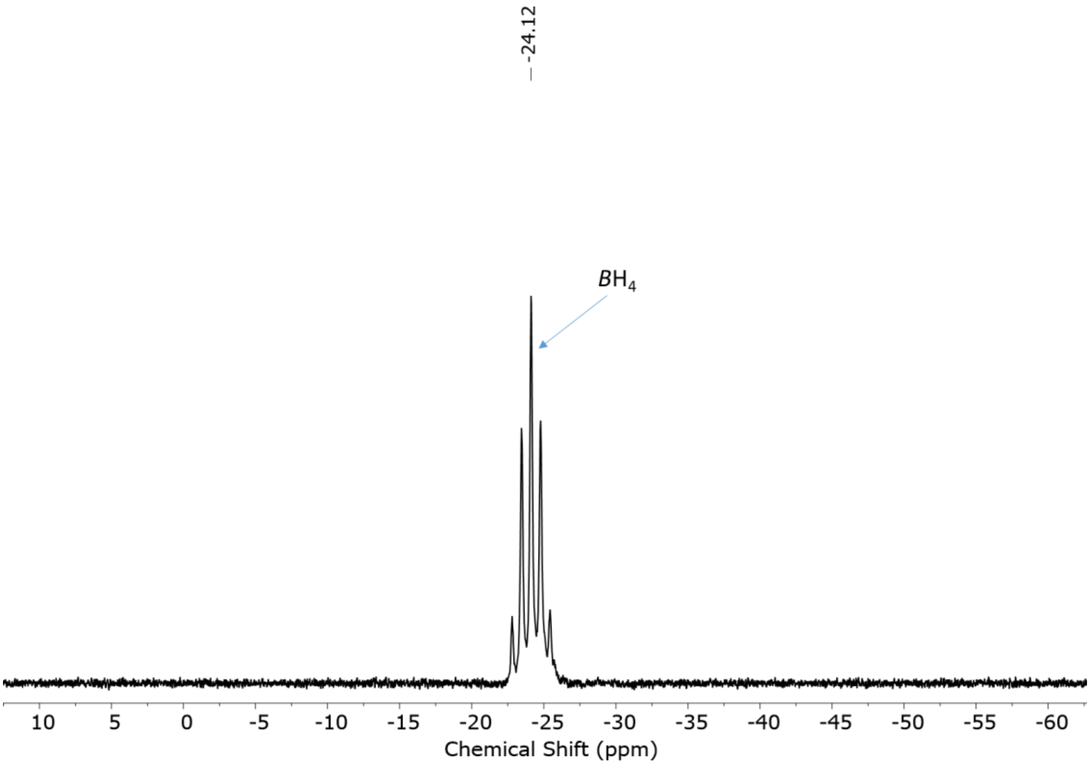

Figure S3. ^{11}B NMR spectrum of **1-Y** (128 MHz) in C_4D_8O.

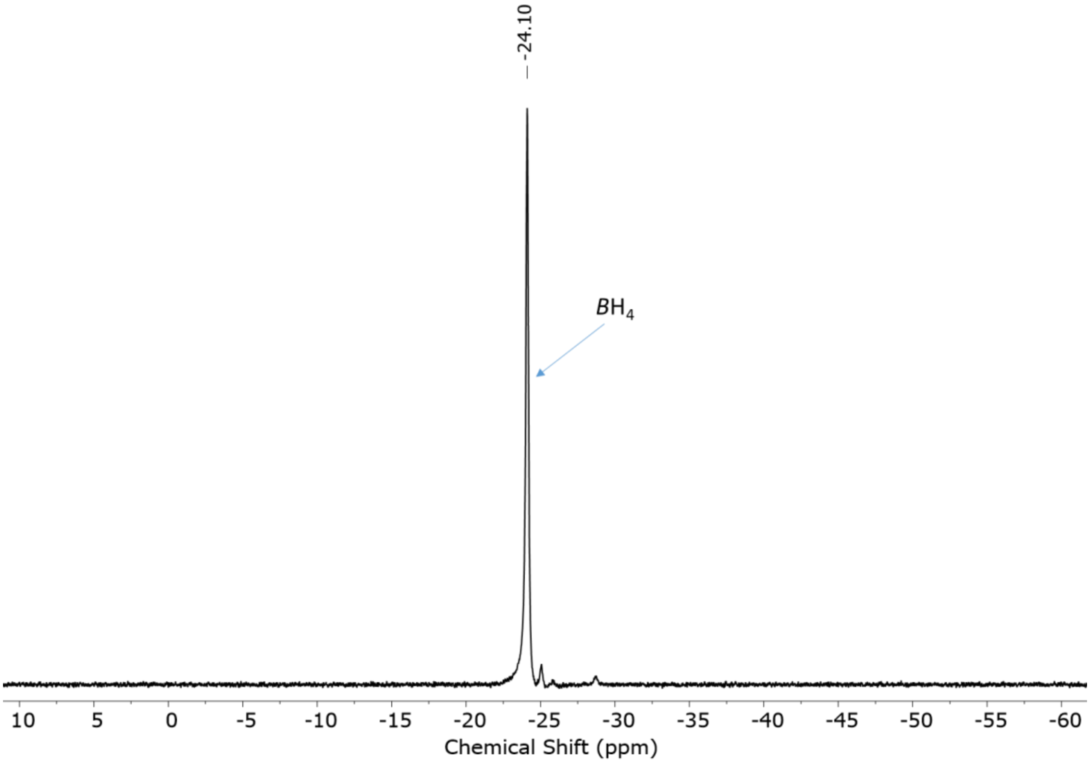

Figure S4. $^{11}B\{^{1}H\}$ NMR spectrum of **1-Y** (128 MHz) in C_4D_8O.

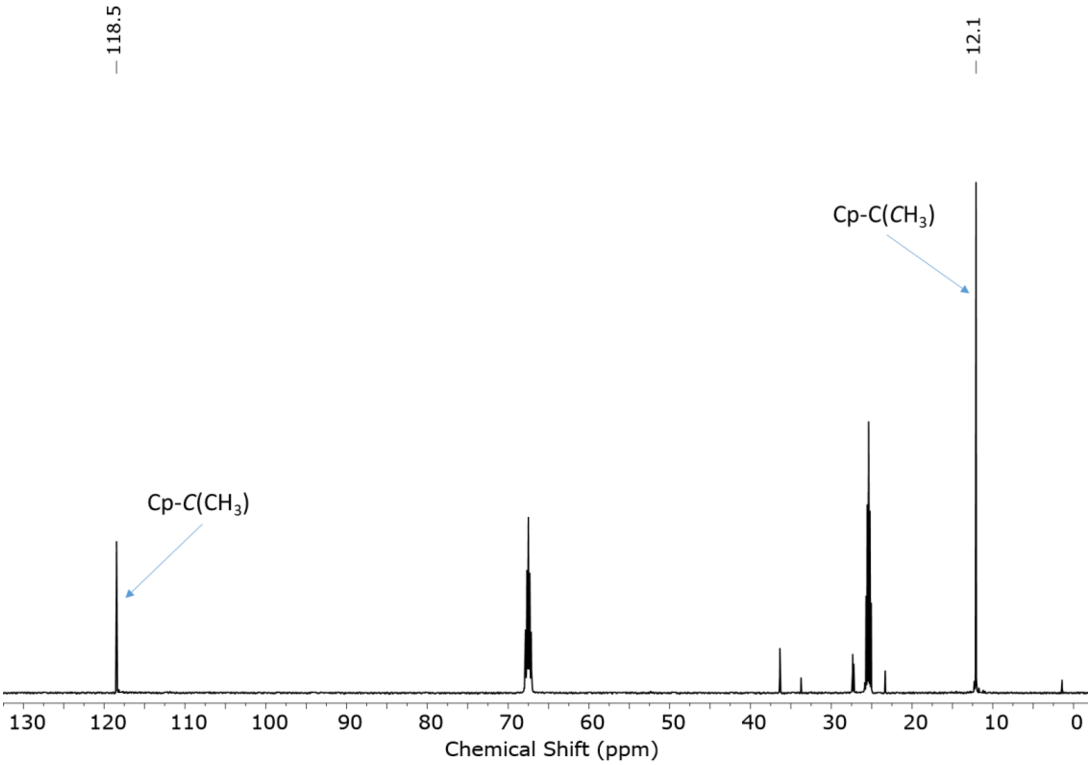

Figure S5. $^{13}C\{^{1}H\}$ NMR spectrum of **1-Y** (126 MHz) in C_4D_8O.

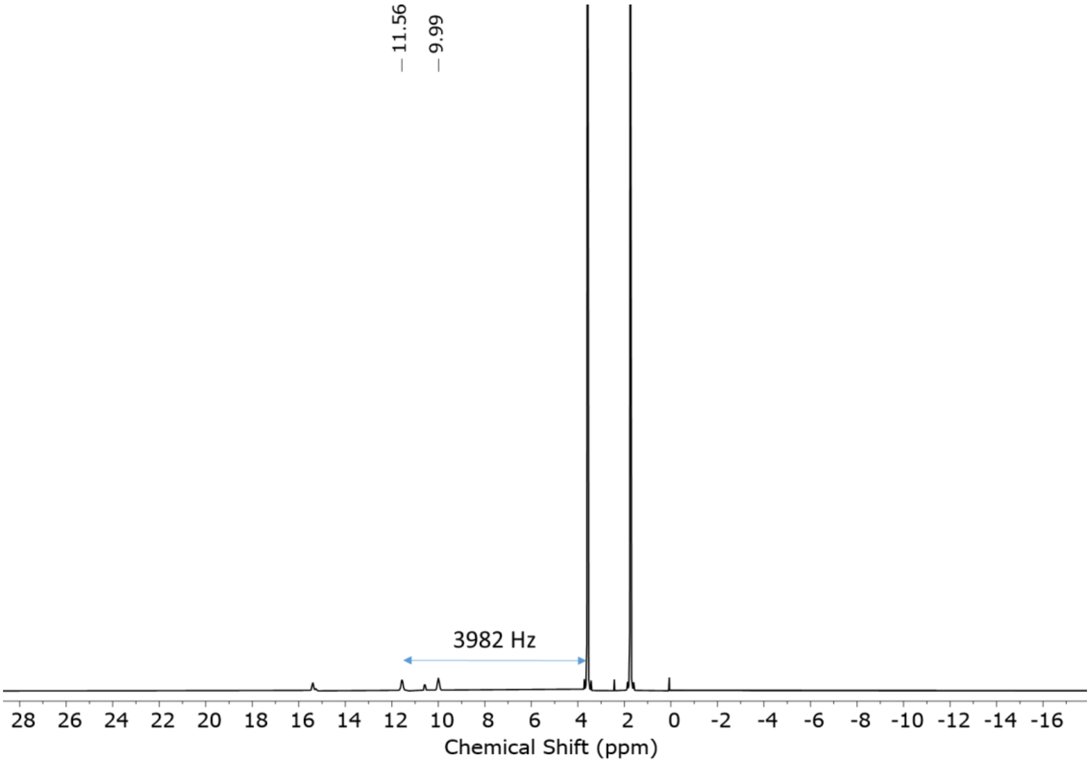

Figure S6. ^{1}H NMR spectrum of **1-Dy** (500 MHz) in C_4D_8O with a C_4D_8O insert; full spectral range 200 to –200 ppm.

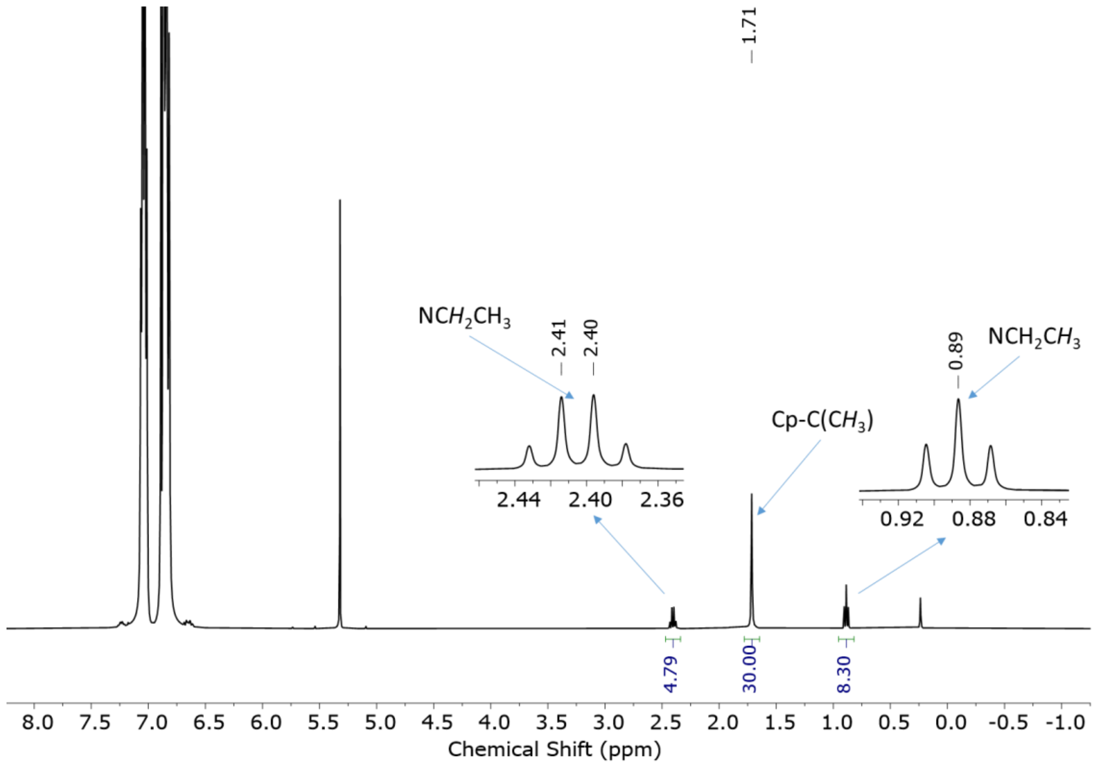

Figure S7. ^{1}H NMR spectrum of "[{Y(Cp*)$_2$}{Al[OC(CF$_3$)$_3$]$_4$}]" (400 MHz) in C_6H_5F with a CD_2Cl_2 insert.

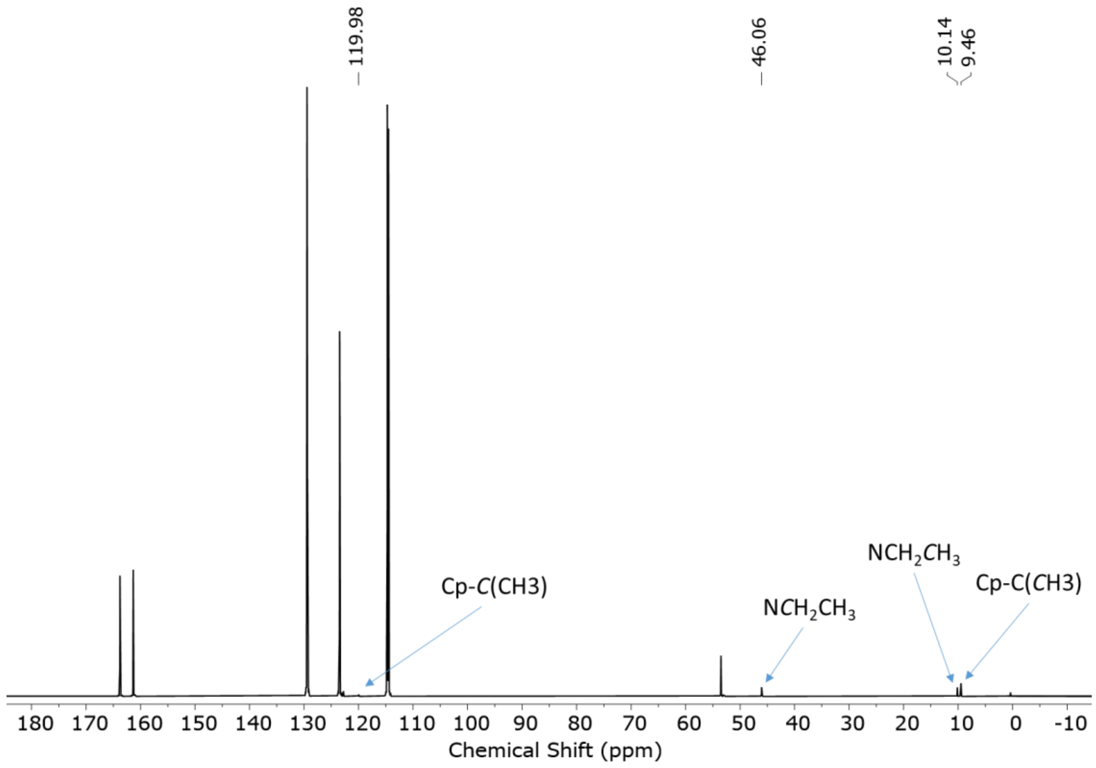

Figure S8. ^{13}C{^{1}H} NMR spectrum of "[{Y(Cp*)$_2$}{Al[OC(CF$_3$)$_3$]$_4$}]" (126 MHz) in C_6H_5F with a CD_2Cl_2 insert.

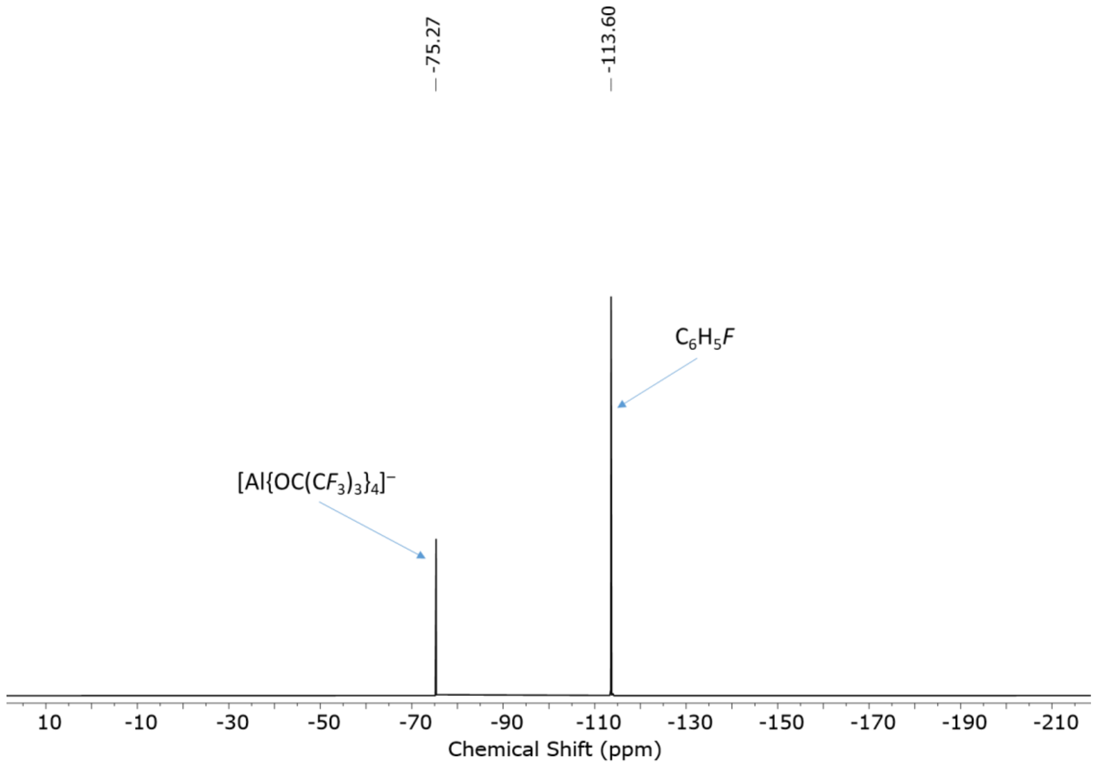

Figure S9. ^{19}F NMR spectrum of "[{Y(Cp*)$_2$}{Al[OC(CF$_3$)$_3$]$_4$}]" (376 MHz) in C_6H_5F with a CD_2Cl_2 insert.

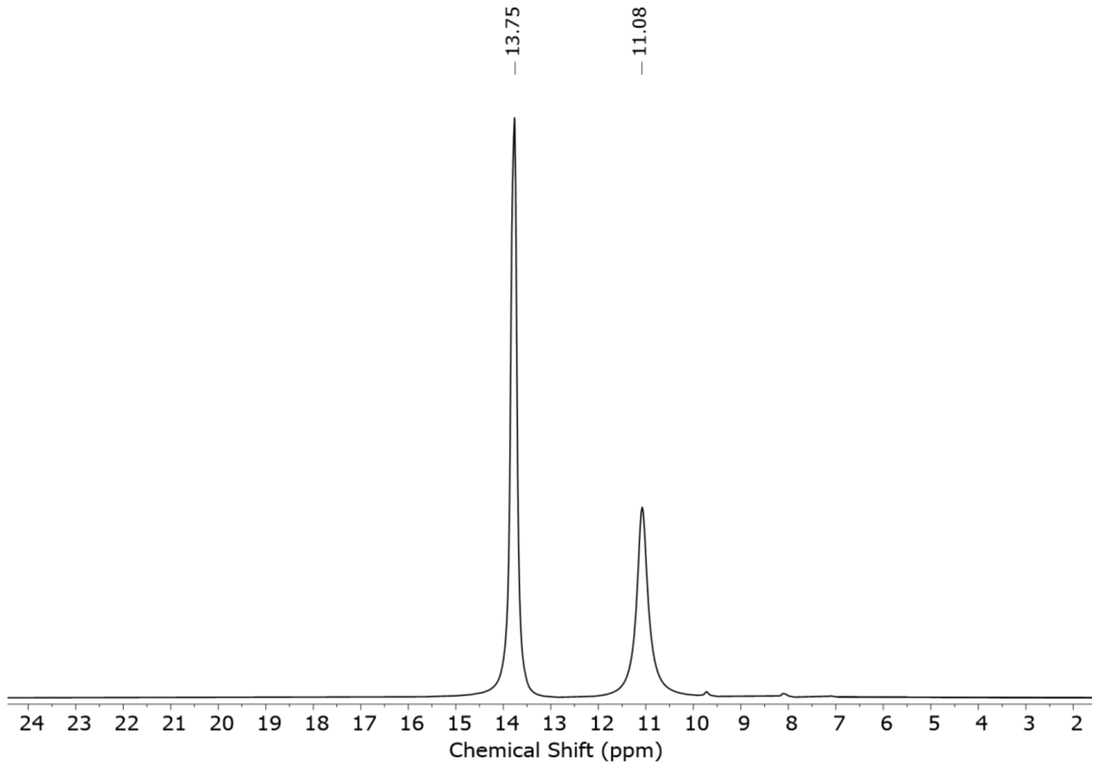

Figure S10. ^{1}H NMR spectrum of "[{Dy(Cp*)$_2$}{Al[OC(CF$_3$)$_3$]$_4$}]" (400 MHz) in C_6H_5F.

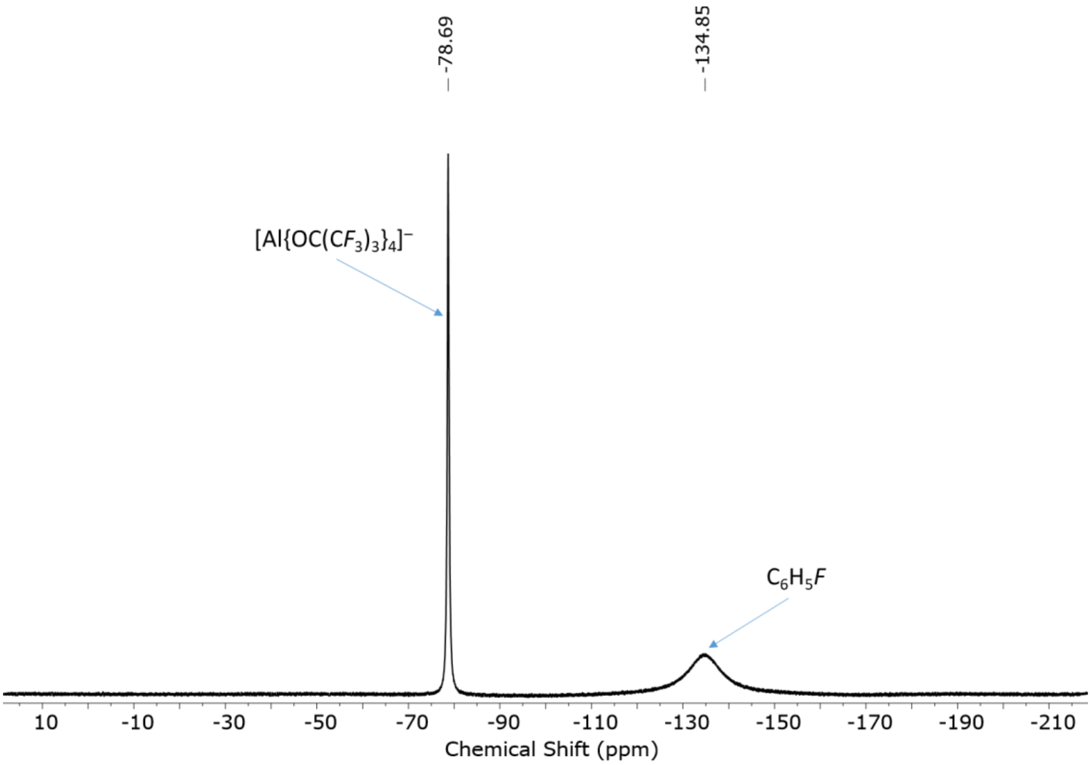

Figure S11. ^{19}F NMR spectrum of "[{Dy(Cp*)$_2$}{Al[OC(CF$_3$)$_3$]$_4$}]" (376 MHz) in C_6H_5F.

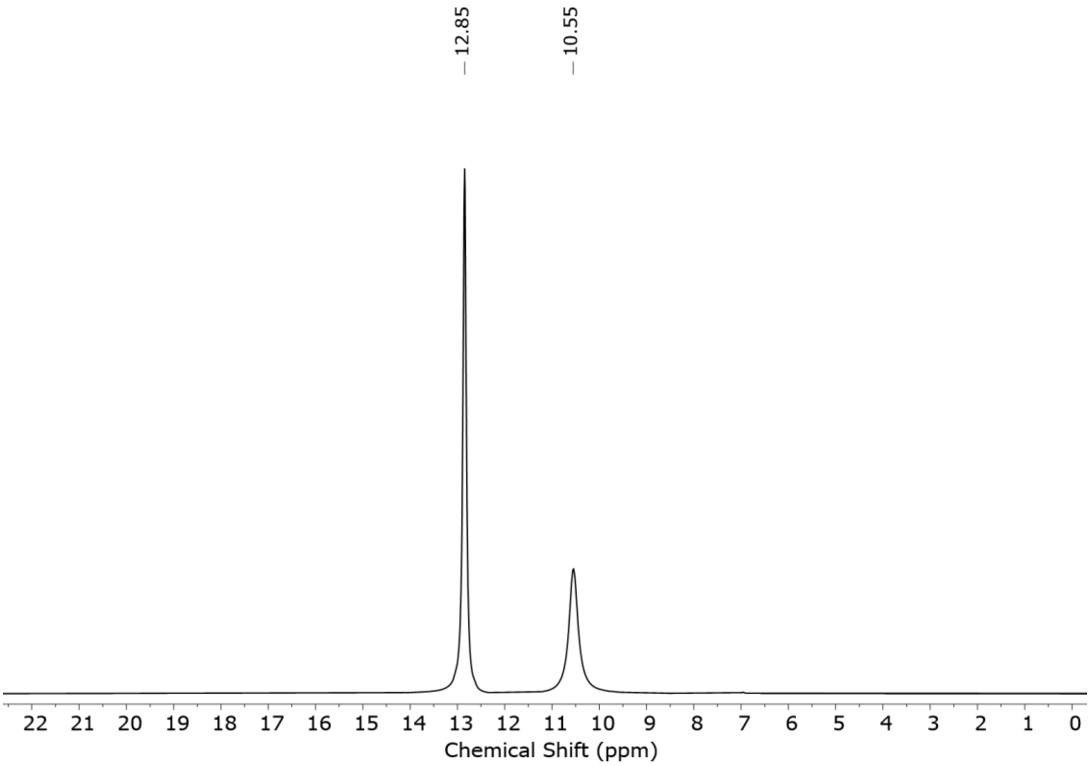

Figure S12. ^{1}H NMR spectrum of **2-Dy** (400 MHz) in C_6H_5F; full spectral range 200 to –200 ppm.

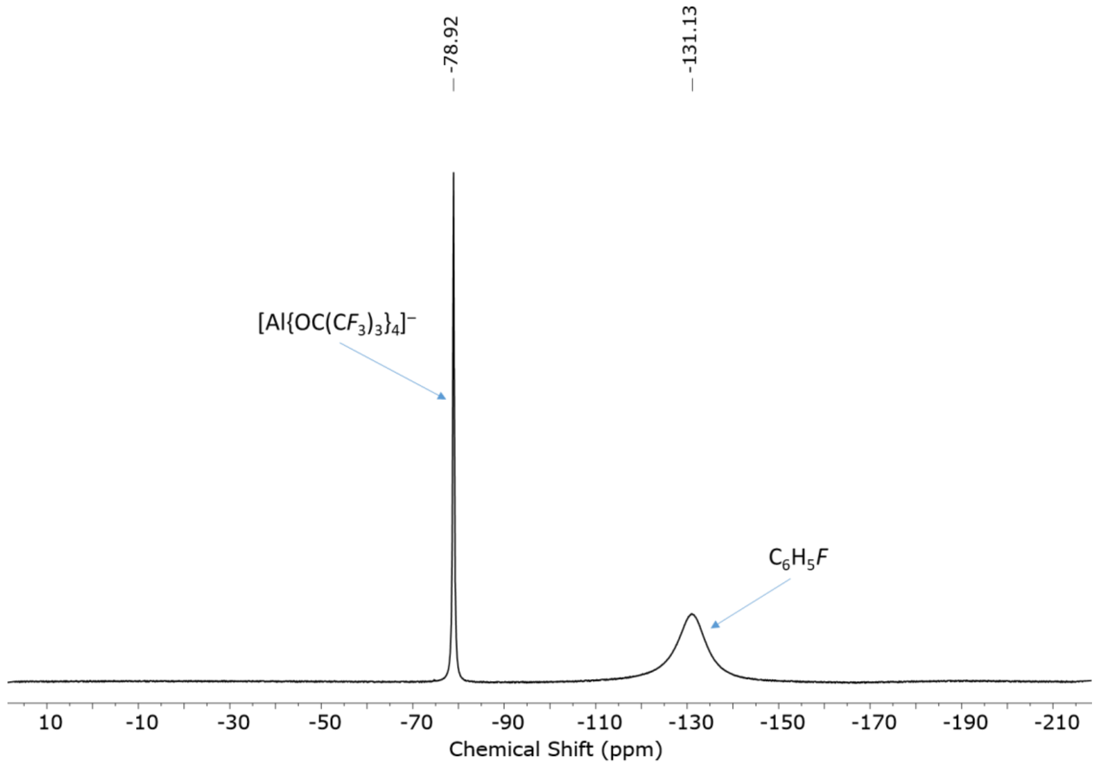

Figure S13. ^{19}F NMR spectrum of **2-Dy** (376 MHz) in C_6H_5F.

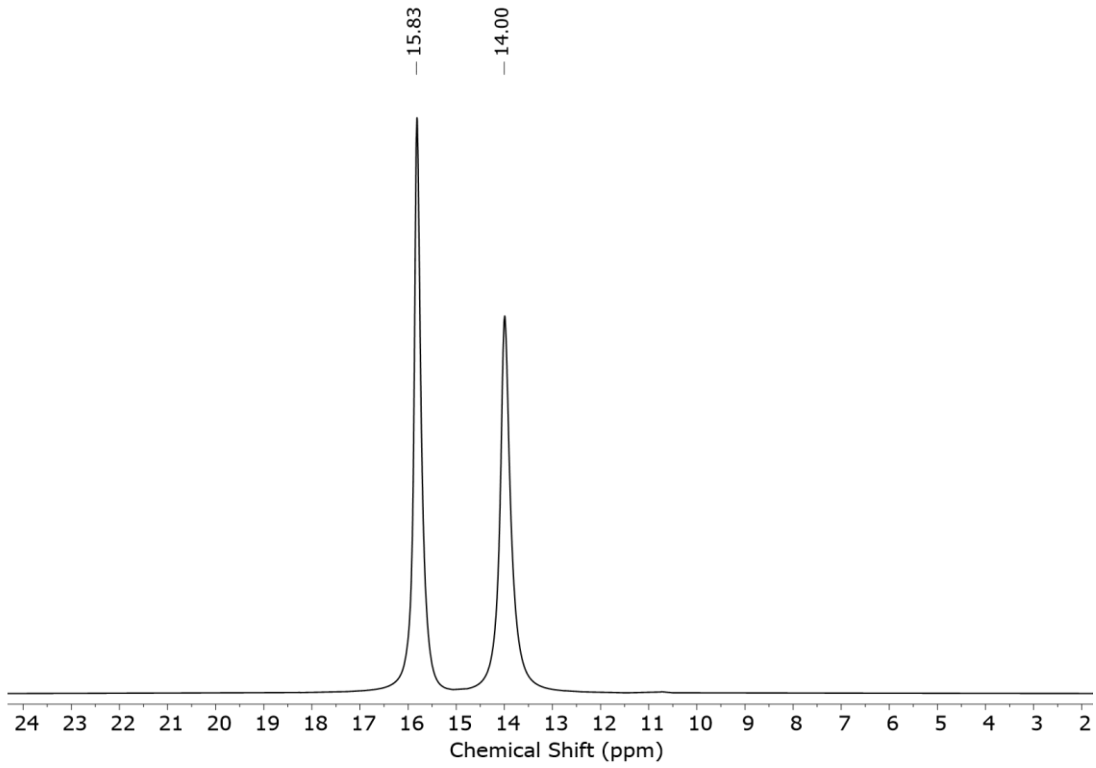

Figure S14. ^{1}H NMR spectrum of **3-Dy** (400 MHz) in $C_6H_4F_2$; full spectral range 200 to –200 ppm.

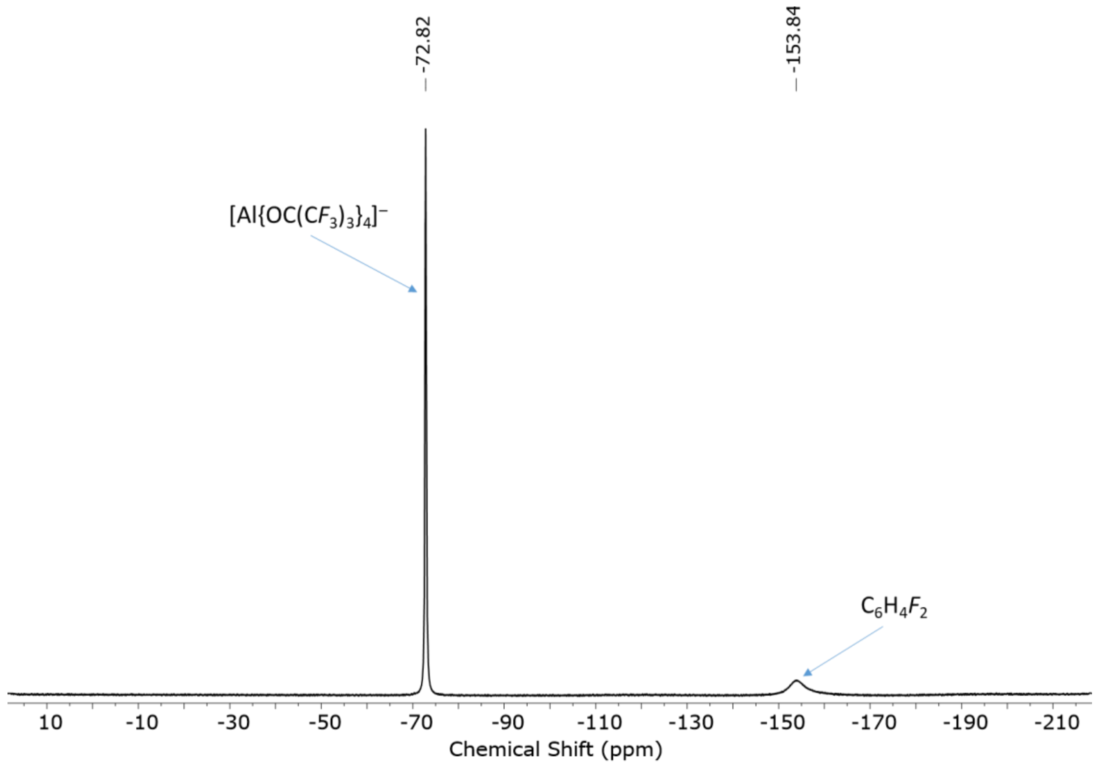

Figure S15. ^{19}F NMR spectrum of **3-Dy** (376 MHz) in $C_6H_4F_2$.

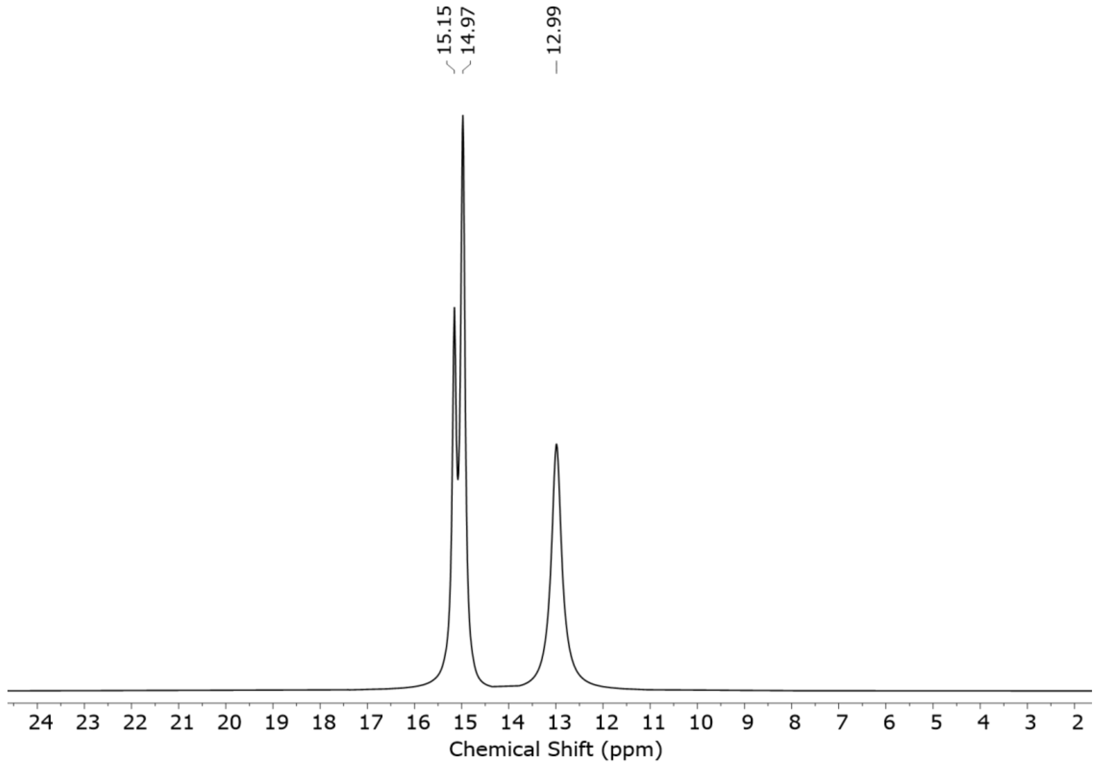

Figure S16. ^{1}H NMR spectrum of **4-Dy** (400 MHz) in C_6H_5Cl; full spectral range 200 to –200 ppm.

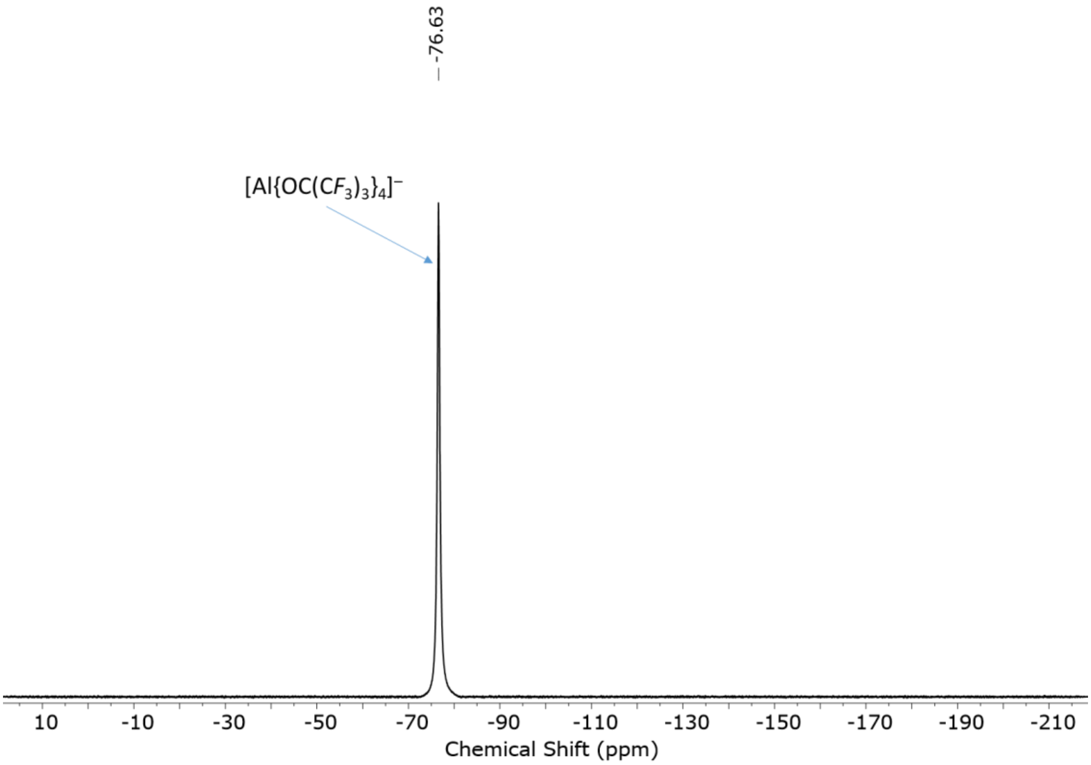

Figure S17. ^{19}F NMR spectrum of **4-Dy** (376 MHz) in C_6H_5Cl.

4. Powder X-ray Diffraction

Data collection. X-ray diffraction data of microcrystalline samples of **2-Dy**, **3-Dy** and **4-Dy** mounted with a minimum amount of fomblin were collected at 100 K using a Rigaku FR-X rotating anode single crystal X-ray diffractometer using Cu Kα radiation (λ = 1.5418 Å) with a Hypix-6000HE detector and an Oxford Cryosystems nitrogen flow gas system. Data were collected between 3–70 °θ, with a detector distance of 150 mm and a beam divergence of 1.5 mRad[5] X-ray data were collected using CrysAlisPro software.[6]

Data processing. The instrument was calibrated using silver behenate as standard. Then, X-ray data were reduced and integrated using CrysAlisPro software.[6] Pawley refinement with the unit cells obtained from the crystal structures were performed using TOPAS software.[7,8]

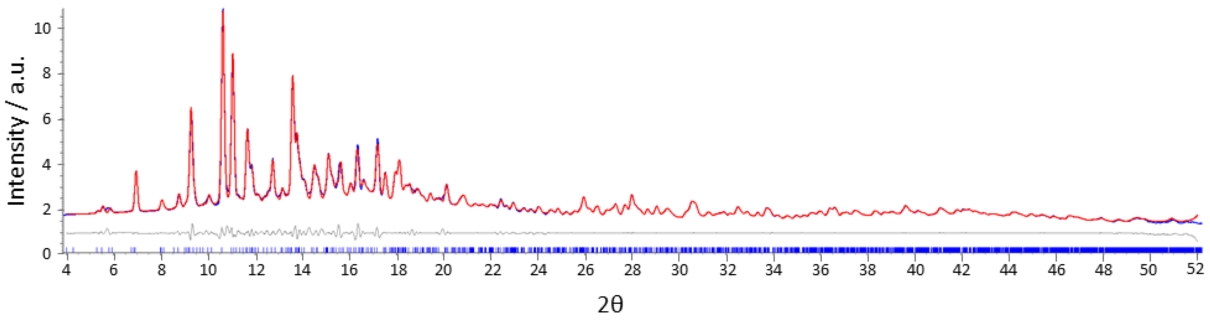

Figure S18. Pawley refinement analysis of **2-Dy**; experimental data (blue), calculated model from crystallographic parameters (red) and the difference (gray). Pawley refinement Rwp = 3.323; Rwp′ = 10.235. Pawley refinement was determined using the unit cell values from the crystal structure.

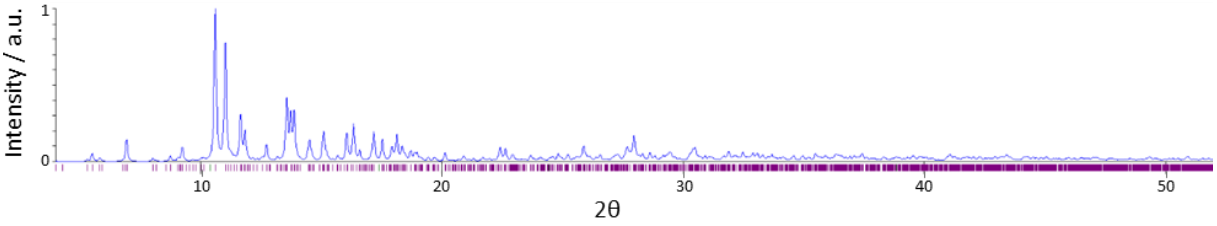

Figure S19. Theoretical powder X-ray diffraction pattern of **2-Dy** derived from crystallographic parameters.

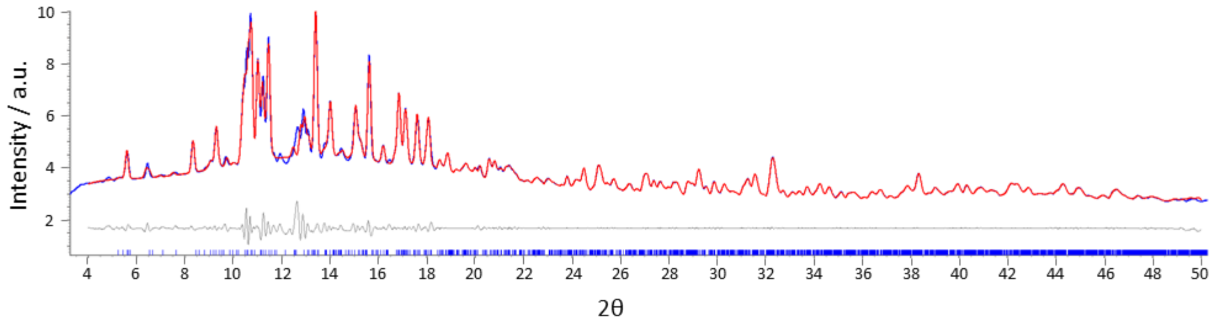

Figure S20. Pawley refinement analysis of **3-Dy**; experimental data (blue), calculated model from crystallographic parameters (red) and the difference (gray). Pawley refinement Rwp = 2.315; Rwp′ = 5.629. Pawley refinement was determined using the unit cell values from the crystal structure.

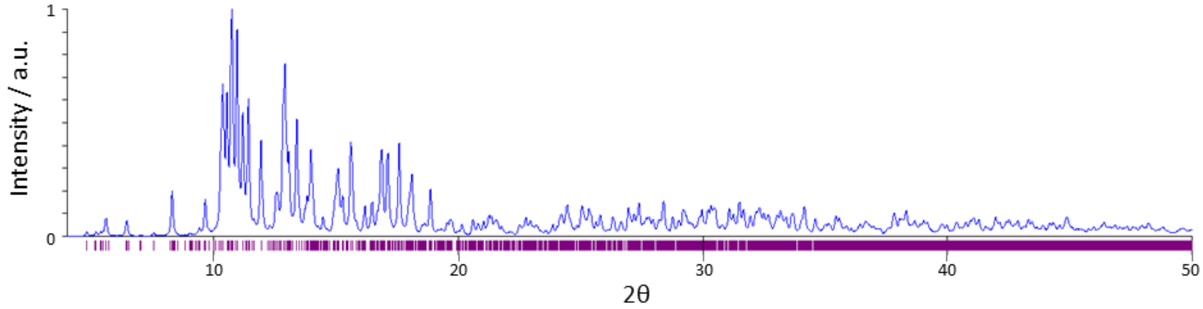

Figure S21. Theoretical powder X-ray diffraction pattern of **3-Dy** derived from crystallographic parameters.

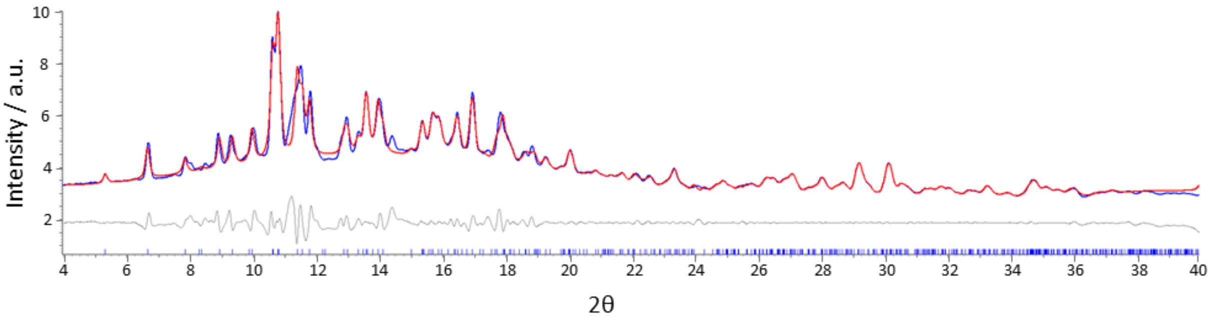

Figure S22. Pawley refinement analysis of **4-Dy**; experimental data (blue), calculated model from crystallographic parameters (red) and the difference (gray). Pawley refinement Rwp = 3.800; Rwp′ = 17.009. Pawley refinement was determined using the unit cell values from the crystal structure.

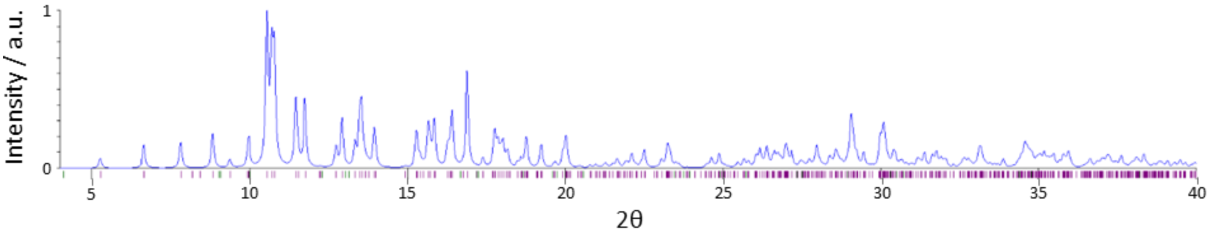

Figure S23. Theoretical powder X-ray diffraction pattern of **4-Dy** derived from crystallographic parameters.

Table S1. Unit cell values obtained from Pawley refinement results.

Complex	a	b	c	α	β	γ
**2-Dy**	20.65(2)	25.61(2)	22.05(2)	90	91.16(2)	90
**3-Dy**	19.11(2)	20.27(3)	32.54(4)	88.01(6)	76.13(4)	65.56(5)
**4-Dy**	10.57(2)	21.35(4)	26.60(5)	90	90.94(2)	90

5. Single Crystal X-ray Diffraction

Single crystal X-ray diffraction (SCXRD) data for compounds **1-Ln**, **2-Ln**, **2-Dy**, **3-Dy**, **4-Dy**, **5-Dy**, **6-Dy**, **7**, **8-Dy**, **10-13-Dy** and **14-Y** were collected using a dual wavelength Rigaku FR-X rotating anode diffractometer using CuKα (λ = 1.54146 Å) radiation, equipped with an AFC-11 4-circle goniometer, VariMAX[TM] microfocus optics, a Hypix-6000HE detector and an Oxford Cryosystems 800 plus nitrogen flow gas system, at a temperature of 100 K. Crystals of **9-Dy** were examined using an Oxford Diffraction Supernova diffractometer, furnished with a CCD area detector and a mirror-monochromated Mo Kα radiation (λ = 0.71073 Å) at a temperature of 175 K. Data were collected and reduced using CrysAlisPro v42.[6] Absorption correction was performed using empirical methods (SCALE3 ABSPACK) based upon symmetry-equivalent reflections combined with measurements at different azimuthal angles. The structures for were solved and refined against all F^2 values using Shelx-2018/3 implemented through Olex2 v1.5.[9,10] ORTEP-3,[11] POV-Ray[12] and Diamond[13] were employed for molecular graphics.

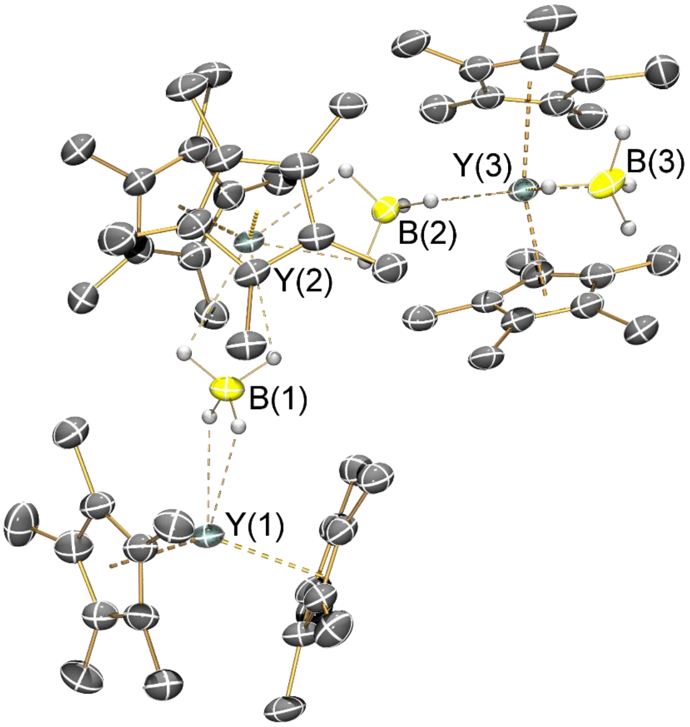

Figure S24. SCXRD structure of a portion of polymeric **1-Y** with selected atom labelling (Y: cyan, C: gray, B: yellow, H: white). Displacement ellipsoids set at 50% probability level, lattice solvent and hydrogen atoms are omitted for clarity, with the exception of those belonging to the BH_4^- groups. Selected mean distances and angles: Y···Cp*$_{centroid}$ 2.374(3) Å, Y···B 2.952(3) Å, Cp*$_{centroid}$···Y···Cp*$_{centroid}$ 133.02(11)°, B···Y···B 105.4(3)°.

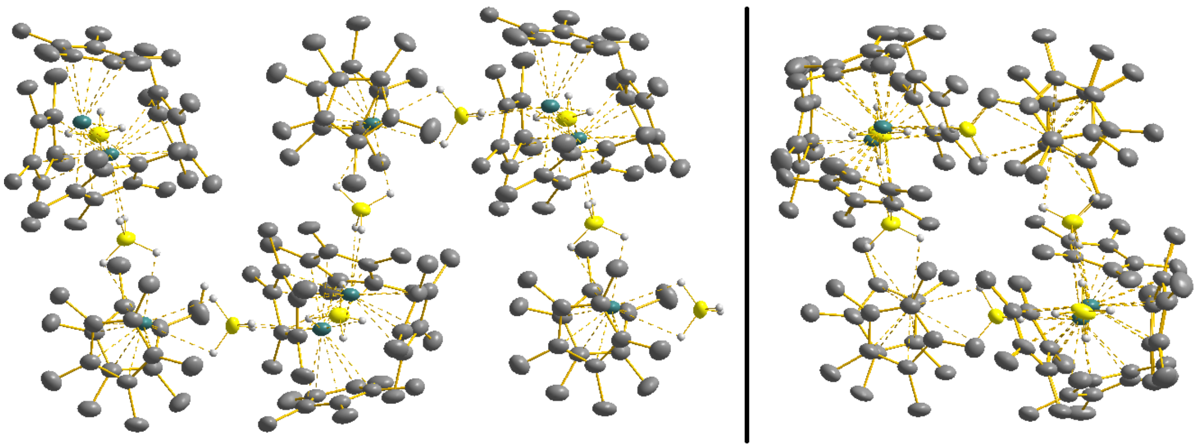

Figure S25. Depictions of the polymeric structure of **1-Y** with selected atom labelling (Y: cyan, C: gray, B: yellow, H: white). The lattice solvent and hydrogen atoms are omitted for clarity, with the exception of those belonging to the BH_4^- groups.

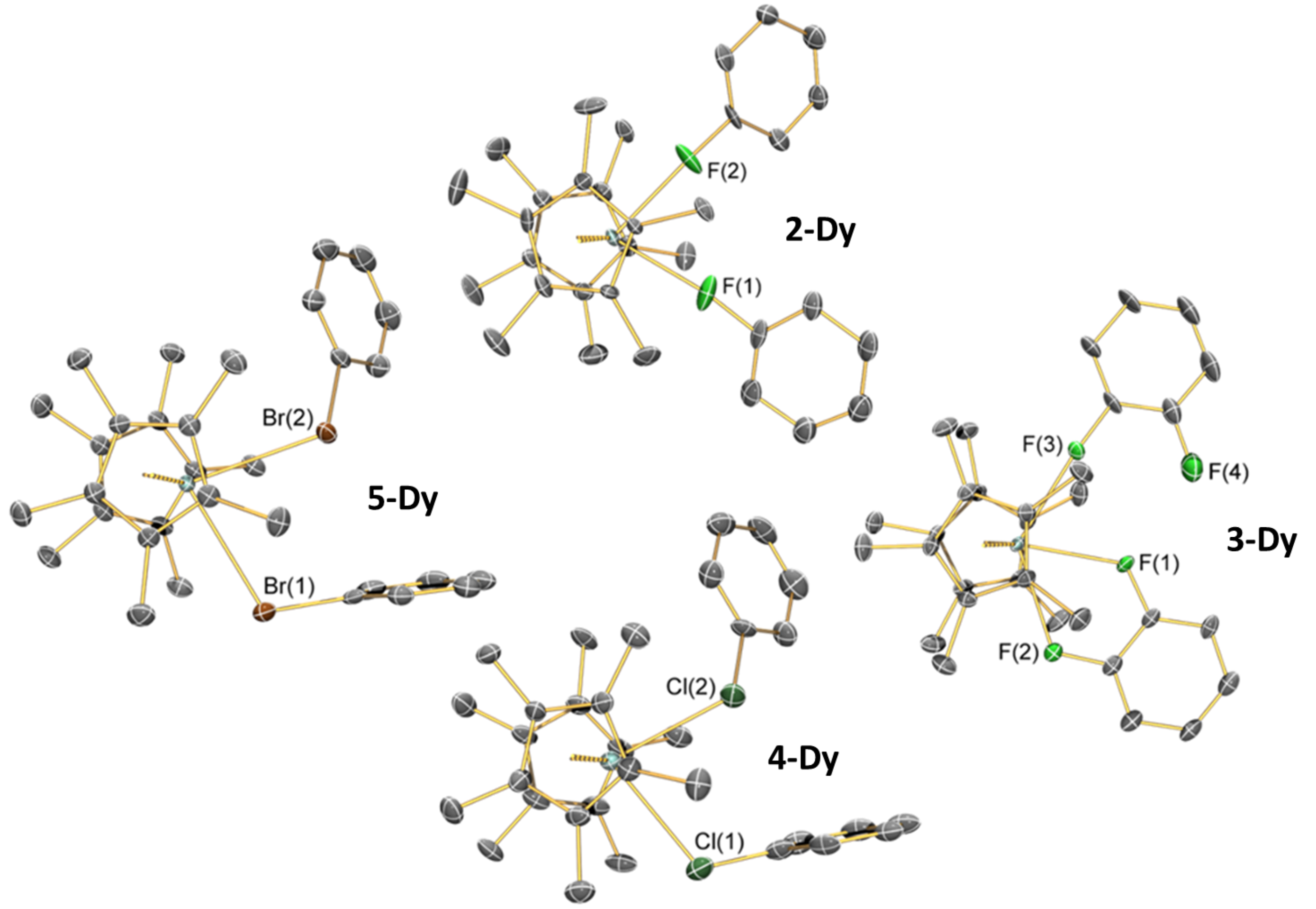

Figure S26. Top view of the SCXRD structures of **2-Dy**, **3-Dy**, **4-Dy** and **5-Dy** with selected atom labelling (Dy: cyan, C: gray, F: green, Cl: dark green, Br: brown). Displacement ellipsoids set at 30% probability levels. The $[Al\{OC(CF_3)_3\}_4]^-$ anions, lattice solvent and hydrogen atoms have been omitted for clarity.

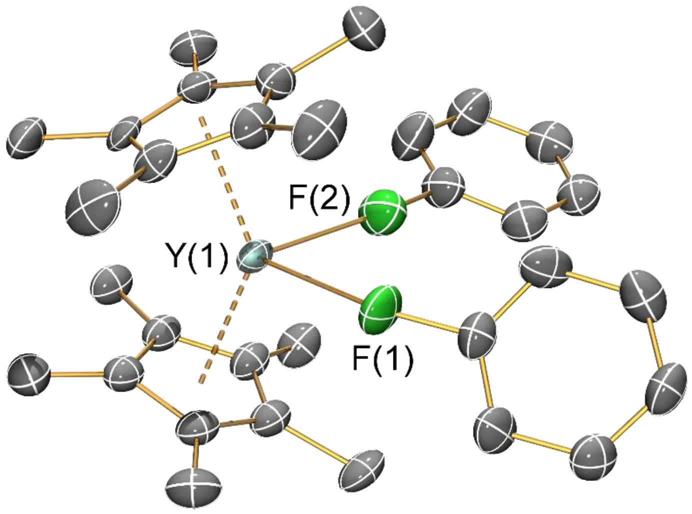

Figure S27. SCXRD structure of **2-Y** with selected atom labelling (Y: cyan, C: gray, F: green). Displacement ellipsoids set at 30% probability levels. The $[Al\{OC(CF_3)_3\}_4]^-$ anion and hydrogen atoms have been omitted for clarity. Selected bond lengths and angles: Y(1)–F(1) 2.386(7) Å, Y(1)–F(2) 2.381(5) Å, F(1)–C(21) 1.422(13) Å, F(2)–C(27) 1.405(10) Å, F(1)–Y(1)–F(2) 79.8(2)°, Y(1)–F(1)–C(21) 170.2(5)°, Y(1)–F(1)–C(27) 154.0(5)°. Selected mean bond lengths and angles: Y···$Cp^*_{centroid}$ 2.302(7) Å, $Cp^*_{centroid}$···Y···$Cp^*_{centroid}$ 139.9(6)°.

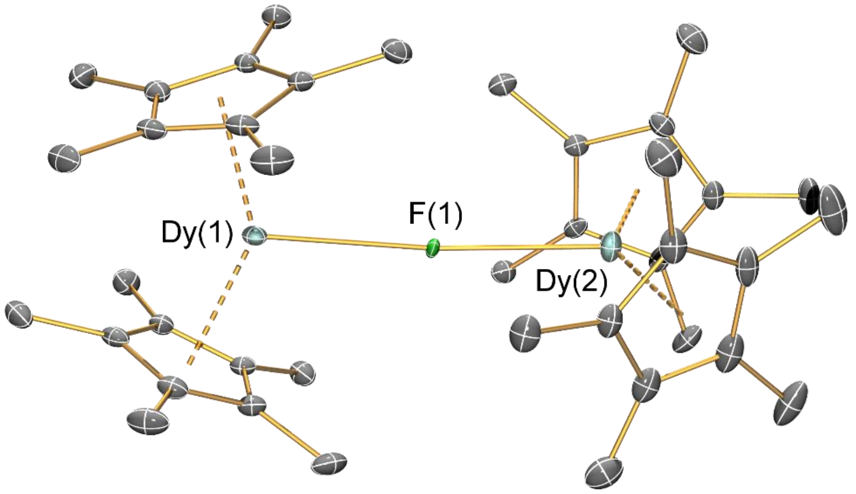

Figure S28. SCXRD structure of **6-Dy** with selected atom labelling (Dy: cyan, C: gray, F: green). Displacement ellipsoids set at 30% probability levels. The $[Al\{OC(CF_3)_3\}_4]^-$ anion and hydrogen atoms have been omitted for clarity. Selected bond lengths and angles: Dy(1)···Dy(2) 5.4426(6) Å, Dy(1)–F(1) 2.751(3) Å, Dy(2)–F(1) 2.715(3) Å, Dy(1)–F(1)–Dy(2) 169.44(10)°. Selected mean bond lengths and angles: Dy···Cp*$_{centroid}$ 2.300(2) Å, Cp*$_{centroid}$···Dy···Cp*$_{centroid}$ 141.75(13)°.

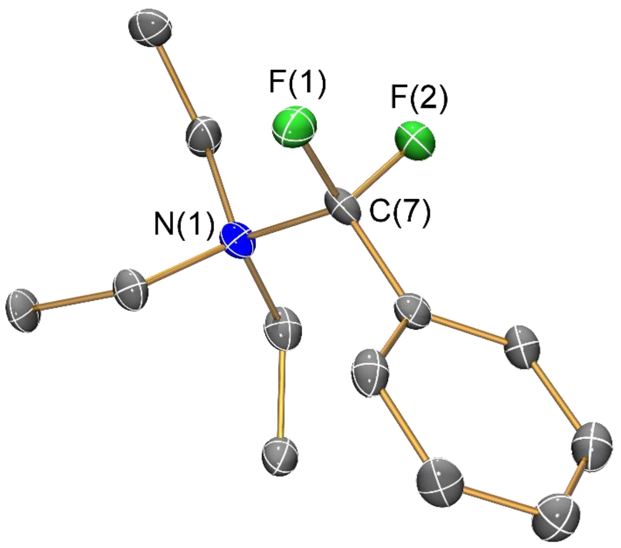

Figure S29. SCXRD structure of **7** with selected atom labelling (C: gray, N: blue, F: green). Displacement ellipsoids set at 30% probability levels. The $[Al\{OC(CF_3)_3\}_4]^-$ anion and hydrogen atoms have been omitted for clarity. Selected bond lengths and angles: N(1)–C(7) 1.549(13) Å, C(7)–F(1) 1.35(2) Å, C(7)–F(2) 1.344(13) Å, N(1)–C(7)–C(1) 117.2(8)°.

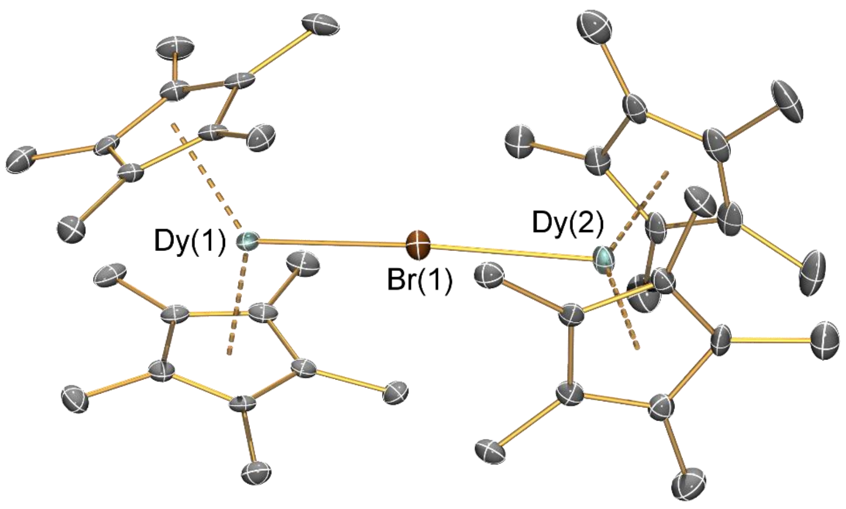

Figure S30. SCXRD structure of **8-Dy** with selected atom labelling (Dy: cyan, C: gray, Br: brown). Displacement ellipsoids set at 30% probability levels. The $[Al\{OC(CF_3)_3\}_4]^-$ anion and hydrogen atoms have been omitted for clarity. Selected bond lengths and angles: Dy(1)···Dy(2) 5.5621(7) Å, Dy(1)–Br(1)–Dy(2) 165.16(3)°. Selected mean bond lengths and angles: Dy···Cp*$_{centroid}$ 2.298(2) Å, Dy–Br 2.805(2) Å, Cp*$_{centroid}$···Dy···Cp*$_{centroid}$ 141.8(2)°.

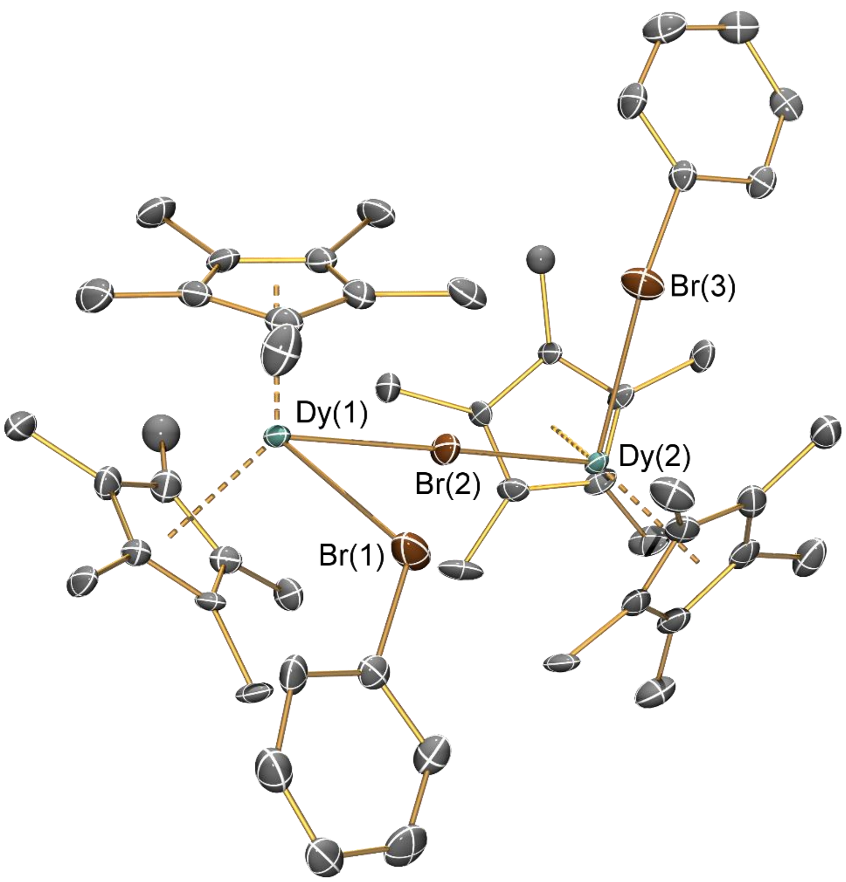

Figure S31. SCXRD structure of **9-Dy** with selected atom labelling (Dy: cyan, C: gray, Br: brown). Displacement ellipsoids set at 30% probability levels. The $[Al\{OC(CF_3)_3\}_4]^-$ anion and hydrogen atoms have been omitted for clarity. Selected bond lengths and angles: Dy(1)···Dy(2) 5.6731(8) Å, Dy(1)–Br(1) 3.178(2) Å, Dy(2)–Br(3) 3.189(2) Å, Br(1)–C(21) 1.924(13) Å, Br(3)–C(47) 1.923(13) Å, Dy(1)–Br(2)–Dy(2) 176.43(8)°. Selected mean bond lengths and angles: Dy···$Cp^*_{centroid}$ 2.337(4) Å, Dy–Br(2) 2.838(2) Å, $Cp^*_{centroid}$···Dy···$Cp^*_{centroid}$ 137.75(4)°, Br–Dy–Br(2) 77.77(7)°.

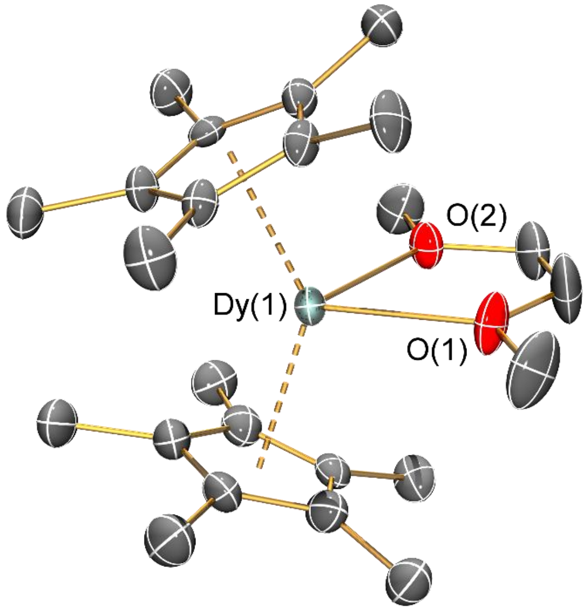

Figure S32. SCXRD structure of **10-Dy** with selected atom labelling (Dy: cyan, C: gray, O: red). Displacement ellipsoids set at 30% probability levels. The $[Al\{OC(CF_3)_3\}_4]^-$ anion and hydrogen atoms have been omitted for clarity. Selected bond lengths: Dy–O(1) 2.384(6) Å, Dy–O(2) 2.284(5) Å. Selected mean bond lengths and angles: Dy···$Cp^*_{centroid}$ 2.344(5) Å, $Cp^*_{centroid}$···Dy···$Cp^*_{centroid}$ 138.6(4)°.

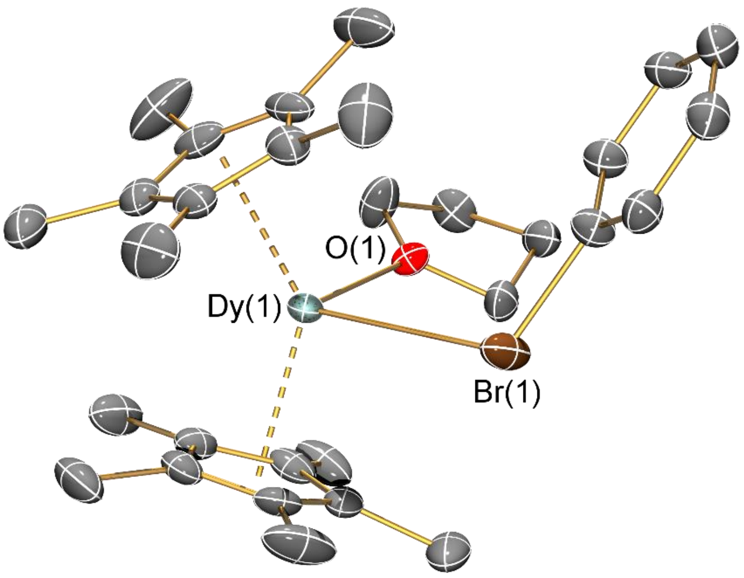

Figure S33. SCXRD structure of **11-Dy** with selected atom labelling (Dy: cyan, C: gray, O: red, Br: brown). Displacement ellipsoids set at 30% probability levels. The $[Al\{OC(CF_3)_3\}_4]^-$ anion and hydrogen atoms have been omitted for clarity. Selected bond lengths and angles: Dy–O(1) 2.399(5) Å, Dy–Br(1) 3.0015(12) Å, $Cp^*_{centroid}\cdots Dy(1)\cdots Cp^*_{centroid}$ 135.6(2)°. Selected mean bond lengths and angles: $Dy\cdots Cp^*_{centroid}$ 2.352(6) Å, Br(1)–C 1.918(12) Å, Dy(1)–Br(1)–C 117.25(10)°.

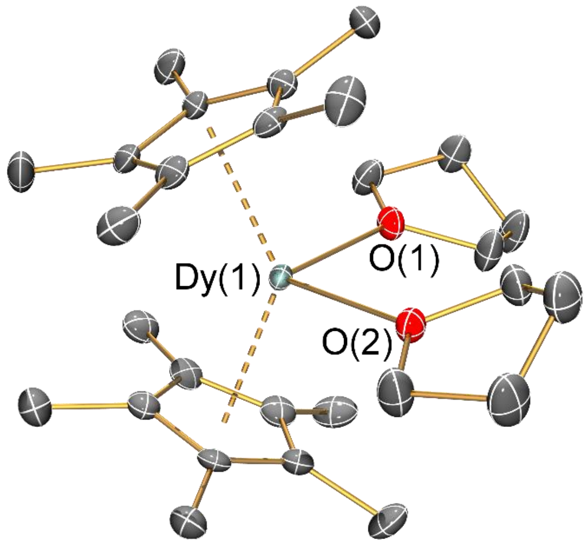

Figure S34. SCXRD structure of **12-Dy** with selected atom labelling (Dy: cyan, C: gray, O: red). Displacement ellipsoids set at 30% probability levels. The $[Al\{OC(CF_3)_3\}_4]^-$ anion and hydrogen atoms have been omitted for clarity. Selected bond lengths and angles: Dy–O(1) 2.408(5) Å, Dy–O(2) 2.408(6) Å, $Cp^*_{centroid}\cdots Dy\cdots Cp^*_{centroid}$ 136.47(2)°, O(1)–Dy(1)–O(2) 94.2(2)°. Selected mean bond length: $Dy\cdots Cp^*_{centroid}$ 2.360(4) Å.

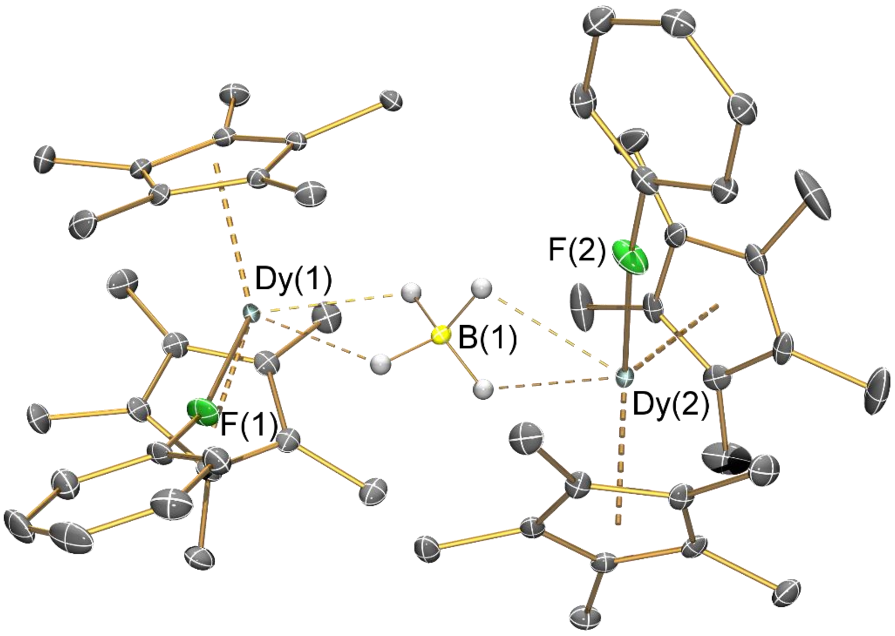

Figure S35. SCXRD structure of **13-Dy** with selected atom labelling (Dy: cyan, C: gray, F: green). Displacement ellipsoids set at 30% probability levels. The $[Al\{OC(CF_3)_3\}_4]^-$ anion and hydrogen atoms have been omitted for clarity. Selected bond lengths and angles: Dy(1)···Dy(2) 5.5491(5) Å, Dy(1)–B(1) 2.768(4) Å, Dy(2)–B(1) 2.799(3) Å, Dy(1)–F(1) 2.459(2) Å, Dy(2)–F(2) 2.463(2) Å, F(1)–C(21) 1.392(4) Å, F(2)–C(47) 1.393(4) Å, Dy(1)–B(1)–Dy(2) 170.74(12)°. Selected mean bond lengths and angles: Dy···$Cp^*_{centroid}$ 2.3404(4) Å, $Cp^*_{centroid}$···Dy···$Cp^*_{centroid}$ 138.60(6)°.

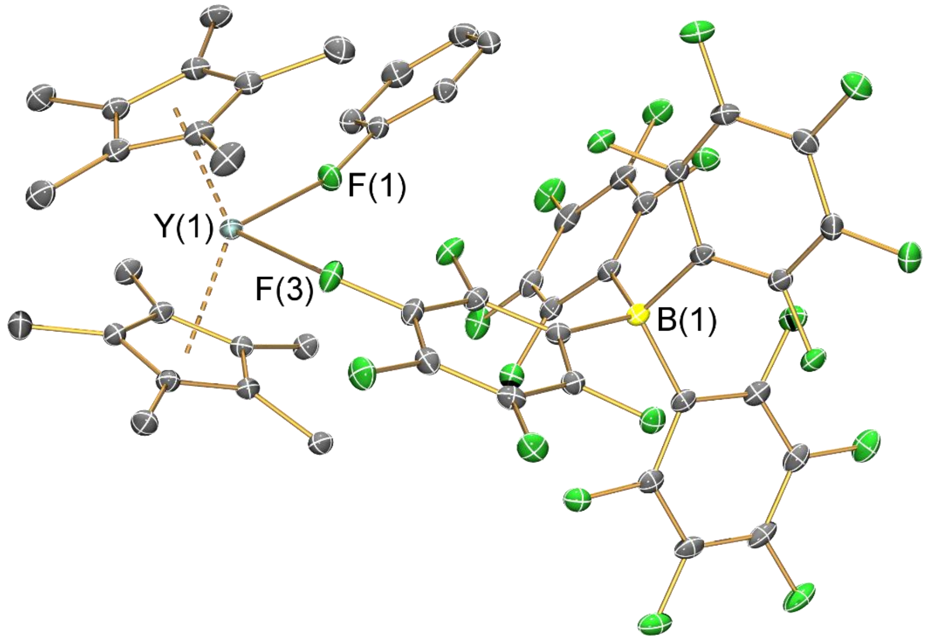

Figure S36. SCXRD structure of **14-Y** with selected atom labelling (Dy: cyan, C: gray, B: yellow, F: green). Displacement ellipsoids set at 30% probability levels. Hydrogen atoms have been omitted for clarity. Selected bond lengths and angles: Y(1)–F(1) 2.3483(12) Å, Y(1)–F(3) 2.4228(12) Å, Y(1)–F(1)–C_{ipso} 164.47(6)°, Y(1)–F(3)–C_{ipso} 145.21(5)°, F(1)–Y(1)–F(3) 97.93(4)°, $Cp*_{centroid}$···Y···$Cp*_{centroid}$ 139.90(3)°.Selected mean bond length: Y···$Cp*_{centroid}$ 2.3097(13) Å.

Table S2. Crystallographic data for **1-Y** and **1-Dy**.

	**1-Y**	**1-Dy**
Formula	$C_{141}H_{246}B_6Y_6$	$C_{67}H_{116}B_3Dy_3$
molecular mass, g mol^{-1}	2539.68	1441.52
cryst size, mm	0.107 × 0.092 × 0.039	0.106 × 0.091 × 0.061
cryst syst	monoclinic	monoclinic
space group	$P2_1/n$	$P2_1/n$
collection temperature, K	100(2)	100(2)
a, Å	17.8309(7)	17.8227(3)
b, Å	15.8094(8)	15.7486(6)
c, Å	24.0617(10)	24.0672(6)
α, °	90	90
β, °	91.068(4)	91.333(2)
γ, °	90	90
V, Å^3	6781.7(5)	6753.4(3)
Z	2	4
ρ_{calcd}, g cm^{-3}	1.244	1.418
μ, mm^{-1}	3.624	17.729
no. of reflections made	48948	33570
no. of unique reflns, R_{int}	13856, 0.1184	13375, 0.0783
no. of reflns with $F^2 > 2\sigma(F^2)$	8788	8967
transmn coeff range	0.899–1.000	0.885–1.000
R, R_w[a] ($F^2 > 2\sigma(F^2)$)	0.0630, 0.1541	0.0526, 0.1211
R, R_w[a] (all data)	0.1082, 0.1823	0.0911, 0.1371
S[a]	0.991	0.985
parameters, restraints	851, 634	917, 1315
max., min. diff map, e Å^{-3}	1.220, –1.457	1.044, –1.126

[a] Conventional $R = \Sigma||F_o| - |F_c||/\Sigma|F_o|$; $R_w = [\Sigma w(F_o^2 - F_c^2)^2/\Sigma w(F_o^2)^2]^{1/2}$; $S = [\Sigma w(F_o^2 - F_c^2)^2/\text{no. data} - \text{no. params})]^{1/2}$ for all data.

Table S3. Crystallographic data for **2-Y** and **2-Dy**.

	**2-Y**	**2-Dy**
Formula	$C_{48}H_{40}AlF_{38}O_4Y$	$C_{48}H_{40}AlDyF_{38}O_4$
molecular mass, g mol^{-1}	1518.69	1592.28
cryst size, mm	0.088 × 0.060 × 0.037	0.289 × 0.245 × 0.174
cryst syst	monoclinic	monoclinic
space group	$P2_1/n$	$P2_1$
collection temperature, K	100(2)	100(2)
a, Å	20.6700(4)	20.62450(10)
b, Å	25.4690(5)	25.4804(2)
c, Å	22.0609(5)	22.01900(10)
α, °	90	90
β, °	90.820(2)	90.9040(10)
γ, °	90	90
V, Å^3	11612.6(4)	11570.00(12)
Z	8	8
ρ_{calcd}, g cm^{-3}	1.737	1.828
μ, mm^{-1}	3.100	8.602
no. of reflections made	77022	150431
no. of unique reflns, R_{int}	18588, 0.0987	42103, 0.0740
no. of reflns with $F^2 > 2\sigma(F^2)$	12029	37739
transmn coeff range	0.962–1.000	0.226–1.000
R, R_w[a] ($F^2 > 2\sigma(F^2)$)	0.1160, 0.2974	0.0713, 0.1948
R, R_w[a] (all data)	0.1620, 0.3318	0.0769, 0.2000
S[a]	0.994	1.127
parameters, restraints	2190, 8055	3868, 16693
max., min. diff map, e Å^{-3}	0.928, –2.017	1.306, –1.855

[a] Conventional $R = \Sigma||F_o| - |F_c||/\Sigma|F_o|$; $R_w = [\Sigma w(F_o^2 - F_c^2)^2/\Sigma w(F_o^2)^2]^{1/2}$; $S = [\Sigma w(F_o^2 - F_c^2)^2/\text{no. data} - \text{no. params})]^{1/2}$ for all data.

Table S4. Crystallographic data for **3-Dy** and **4-Dy**.

	**3-Dy**	**4-Dy**
Formula	$C_{48}H_{38}AlDyF_{40}O_4$	$C_{102}H_{85}Al_2Cl_5Dy_2F_{72}O_8$
molecular mass, g mol^{-1}	1628.26	3362.90
cryst size, mm	0.348 × 0.237 × 0.195	0.348 × 0.237 × 0.195
cryst syst	Triclinic	monoclinic
space group	$P\bar{1}$	$P2_1/c$
collection temperature, K	100(2)	100(2)
a, Å	19.4825(3)	10.4788(2)
b, Å	20.2282(4)	21.5737(4)
c, Å	33.3534(5)	26.5259(4)
α, °	87.8541(14)	90
β, °	75.9026(14)	90.459(2)
γ, °	65.5559(17)	90
V, $Å^3$	11576.0(4)	5996.42(18)
Z	8	2
ρ_{calcd}, g cm^{-3}	1.869	1.863
μ, mm^{-1}	8.666	9.291
no. of reflections made	162208	17264
no. of unique reflns, R_{int}	44437, 0.0606	17264
no. of reflns with $F^2 > 2\sigma(F^2)$	32544	15711
transmn coeff range	0.597-1.000	0.597–1.000
R, R_w^a ($F^2 > 2\sigma(F^2)$)	0.0689, 0.1945	0.0535, 0.1451
R, R_w^a (all data)	0.0874, 0.2102	0.0576, 0.1473
S^a	1.069	1.027
parameters, restraints	4439, 19204	891, 84
max., min. diff map, e $Å^{-3}$	1.895, -2.920	1.396, –1.946

[a] Conventional $R = \Sigma||F_o| - |F_c||/\Sigma|F_o|$; $R_w = [\Sigma w(F_o^2 - F_c^2)^2/\Sigma w(F_o^2)^2]^{1/2}$; $S = [\Sigma w(F_o^2 - F_c^2)^2/\text{no. data} - \text{no. params})]^{1/2}$ for all data.

Table S5. Crystallographic data for **5-Dy** and **6-Dy**.

	**5-Dy**	**6-Dy**
Formula	$C_{48}H_{40}AlBr_2DyF_{36}O_4$	$C_{56}H_{60}AlDy_2F_{37}O_4$
molecular mass, g mol^{-1}	1714.10	1852.02
cryst size, mm	0.330 × 0.177 × 0.150	0.229 × 0.155 × 0.115
cryst syst	monoclinic	monoclinic
space group	$P2_1/c$	$P2_1/c$
collection temperature, K	100(2)	100(2)
a, Å	10.4584(2)	17.0826(2)
b, Å	21.6145(4)	19.8233(3)
c, Å	26.7676(6)	21.0768(3)
α, °	90	90
β, °	91.162(2)	104.3610(13)
γ, °	90	90
V, $Å^3$	6049.7(2)	6914.30(17)
Z	4	4
ρ_{calcd}, g cm^{-3}	1.882	1.779
μ, mm^{-1}	2.723	12.862
no. of reflections made	44886	39908
no. of unique reflns, R_{int}	14181, 0.0293	13938, 0.0302
no. of reflns with $F^2 > 2\sigma(F^2)$	11679	12327
transmn coeff range	0.703–1.000	0.757–1.000
R, R_w[a] ($F^2 > 2\sigma(F^2)$)	0.0271, 0.0600	0.0536, 0.1500
R, R_w[a] (all data)	0.0386, 0.0630	0.0591, 0.1544
S[a]	0.977	1.022
parameters, restraints	948, 1293	1393, 3953
max., min. diff map, e $Å^{-3}$	0.924, –0.546	2.032, –1.582

[a] Conventional $R = \Sigma||F_o| - |F_c||/\Sigma|F_o|$; $R_w = [\Sigma w(F_o^2 - F_c^2)^2/\Sigma w(F_o^2)^2]^{1/2}$; $S = [\Sigma w(F_o^2 - F_c^2)^2/\text{no. data} - \text{no. params})]^{1/2}$ for all data.

Table S6. Crystallographic data for **7** and **8-Dy**.

	**7**	**8-Dy**
Formula	$C_{29}H_{20}AlF_{38}NO_4$	$C_{56}H_{60}AlBrDy_2F_{36}O_4$
molecular mass, g mol^{-1}	1195.44	1912.93
cryst size, mm	0.171 × 0.098 × 0.082	0.091 × 0.074 × 0.047
cryst syst	monoclinic	monoclinic
space group	$P2_1/c$	$P2_1/c$
collection temperature, K	100(2)	100(2)
a, Å	16.7883(11)	17.0861(5)
b, Å	13.4028(6)	19.7751(5)
c, Å	19.8723(12)	21.1901(6)
α, °	90	90
β, °	114.972(7)	104.847(3)
γ, °	90	90
V, Å^3	4053.5(5)	6920.7(4)
Z	4	4
ρ_{calcd}, g cm^{-3}	1.959	1.836
μ, mm^{-1}	2.482	13.504
no. of reflections made	13085	38317
no. of unique reflns, R_{int}	13085	13315, 0.0577
no. of reflns with $F^2 > 2\sigma(F^2)$	9404	10981
transmn coeff range	0.875–1.000	0.863–1.000
R, R_w[a] ($F^2 > 2\sigma(F^2)$)	0.0607, 0.1699	0.0515, 0.1441
R, R_w[a] (all data)	0.0778, 0.1824	0.0629, 0.1531
S[a]	1.032	1.053
parameters, restraints	662, 0	1426, 3685
max., min. diff map, e Å^{-3}	0.395, –0.506	1.317, –1.648

[a] Conventional $R = \Sigma||F_o| - |F_c||/\Sigma|F_o|$; $R_w = [\Sigma w(F_o^2 - F_c^2)^2/\Sigma w(F_o^2)^2]^{1/2}$; $S = [\Sigma w(F_o^2 - F_c^2)^2$/no. data – no. params)$]^{1/2}$ for all data.

Table S7. Crystallographic data for **9-Dy** and **10-Dy**.

	**9-Dy**	**10-Dy**
Formula	$C_{68}H_{70}AlBr_3Dy_2F_{36}O_4$	$C_{40}H_{40}AlDyF_{36}O_6$
molecular mass, g mol^{-1}	2226.95	1490.20
cryst size, mm	0.563 × 0.414 × 0.235	0.486 × 0.109 × 0.101
cryst syst	Monoclinic	triclinic
space group	$P2_1/c$	$P\bar{1}$
collection temperature, K	175(2)	100(2)
a, Å	10.4638(5)	10.4377(4)
b, Å	25.7418(9)	15.3020(4)
c, Å	30.0529(13)	16.7339(5)
α, °	90	86.525(2)
β, °	90.360(5)	85.610(3)
γ, °	90	88.654(2)
V, Å^3	8094.8(6)	2659.48(15)
Z	4	2
ρ_{calcd}, g cm^{-3}	1.827	1.861
μ, mm^{-1}	3.451	9.272
no. of reflections made	19866	28030
no. of unique reflns, R_{int}	19866	10602, 0.0482
no. of reflns with $F^2 > 2\sigma(F^2)$	10710	8983
transmn coeff range	0.331–1.000	0.647–1.000
R, R_w[a] ($F^2 > 2\sigma(F^2)$)	0.0644, 0.1419	0.0745, 0.2046
R, R_w[a] (all data)	0.1172, 0.1524	0.0842, 0.2156
S[a]	0.884	1.007
parameters, restraints	1407, 4186	1466, 5184
max., min. diff map, e Å^{-3}	3.743, –3.171	1.687, –1.707

[a] Conventional $R = \Sigma||F_o| - |F_c||/\Sigma|F_o|$; $R_w = [\Sigma w(F_o^2 - F_c^2)^2/\Sigma w(F_o^2)^2]^{1/2}$; $S = [\Sigma w(F_o^2 - F_c^2)^2$/no. data – no. params)]$^{1/2}$ for all data.

Table S8. Crystallographic data for **11-Dy** and **12-Dy**.

	**11-Dy**	**12-Dy**
Formula	$C_{46}H_{43}AlBrDyF_{36}O_5$	$C_{44}H_{46}AlDyF_{36}O_6$
molecular mass, g mol^{-1}	1629.19	1544.29
cryst size, mm	0.114 × 0.101 × 0.067	0.165 × 0.082 × 0.057
cryst syst	monoclinic	monoclinic
space group	$P2_1/c$	*P*c
collection temperature, K	100(2)	100(2)
a, Å	16.8415(8)	11.8969(2)
b, Å	19.1894(6)	29.5120(5)
c, Å	18.9552(9)	15.9063(3)
α, °	90	90
β, °	111.492(5)	89.9852(15)
γ, °	90	90
V, Å^3	5700.0(5)	5584.71(17)
Z	4	4
ρ_{calcd}, g cm^{-3}	1.898	1.837
μ, mm^{-1}	2.190	1.519
no. of reflections made	30089	73944
no. of unique reflns, R_{int}	11447, 0.0552	25642, 0.0349
no. of reflns with $F^2 > 2\sigma(F^2)$	7457	22772
transmn coeff range	0.917–1.000	0.852–1.000
R, R_w[a] ($F^2 > 2\sigma(F^2)$)	0.0617, 0.1505	0.0298, 0.0618
R, R_w[a] (all data)	0.1079, 0.1726	0.0373, 0.0640
S[a]	0.933	1.116
parameters, restraints	1349, 4238	1966, 6278
max., min. diff map, e Å^{-3}	1.621, –1.010	0.771, –0.613

[a] Conventional $R = \Sigma||F_o| - |F_c||/\Sigma|F_o|$; $R_w = [\Sigma w(F_o^2 - F_c^2)^2/\Sigma w(F_o^2)^2]^{1/2}$; $S = [\Sigma w(F_o^2 - F_c^2)^2/\text{no. data} - \text{no. params})]^{1/2}$ for all data.

Table S9. Crystallographic data for **13-Dy** and **14-Y**.

	**13-Dy**	**14-Y**
Formula	$C_{68}H_{74}AlBDy_2F_{38}O_4$	$C_{50}H_{35}BF_{21}Y$
molecular mass, g mol^{-1}	2040.06	1134.50
cryst size, mm	0.461 × 0.163 × 0.131	0.160 × 0.115 × 0.108
cryst syst	triclinic	triclinic
space group	$P\bar{1}$	$P\bar{1}$
collection temperature, K	100(2)	100(2)
a, Å	14.3908(4)	9.94820(10)
b, Å	16.1076(6)	13.70810(10)
c, Å	17.8687(5)	16.94630(10)
α, °	71.100(3)	91.3800(10)
β, °	88.212(2)	94.3990(10)
γ, °	79.497(3)	93.5520(10)
V, Å^3	3851.3(2)	2298.78(3)
Z	2	2
ρ_{calcd}, g cm^{-3}	1.759	1.639
μ, mm^{-1}	2.075	2.899
no. of reflections made	54933	26352
no. of unique reflns, R_{int}	18978, 0.0335	9205, 0.0211
no. of reflns with $F^2 > 2\sigma(F^2)$	15119	8854
transmn coeff range	0.385–1.000	0.861–1.000
R, R_w[a] ($F^2 > 2\sigma(F^2)$)	0.0318, 0.0697	0.0305, 0.0757
R, R_w[a] (all data)	0.0473, 0.0741	0.0314, 0.0763
S[a]	1.018	1.036
parameters, restraints	1408, 3108	668, 0
max., min. diff map, e Å^{-3}	1.388, –0.661	0.597, –0.975

[a] Conventional $R = \Sigma||F_o| - |F_c||/\Sigma|F_o|$; $R_w = [\Sigma w(F_o^2 - F_c^2)^2/\Sigma w(F_o^2)^2]^{1/2}$; $S = [\Sigma w(F_o^2 - F_c^2)^2/\text{no. data} - \text{no. params})]^{1/2}$ for all data.

6. Infrared Spectroscopy

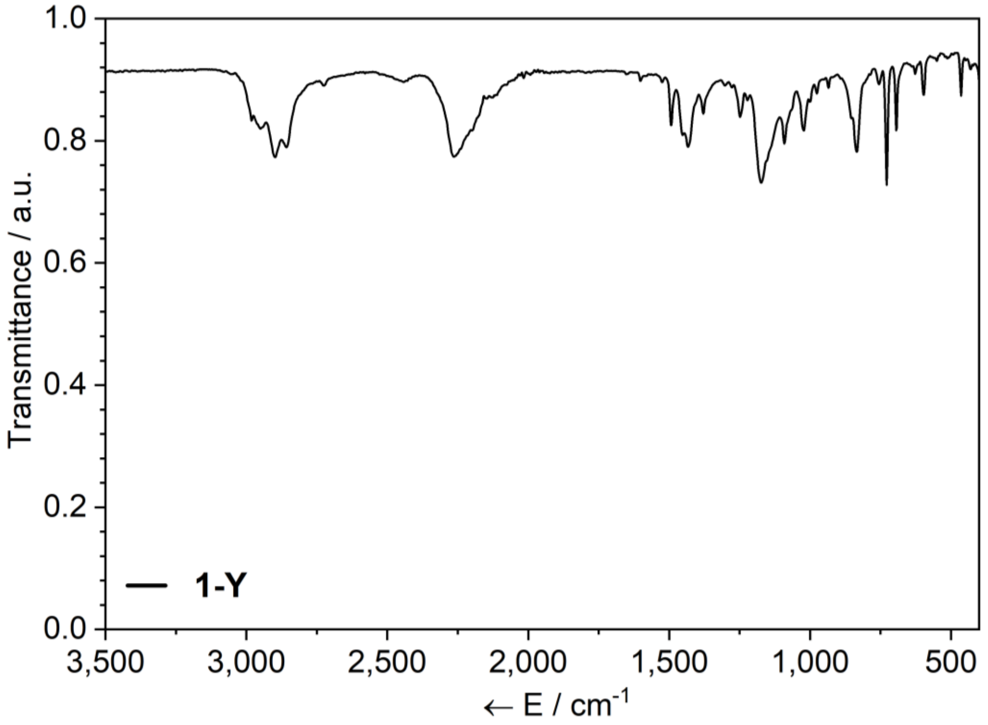

Figure S37. ATR-IR spectrum of **1-Y**, recorded as a microcrystalline powder.

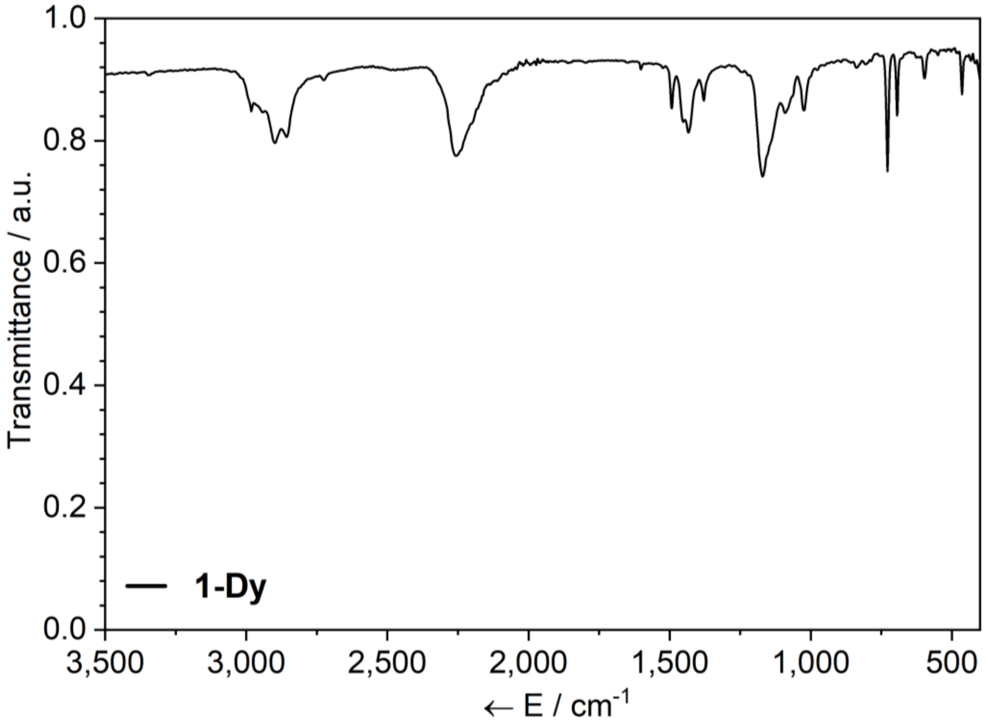

Figure S38. ATR-IR spectrum of **1-Dy**, recorded as a microcrystalline powder.

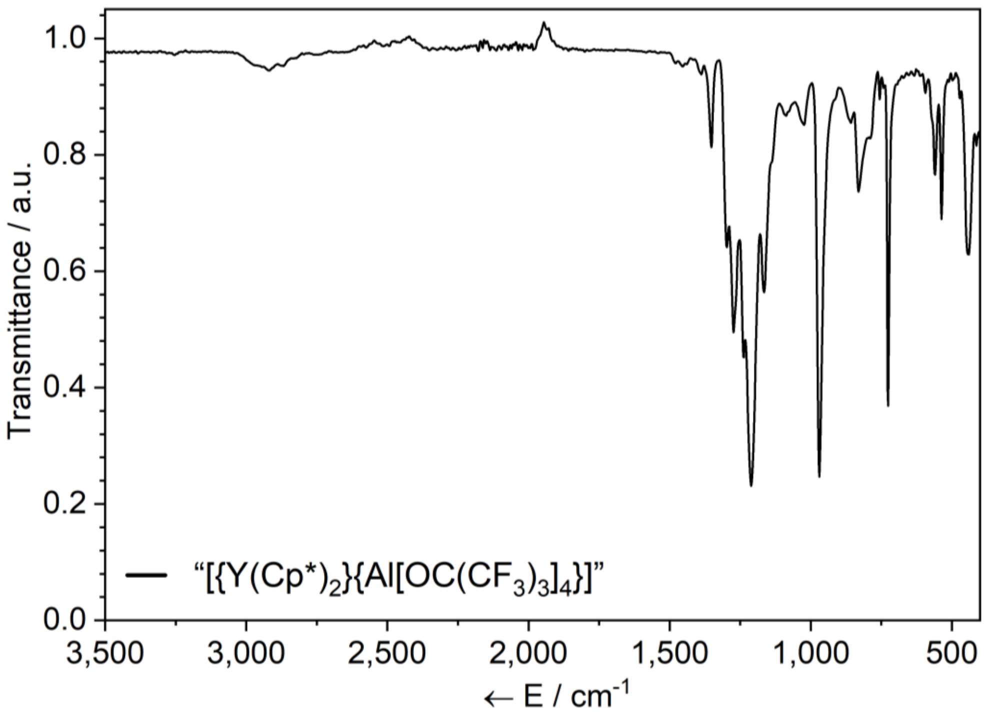

Figure S39. ATR-IR spectrum of "[{Y(Cp*)$_2$}{Al[OC(CF$_3$)$_3$]$_4$}]", recorded as an amorphous powder.

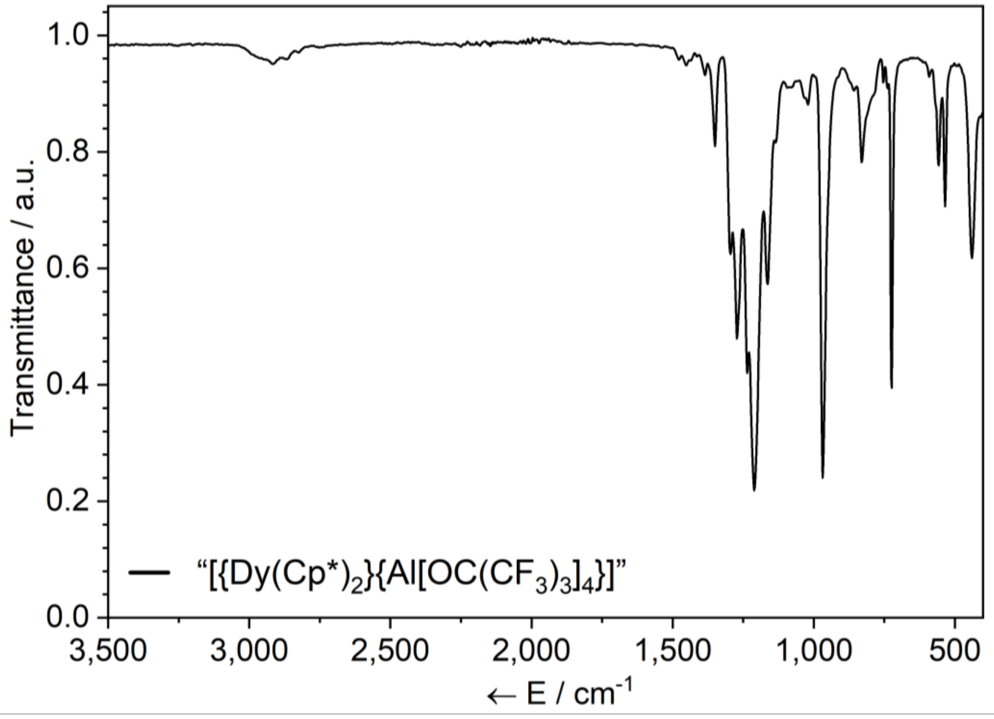

Figure S40. ATR-IR spectrum of "[{Dy(Cp*)$_2$}{Al[OC(CF$_3$)$_3$]$_4$}]", recorded as an amorphous powder.

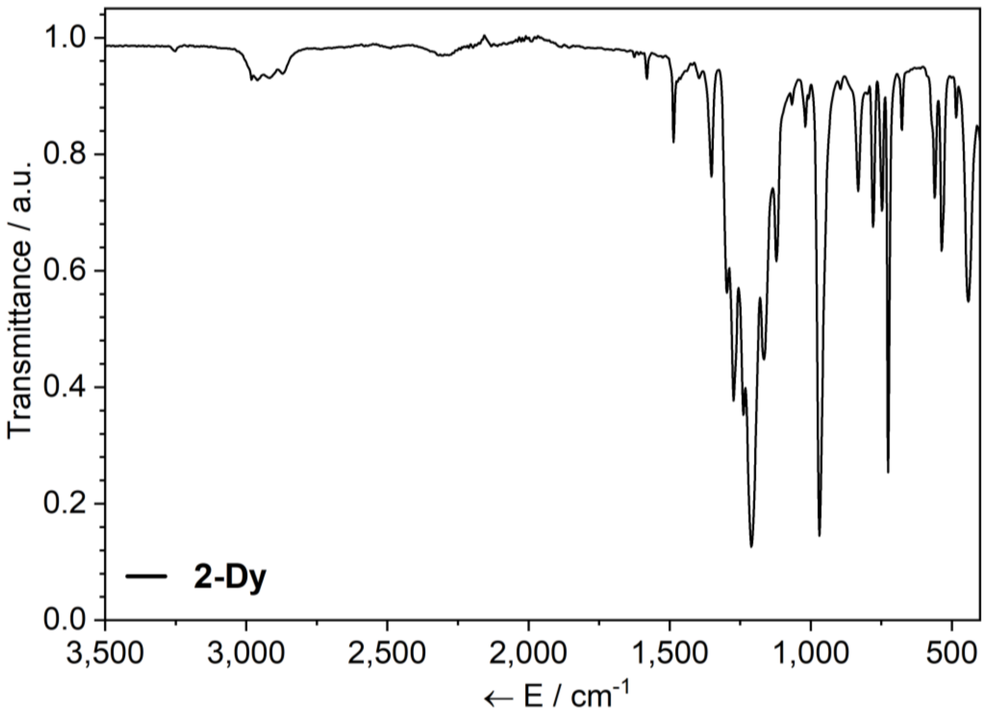

Figure S41. ATR-IR spectrum of **2-Dy**, recorded as a microcrystalline powder.

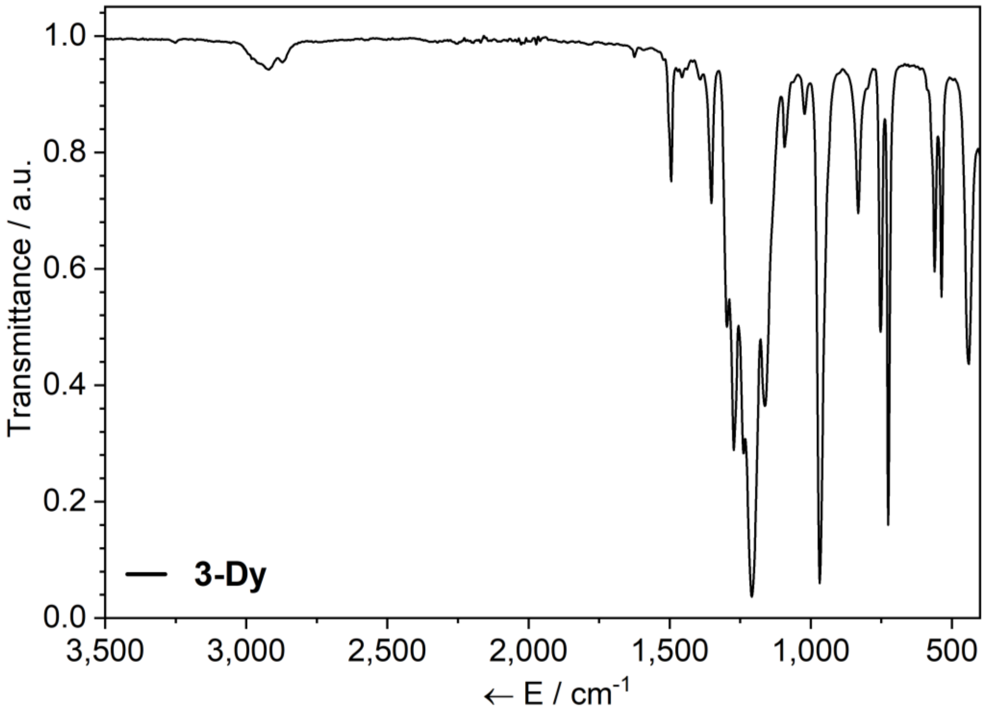

Figure S42. ATR-IR spectrum of **3-Dy**, recorded as a microcrystalline powder.

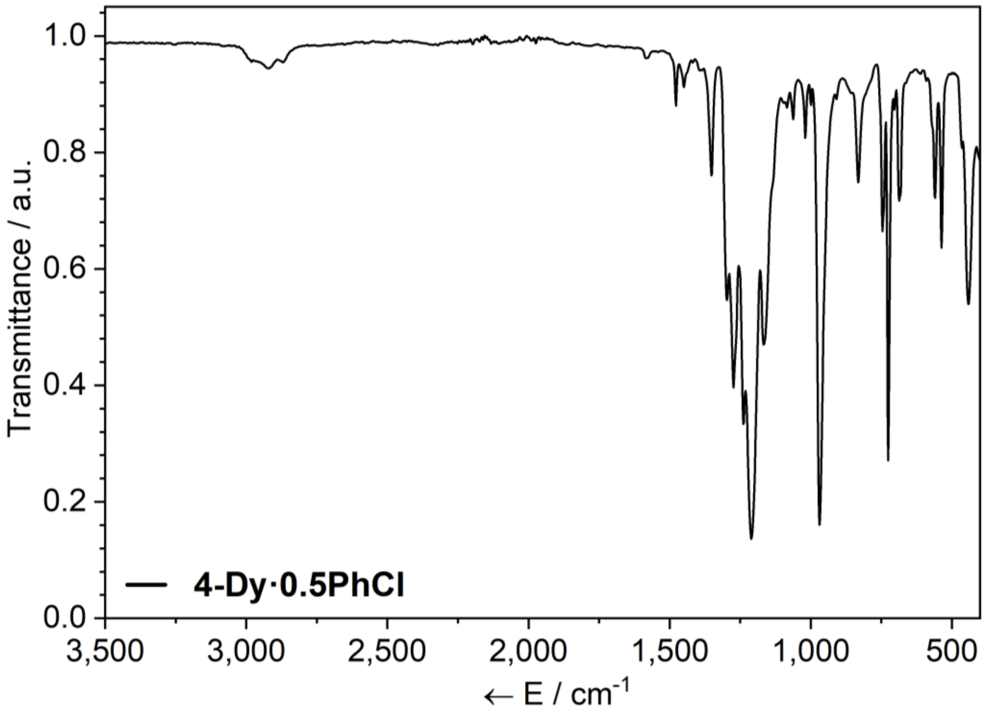

Figure S43. ATR-IR spectrum of **4-Dy**, recorded as a microcrystalline powder.

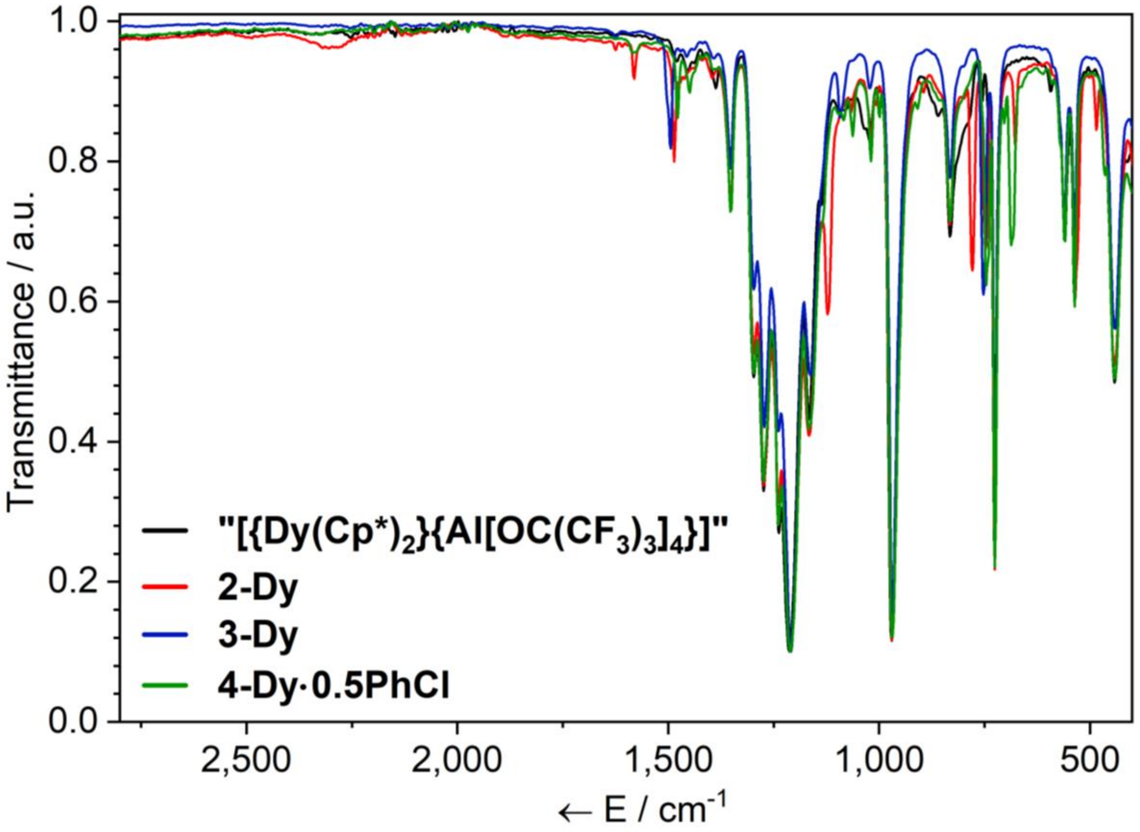

Figure S44. Combined normalized ATR-IR spectra of “[{Dy(Cp*)$_2$}{Al[OC(CF$_3$)$_3$]$_4$}]” and **2-Dy**, **3-Dy** and **4-Dy**, recorded as microcrystalline powders.

Crystalline samples of **2-Dy**, **3-Dy** and **4-Dy** were finely ground into powders and separately heated at 60 °C for 30 mins under vacuum, to determine if desolvation could be promoted at elevated temperatures. Analysis by ATR-IR spectroscopy showed that the C–X stretches decreased in intensity, but these changes are not conclusive as other absorptions are also affected, indicating that some degradation of these samples had occurred.

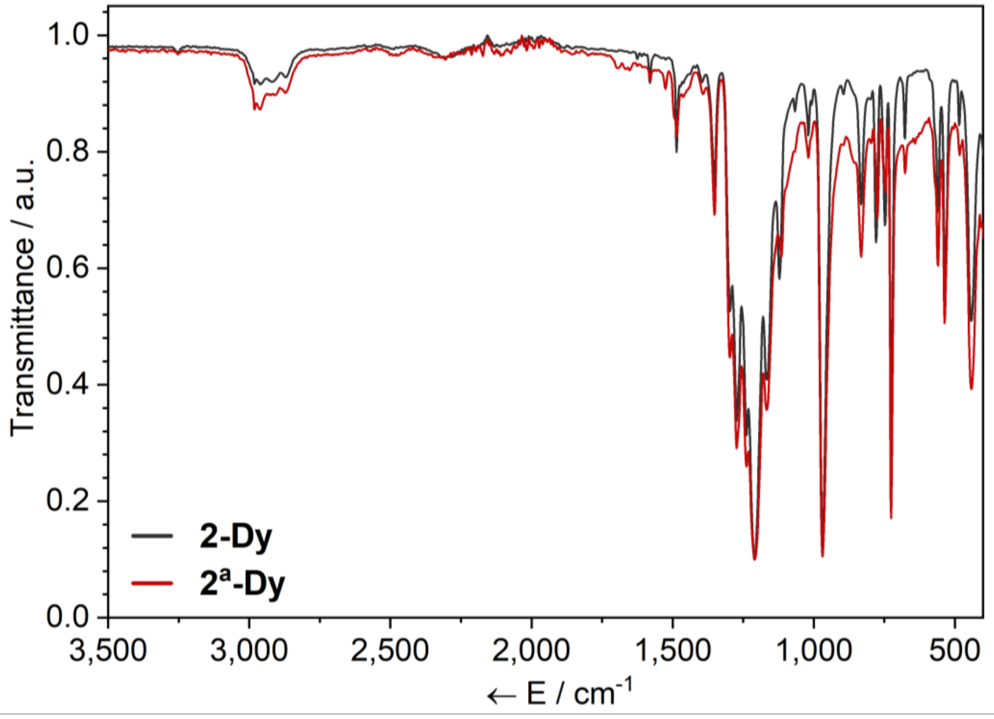

Figure S45. Normalized ATR-IR spectrum of **2-Dy**, recorded as a microcrystalline powder, and **2ª-Dy** after heating **2-Dy** at 60 °C and 10^{-3} mbar for 1 hr, recorded as a powder.

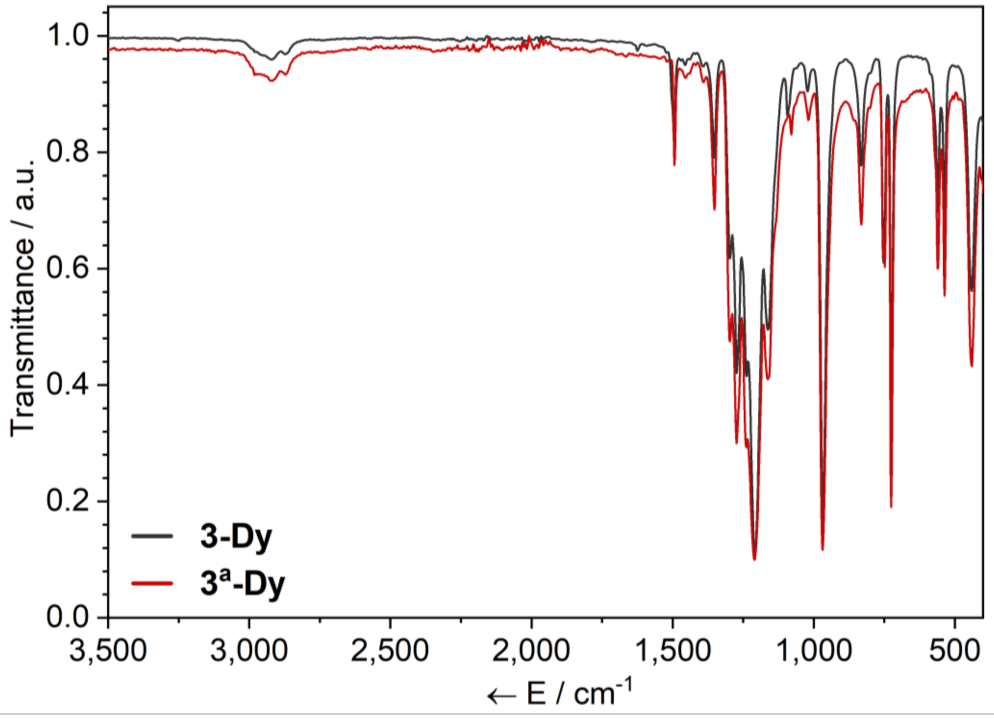

Figure S46. Normalized ATR-IR spectrum of **3-Dy**, recorded as a microcrystalline powder, and **3ª-Dy** after heating **3-Dy** at 60 °C and 10^{-3} mbar for 1 hr, recorded as a powder.

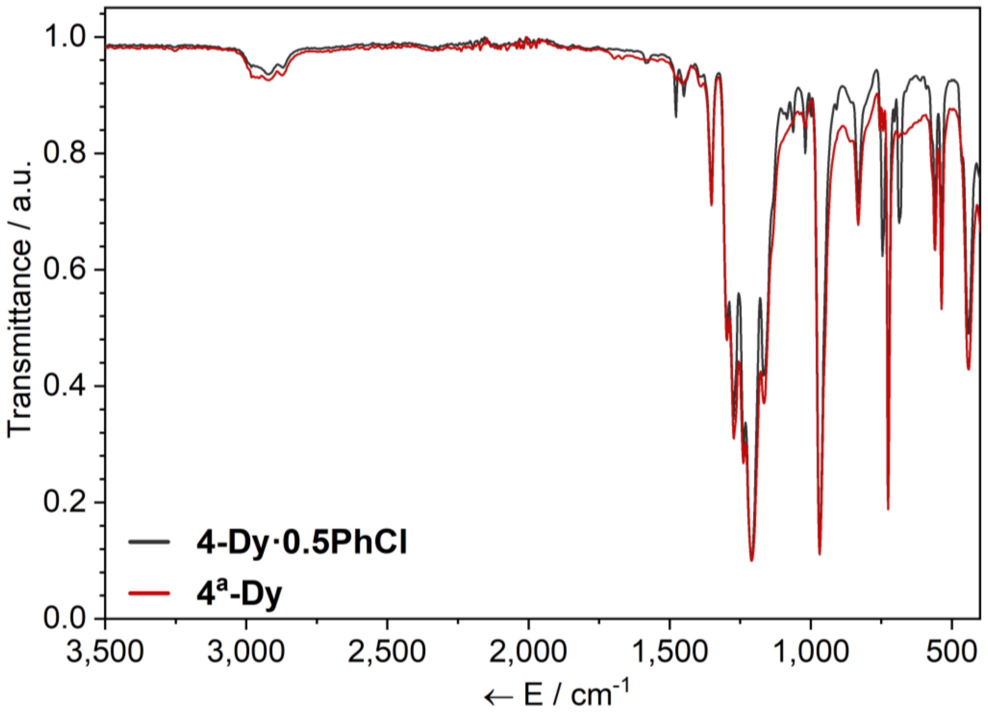

Figure S47. Normalized ATR-IR spectrum of **4-Dy**, recorded as a microcrystalline powder, and **4ª-Dy** after heating **4-Dy** at 60 °C and 10^{-3} mbar for 1 hr, recorded as a powder.

7. DFT Calculations

All DFT calculations were performed using the Orca 4.2.1 or 5.0.3 software package.[14–16] The PBE0 hybrid functional, augmented by Stefan Grimme's D4 dispersion correction,[17,18] the Def2-TZVP basis set,[19–21] and the RIJCOSX approximation were employed throughout. The initial coordinates were obtained through unoptimized XRD data and manually altered to make the Y analogues of the Dy cations. Atom coordinates were verified as minima on the potential energy surface by the absence of imaginary vibrational modes.

Table S10. Calculated atomic coordinates for [Y(Cp*)$_2$][Al{OC(CF$_3$)$_3$}$_4$].

Atom	x	y	z	Atom	x	y	z
Y	–9.865019	3.849854	–0.289539	C	–12.465808	3.488788	–0.202087
C	–7.726454	3.906294	–1.664941	C	–11.856571	2.208638	–0.124561
C	–8.556007	2.942824	–2.302387	C	–11.067502	2.178094	1.054032
C	–9.666725	3.628461	–2.865379	C	–11.223617	3.414247	1.735778
C	–9.53796	5.008489	–2.553949	C	–12.551427	5.614686	1.265404
C	–8.334151	5.180328	–1.826862	H	–12.578645	6.246654	0.372867
C	–6.431816	3.650922	–0.957245	H	–13.569257	5.609344	1.67052
H	–6.303909	2.603001	–0.684366	H	–11.922755	6.105002	2.010817
H	–5.581877	3.920682	–1.591585	C	–13.493802	3.896234	–1.198235
H	–6.327897	4.241546	–0.042832	H	–13.379766	3.346861	–2.13382
C	–8.261753	1.482001	–2.422075	H	–14.495967	3.676891	–0.813623
H	–9.169183	0.881577	–2.486607	H	–13.462426	4.963026	–1.428708
H	–7.672727	1.271934	–3.320313	C	–12.119864	1.064391	–1.046168
H	–7.679718	1.112457	–1.57444	H	–11.286649	0.360572	–1.08988
C	–10.64972	3.055415	–3.829312	H	–12.989998	0.496244	–0.702537
H	–11.627111	3.536488	–3.773171	H	–12.338483	1.396414	–2.061073
H	–10.281122	3.20678	–4.849967	C	–10.019228	1.160331	1.34498
H	–10.787483	1.981636	–3.693229	H	–9.017183	1.614324	1.371666
C	–10.449245	6.10276	–3.003724	H	–10.127984	0.677126	2.317864
H	–10.491386	6.92735	–2.288098	H	–10.007644	0.385777	0.578352
H	–10.111362	6.530751	–3.953472	C	–10.523079	3.792976	3.006223
H	–11.466631	5.739562	–3.155145	H	–10.397816	4.873363	3.110515
C	–7.763661	6.480003	–1.355473	H	–11.037385	3.443718	3.90603
H	–7.150898	6.367409	–0.457834	H	–9.541131	3.328726	3.076167
H	–7.110387	6.913154	–2.119024	Al	–4.262094	1.75531	6.251293
H	–8.537998	7.221363	–1.145898	O	–5.157499	2.925681	7.166351
C	–12.087445	4.22756	0.953009	O	–5.407551	1.244177	5.042922
O	–3.795405	0.342862	7.119072	F	0.594521	1.682298	5.707566
O	–2.845001	2.517298	5.616253	C	–6.441749	1.466175	4.239281
C	–5.19441	3.847052	8.137563	C	–6.17343	0.632398	2.934877
C	–6.707239	4.104246	8.434649	F	–5.664714	–0.550867	3.201565
F	–7.359861	2.953463	8.538912	F	–7.286336	0.425965	2.209359
F	–6.923056	4.801831	9.549849	F	–5.301661	1.276306	2.156085
F	–7.255789	4.779406	7.418768	C	–7.772801	0.997863	4.916964
C	–4.45019	3.353294	9.431053	F	–7.849768	–0.326789	4.929008
F	–5.199813	2.502192	10.131214	F	–7.817608	1.452732	6.148858
F	–3.337864	2.714191	9.078336	F	–8.895745	1.445999	4.301858
F	–4.112308	4.354641	10.246949	C	–6.620885	2.984142	3.853448
C	–4.53624	5.184399	7.667916	F	–7.208334	3.108101	2.624406
F	–4.914626	6.221152	8.419166	F	–7.411552	3.642895	4.698409
F	–3.208377	5.108294	7.719552	F	–5.460057	3.598086	3.800175
F	–4.876725	5.449395	6.412798	C	–4.179526	–0.864605	7.567707
C	–1.623645	2.378712	5.066842	C	–4.345734	–1.901405	6.410406
C	–0.977581	3.787525	4.819579	F	–3.382361	–1.765665	5.512515
F	0.046346	3.71365	3.956797	F	–4.322929	–3.157347	6.866073
F	–1.87591	4.632286	4.324168	F	–5.509697	–1.729829	5.784228
F	–0.498367	4.318259	5.939006	C	–3.083759	–1.369718	8.558665
C	–1.731426	1.640577	3.692854	F	–2.044208	–1.878649	7.894466
F	–2.256406	2.452097	2.767281	F	–2.639755	–0.367572	9.300934
F	–0.561479	1.210279	3.228396	F	–3.559212	–2.320435	9.37175
F	–2.534957	0.587017	3.803422	C	–5.526537	–0.754439	8.346298
C	–0.699727	1.56011	6.018484	F	–6.099414	–1.934651	8.580525
F	–0.866366	1.973824	7.262444	F	–5.332063	–0.151444	9.517141
F	–1.010784	0.27022	5.961553	F	–6.390506	–0.014936	7.659741

Table S11. Calculated atomic coordinates for $[Y(Cp^*)_2(PhF\text{-}\kappa\text{-}F)_2]^+$ (**2′-Y**).

Atom	x	y	z	Atom	x	y	z
Y	–0.4287367	19.4519502	27.8949845	H	0.5091128	19.5772260	23.4104356
C	–1.2930000	20.6791490	25.7666492	C	–3.6739373	17.4734660	32.4036397
F	0.0767938	17.1126927	28.4096839	H	–3.5070788	16.6998305	33.1429833
C	0.1704597	20.9492705	29.9432471	C	–3.3923291	17.7184727	26.6812846
C	1.6496778	20.9657050	28.1864548	H	–3.0100333	16.6981485	26.7173697
F	–2.0536062	18.8683981	29.4829436	H	–3.8395422	17.9407992	27.6535880
C	–0.8517906	21.4118334	30.9293964	H	–4.2088939	17.7263676	25.9526310
H	–1.7105683	21.8857804	30.4469835	C	0.4207401	13.5450736	27.9486657
H	–1.2271425	20.5988927	31.5517217	H	1.1690668	12.8285495	27.6336272
H	–0.4241082	22.1581971	31.6063794	C	–4.8167012	18.2572313	32.4571358
C	0.9475402	18.7913488	31.1546062	H	–5.5465127	18.0940093	33.2402203
H	–0.0100066	18.7968405	31.6771699	C	0.7407503	14.8952539	27.9775286
H	1.1381557	17.7721497	30.8097978	H	1.7196012	15.2594219	27.6932045
H	1.7152029	19.0199684	31.9003377	C	–1.5063342	15.3888940	28.7593605
C	–1.1153316	18.3934858	25.6273305	H	–2.2419955	16.1208163	29.0631106
C	–2.4338078	20.1090413	26.3868642	C	–2.9853801	18.6673461	30.4898650
C	2.5036399	21.4555795	27.0647213	C	1.8893740	19.7969187	28.9555525
H	2.8991210	20.6395335	26.4576003	C	–0.8453753	13.1151295	28.3192701
H	1.9614633	22.1313429	26.4029861	H	–1.0862869	12.0598200	28.2931389
H	3.3632510	22.0098641	27.4545407	C	0.5817394	21.6777874	28.7991203
C	0.9766237	19.7883832	30.0429138	C	–5.0260544	19.2521105	31.5132602
C	–1.1367073	22.1243273	25.4340063	H	–5.9170828	19.8661032	31.5575113
H	–1.5985023	22.7723941	26.1789561	C	–0.6429453	17.0436297	25.1959241
H	–0.0926796	22.4202393	25.3310753	H	0.4380126	16.9244453	25.3058955
H	–1.6246026	22.3391024	24.4773718	H	–1.1232728	16.2384863	25.7522775
C	–0.2408423	15.7666255	28.3811012	H	–0.8677979	16.8811074	24.1370870
C	–0.4681774	19.6174245	25.3108836	C	–1.8029833	14.0325218	28.7237780
C	2.9763399	18.7978327	28.7243301	H	–2.7916932	13.6980011	29.0133195
H	2.7216757	17.8187630	29.1353061	C	–4.0984767	19.4714193	30.5037720
H	3.2052185	18.6706203	27.6634644	H	–4.2362706	20.2448144	29.7602187
H	3.9051991	19.1129237	29.2102916	C	–2.3319159	18.6957654	26.2888961
C	–3.6118934	20.8694697	26.9063219	C	0.1183935	23.0579384	28.4708635
H	–4.3078015	20.2138641	27.4315957	H	0.3968859	23.3598770	27.4630517
H	–3.3318927	21.6811053	27.5850239	H	–0.9646627	23.1695081	28.5657839
H	–4.1707392	21.3299529	26.0857330	H	0.5684435	23.7791421	29.1607257
C	0.7550163	19.7439260	24.4640972	C	–2.7302940	17.6738639	31.4043389
H	1.2016795	20.7345121	24.5387414	H	–1.8263151	17.0820145	31.3459967
H	1.5239461	19.0122009	24.7256059				

Table S12. Calculated atomic coordinates for [Y(Cp*)$_2$(C$_6$H$_4$F$_2$-κ^2-*F*,*F*)(C$_6$H$_4$F$_2$-κ-*F*)]$^+$ (**3′-Y**).

Atom	x	y	z	Atom	x	y	z
Y	16.2946827	13.1280309	–4.3222885	H	16.8770072	12.5969727	–8.2658856
F	14.2908368	12.0464996	–3.2296742	C	15.9768079	15.1040383	–7.4365681
F	16.2215185	13.0315018	–1.7935009	H	15.7679543	16.1129874	–7.8072022
F	18.2906333	14.0478345	–3.2554250	H	15.3780096	14.4148153	–8.0283428
F	18.7282168	12.9119192	–0.8309261	H	17.0293515	14.9033411	–7.6472944
C	16.6951636	11.5683625	–6.3807300	C	14.6083771	10.1975680	–5.7126467
C	16.7813651	10.5441486	–4.3288028	H	14.1172957	9.8828897	–4.7911377
C	17.9742397	11.8230899	–5.8162633	H	14.6959592	9.3098570	–6.3472595
C	15.6568020	15.0286015	–5.9819427	H	13.9384904	10.8900170	–6.2268022
C	15.2829807	12.2997200	–1.1378187	C	15.3601700	12.0783807	0.2139356
C	15.9535898	10.7927425	–5.4544008	H	16.1826592	12.4896472	0.7851613
C	18.0294133	11.1713257	–4.5568321	C	15.6742015	15.6056357	–3.7592688
C	16.4192309	15.6541969	–4.9647055	C	13.2584097	11.0325410	–1.3348274
C	19.1211945	12.4936645	–6.5004469	H	12.4608096	10.6378549	–1.9513825
H	18.7907770	13.2624659	–7.2013649	C	13.3189768	10.8008300	0.0341293
H	19.7107153	11.7720462	–7.0753050	H	12.5447714	10.2083354	0.5045212
H	19.8055269	12.9629596	–5.7901689	C	14.3565010	11.3150880	0.7979515
C	19.5371568	14.1001249	–2.6913830	H	14.3905119	11.1220297	1.8625668
C	16.4824615	9.6294584	–3.1857901	C	13.2905806	13.9568199	–6.1278278
H	16.8867347	9.9917784	–2.2371876	H	13.6129704	13.4399088	–7.0324361
H	16.9314085	8.6458729	–3.3594704	H	12.5650027	14.7172223	–6.4330649
H	15.4126118	9.4647870	–3.0537489	H	12.7520328	13.2353319	–5.5088673
C	14.4482038	14.9504458	–4.0329416	C	16.0514911	16.2343343	–2.4580239
C	17.7042448	16.3822413	–5.1839370	H	15.6154294	15.7086612	–1.6061769
H	18.2199345	16.5887992	–4.2455193	H	15.6948002	17.2678467	–2.4073304
H	17.5217338	17.3479577	–5.6657350	H	17.1323152	16.2643920	–2.3097294
H	18.3888371	15.8322794	–5.8359144	C	13.3112730	14.8140884	–3.0749406
C	14.2579758	11.7853864	–1.8971705	H	12.6139618	14.0313144	–3.3747471
C	19.2406115	10.9859184	–3.7048464	H	12.7411238	15.7469593	–3.0256249
H	20.0160948	11.7213377	–3.9185007	H	13.6423757	14.6004657	–2.0552459
H	19.6812155	10.0009119	–3.8923408	C	19.7378079	13.5210562	–1.4525926
H	19.0146178	11.0255404	–2.6370059	C	22.0309496	14.1760576	–1.5532572
C	14.4404729	14.5745450	–5.4015805	H	23.0154860	14.2017707	–1.1036928
C	20.5558251	14.7211124	–3.3734469	C	21.8161133	14.7567206	–2.7935908
H	20.3602710	15.1647259	–4.3409636	H	22.6297740	15.2406834	–3.3180271
C	16.3001386	11.8004486	–7.7996642	C	20.9908437	13.5559012	–0.8782627
H	15.2412545	12.0396371	–7.9096642	H	21.1340016	13.0899580	0.0890214
H	16.4795116	10.8904013	–8.3820006				

Table S13. Calculated atomic coordinates for $[Y(Cp^*)_2(PhCl\text{-}\kappa\text{-}Cl)_2]^+$ (**4′-Y**).

Atom	x	y	z	Atom	x	y	z
Y	0.2252979	6.2166031	12.3820378	H	–2.6395731	8.8976383	11.7513707
Cl	3.1248355	5.9058031	12.0204499	H	–2.9317720	9.4427761	13.3936246
Cl	0.3664092	3.8159589	13.9062619	H	–3.2775366	7.7780999	12.9557614
C	0.3379324	4.4655432	10.4244568	C	0.1311289	9.8274105	11.6060116
C	0.4531203	5.7511214	9.8310655	H	0.9892492	9.6525660	10.9520227
C	–0.7885823	6.4210497	9.9886170	H	0.2700664	10.8205291	12.0457861
C	–1.6640029	5.5604263	10.7015964	H	–0.7600251	9.8732130	10.9832750
C	–0.9701579	4.3504663	10.9612100	C	2.4659792	8.9584819	13.4570748
C	1.3352575	3.3580786	10.3318566	H	3.1590317	8.3928627	14.0791305
H	2.3411042	3.7312404	10.1365477	H	2.4809682	9.9912040	13.8211756
H	1.0803070	2.6845807	9.5076534	H	2.8651482	8.9769998	12.4407714
H	1.3698186	2.7479797	11.2379085	C	1.3098512	7.1414609	15.7709619
C	1.5994612	6.2587351	9.0165373	H	0.7473658	6.4166363	16.3577036
H	1.7984520	7.3200406	9.1881241	H	1.5261349	7.9889999	16.4292038
H	1.3883508	6.1502514	7.9479413	H	2.2682580	6.6817686	15.5193529
H	2.5210457	5.7114639	9.2151435	C	0.6616286	3.7543090	15.6357243
C	–1.1682069	7.6798652	9.2848790	C	–0.4277941	3.6515901	16.4791172
H	–1.9850446	8.2037779	9.7799431	H	–1.4341911	3.6227063	16.0825762
H	–1.5072263	7.4481474	8.2696706	C	–0.1914859	3.5790499	17.8439759
H	–0.3306893	8.3707557	9.1903937	H	–1.0302150	3.4981606	18.5245730
C	–3.1279229	5.7570351	10.9178672	C	1.1064805	3.6014155	18.3331183
H	–3.4518606	5.4725990	11.9226694	H	1.2820371	3.5426811	19.4001957
H	–3.6985792	5.1373683	10.2184808	C	2.1787594	3.6871795	17.4578323
H	–3.4283607	6.7899392	10.7520571	H	3.1942206	3.6940273	17.8350865
C	–1.6102800	3.1020396	11.4743844	C	1.9643696	3.7631260	16.0902866
H	–0.8764930	2.3499988	11.7648385	H	2.7921877	3.8261211	15.3987514
H	–2.2328571	2.6558944	10.6918295	C	4.0281848	4.9941647	13.2110709
H	–2.2624880	3.2822877	12.3317677	C	4.1314827	3.6245519	13.0507584
C	–0.8504260	7.4944316	14.3621003	H	3.6334531	3.1279101	12.2289388
C	–1.1849582	8.2431299	13.2039598	C	4.8974338	2.9112089	13.9610023
C	0.0130807	8.8020737	12.6843759	H	4.9950477	1.8384880	13.8473327
C	1.0796821	8.4100467	13.5308204	C	5.5380012	3.5651892	15.0038762
C	0.5507483	7.6042450	14.5707512	H	6.1394034	3.0019936	15.7068770
C	–1.8386372	6.8455874	15.2766882	C	5.4193576	4.9405320	15.1391939
H	–2.6043151	6.2804519	14.7376244	H	5.9274936	5.4569318	15.9445548
H	–2.3673778	7.5980674	15.8705894	C	4.6618077	5.6717764	14.2357645
H	–1.3542490	6.1662821	15.9775641	H	4.5841576	6.7472407	14.3173539
C	–2.5753203	8.5967479	12.7965037				

Table S14. Calculated atomic coordinates for $[Y(Cp^*)_2(PhI\text{-}\kappa\text{-}I)_2]^+$ (**15′-Y**).

Atom	x	y	z	Atom	x	y	z
Y	0.4058970	5.9957118	12.3844327	H	–2.2110985	8.9700797	12.3244445
I	3.3282230	5.0481688	11.4409416	H	–2.1877809	9.4793412	14.0040446
I	1.0811915	3.2708498	14.0415272	H	–2.8048449	7.8901561	13.5865049
C	–0.0345968	4.3333366	10.3660183	C	0.6182463	9.6232618	11.8625621
C	0.1291917	5.6256427	9.7957989	H	1.3226822	9.3797596	11.0624328
C	–0.9644108	6.4308373	10.2063780	H	0.9719138	10.5542650	12.3173131
C	–1.8039779	5.6394185	11.0361207	H	–0.3455589	9.8350780	11.4027600
C	–1.2264056	4.3497785	11.1349439	C	3.0552793	8.3784767	13.3475364
C	0.7362328	3.0971168	10.0310329	H	3.7528300	7.6747589	13.7994848
H	1.6932932	3.3144436	9.5553499	H	3.2443153	9.3569880	13.8022754
H	0.1694028	2.4812092	9.3255195	H	3.3091056	8.4703246	12.2888291
H	0.9342064	2.4714835	10.9048877	C	1.9917285	6.5270139	15.6704865
C	1.1356854	6.0347389	8.7686889	H	1.4542442	5.7503522	16.2141297
H	1.5859464	7.0085843	8.9789383	H	2.2927631	7.2812653	16.4048783
H	0.6616581	6.1137082	7.7851853	H	2.9076564	6.0864644	15.2726348
H	1.9465962	5.3121128	8.6705808	C	–0.1356306	3.1321603	15.7465658
C	–1.2910943	7.7664694	9.6269826	C	–1.4877902	2.8827230	15.5940383
H	–1.9639233	8.3415575	10.2619885	H	–1.9228768	2.7476144	14.6131466
H	–1.7905127	7.6419940	8.6602963	C	–2.2767501	2.7995387	16.7320965
H	–0.4005323	8.3700855	9.4494320	H	–3.3374229	2.6036602	16.6295583
C	–3.1750589	5.9849512	11.5157938	C	–1.7134419	2.9583707	17.9891328
H	–3.3388318	5.7194348	12.5634954	H	–2.3354593	2.8905980	18.8731584
H	–3.9243973	5.4398138	10.9330248	C	–0.3529676	3.1933044	18.1173213
H	–3.3908793	7.0457233	11.4056252	H	0.0922908	3.3068680	19.0984381
C	–1.9008540	3.1569406	11.7254488	C	0.4525423	3.2806785	16.9906186
H	–1.1915476	2.3977657	12.0574094	H	1.5158275	3.4569031	17.0900810
H	–2.5512825	2.6844635	10.9810077	C	4.9077382	5.4514049	12.7634790
H	–2.5399366	3.4253099	12.5693172	C	4.9840115	4.7693253	13.9643222
C	–0.2715747	7.2047404	14.5801120	H	4.2367615	4.0370518	14.2412343
C	–0.6574682	8.0811750	13.5319125	C	6.0514773	5.0396747	14.8092939
C	0.5219579	8.5481952	12.8943960	H	6.1281948	4.5132728	15.7531992
C	1.6339477	7.9789073	13.5647466	C	7.0164401	5.9663577	14.4462340
C	1.1478733	7.1530350	14.6100074	H	7.8477052	6.1694307	15.1101557
C	–1.2069387	6.5912033	15.5716719	C	6.9239181	6.6274408	13.2305612
H	–2.0797516	6.1305457	15.1007443	H	7.6801140	7.3470021	12.9403907
H	–1.5885156	7.3501118	16.2620955	C	5.8628512	6.3742178	12.3740196
H	–0.7182317	5.8246387	16.1719452	H	5.7878948	6.8907595	11.4258237
C	–2.0345246	8.6209688	13.3413713				

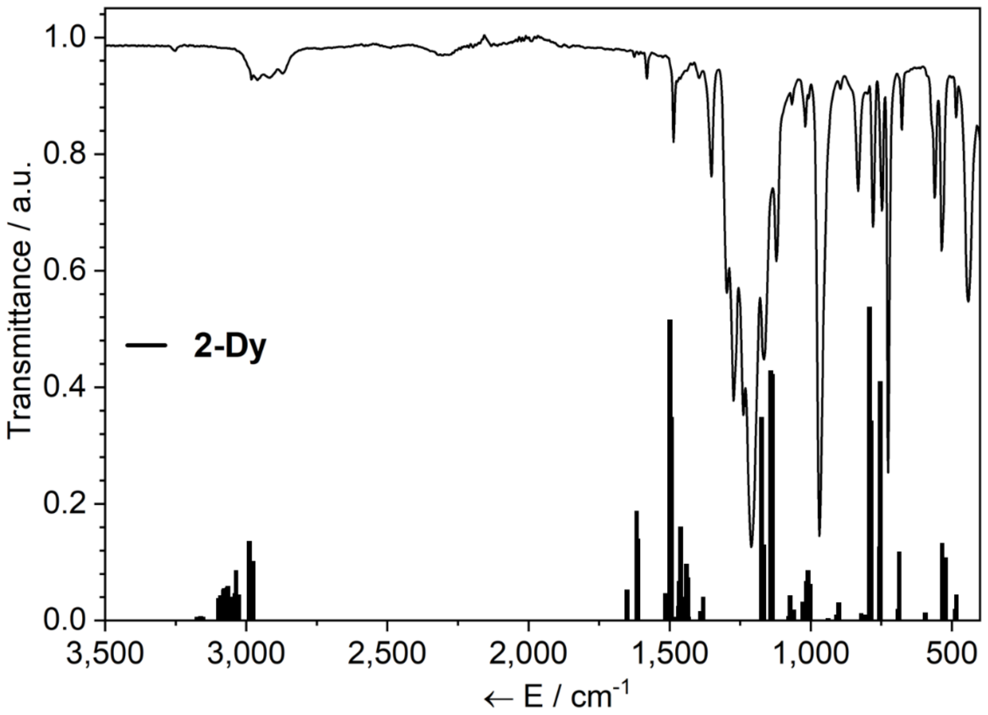

Figure S48. Overlay of the simulated IR spectrum of **2′-Y** (0.975 scale) and the measured ATR-IR spectrum of **2-Dy**.

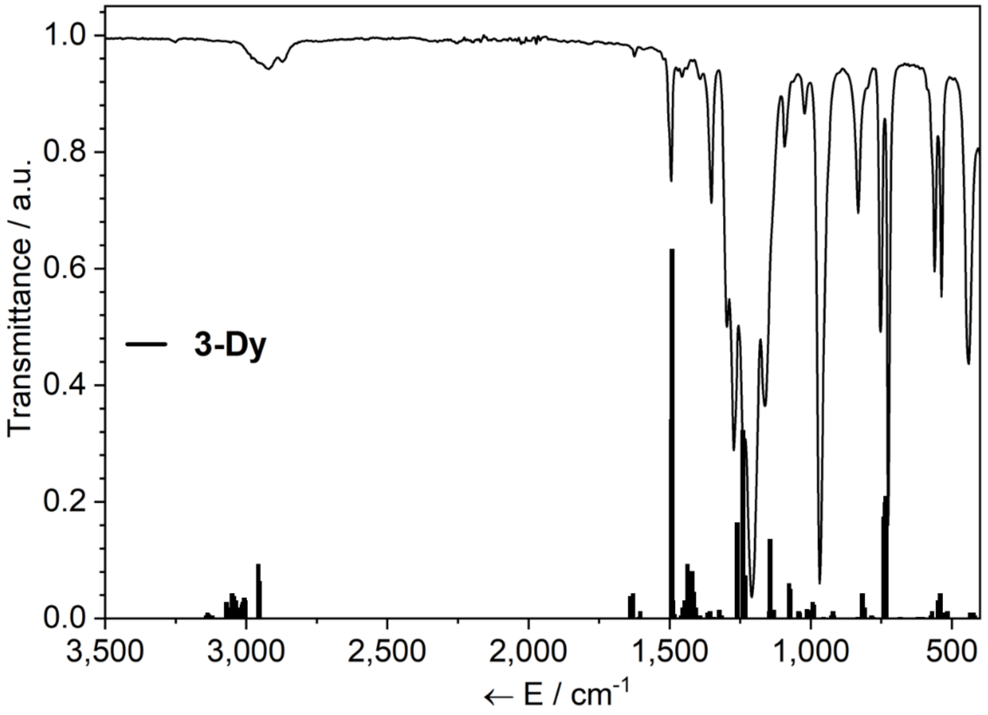

Figure S49. Overlay of the simulated IR spectrum of **3′-Y** (0.98 scale) and the measured ATR-IR spectrum of **3-Dy**.

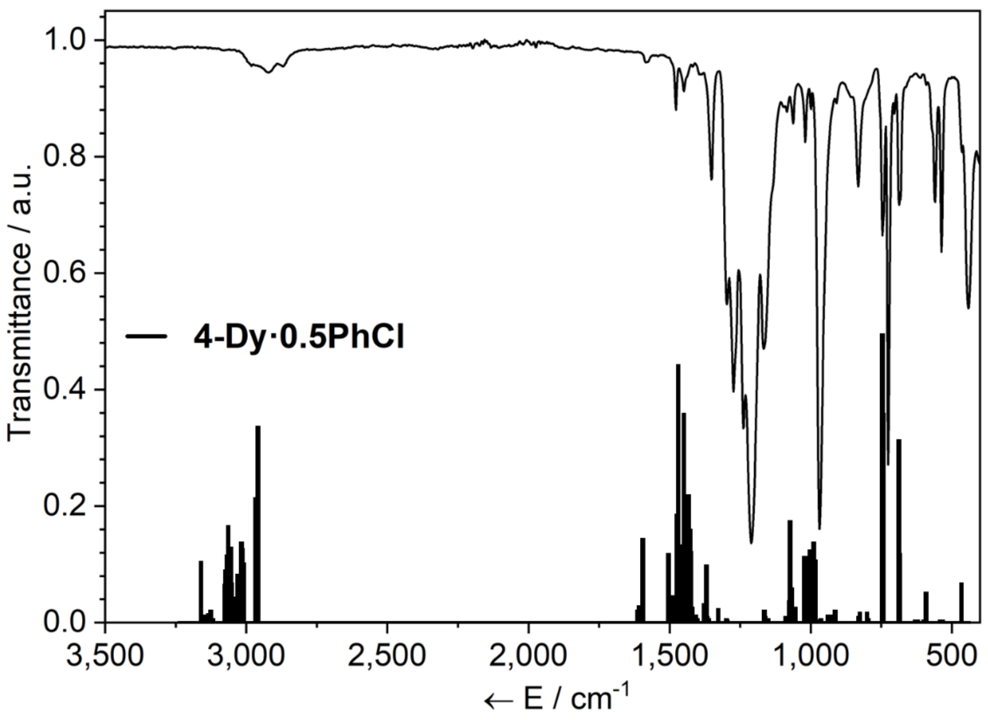

Figure S50. Overlay of the simulated IR spectrum of **4′-Y** (0.995 scale) and the measured ATR-IR spectrum of **4-Dy**.

8. Magnetic Measurements

Magnetic measurements were performed using a Quantum Design MPMS3 superconducting quantum interference device (SQUID) magnetometer. All samples were crushed with a mortar and pestle under argon, and then loaded into a borosilicate glass NMR tube along with eicosane, which was then evacuated and flame-sealed to a length of *ca.* 3 cm. The eicosane was melted by heating the tube gently with a low-power heat gun in order to immobilize the crystallites. The NMR tube was then mounted in the center of a drinking straw using friction by wrapping it with Kapton tape, and the straw was then fixed to the end of the sample rod. Samples of "[{Dy(Cp*)$_2$}{Al[OC(CF$_3$)$_3$]$_4$}]" (19.9 mg), **2-Dy** (24.4 mg), **3-Dy** (22.9 mg) and **4-Dy** (17.0, 18.1 and 22.7 mg) were prepared with the following amounts of eicosane ("[{Dy(Cp*)$_2$}{Al[OC(CF$_3$)$_3$]$_4$}]": 12.8 mg; **2-Dy**: 15.5 mg; **3-Dy**: 14.9 mg: **4-Dy**: 13.2, 14.2 and 13.4 mg). The measurements were corrected for the diamagnetism of the straw, borosilicate tube and eicosane using calibrated blanks, for the shape of the sample using Quantum Design Geometry Simulator, and for the intrinsic diamagnetism of the sample estimated as the molecular weight (g mol^{-1}) multiplied by -0.5×10^{-6} cm^3 K mol^{-1}.

All dc magnetic measurements were performed in dc scan mode with a scan length of 40 mm and a scan time of 6 s. The equilibrium magnetic susceptibility was measured under 0.1 T field, on cooling in temperature settle mode, at 5 K min^{-1} from 300–100 K and 1 K min^{-1} from 100–1.8 K. Equilibrium magnetization vs field measurements held the temperature and field stable for a minimum of 5 mins (0–3.5 T) or 3 mins (4–7 T) before each measurement. Hysteresis measurements on "[{Dy(Cp*)$_2$}{Al[OC(CF$_3$)$_3$]$_4$}]" were performed at 2 and 4 K, ±7 T in continuous sweep mode on a sample that had been magnetized at 7 T. The sweep rates for "[{Dy(Cp*)$_2$}{Al[OC(CF$_3$)$_3$]$_4$}]" were 22 Oe s^{-1} for $|H| < 1$ T, 54 Oe s^{-1} for $1 < |H| < 2$ T, and 91 Oe s^{-1} for $2 < |H| < 7$ T. For **2-Dy**, **3-Dy** and **4-Dy** hysteresis measurements were performed between ± 5 T at temperatures of 2–10 K (and 12 K for **4-Dy**) on samples that had

been magnetized at 5 T. Measurements were performed in continuous sweep mode with a sweep rate of 22 Oe s^{-1} across the entire field range. Slow thermal equilibration was noticeable for "[{Dy(Cp*)$_2$}{Al[OC(CF$_3$)$_3$]$_4$}]" and **4-Dy**; to ensure thermal equilibration of these sample at each ac measurement temperature, the ac susceptibility in zero dc field was monitored as a function of time until it was steady. Frequency-dependent ac measurements (0.1–1000 Hz) were performed in zero dc field with 5 Oe oscillating field, or 2 Oe oscillating field for 750 and 1000 Hz due to instrumental limitations. Frequency-dependent ac measurements on "[{Dy(Cp*)$_2$}{Al[OC(CF$_3$)$_3$]$_4$}]" were performed in zero dc field with a 2 Oe oscillating field.

The magnetic properties of "[{Dy(Cp*)$_2$}{Al[OC(CF$_3$)$_3$]$_4$}]" were investigated by SQUID magnetometry. Firstly, low temperature magnetization vs field measurements of "[{Dy(Cp*)$_2$}{Al[OC(CF$_3$)$_3$]$_4$}]" reveal a well-isolated ground state and a saturation value of 4.69 N μ_B, which is less than the expected value of 5.00 N μ_B for a pure $m_J = \pm 15/2$ ground state, consistent with the presence of triethylamine. Additionally, the room temperature magnetic susceptibility ($\chi_M T$) is 12.8 cm^3 K mol^{-1}. The $\chi_M T$ value decreases monotonously with temperature towards 10.8 cm^3 K mol^{-1}, which is a result of excited crystal field states being thermally depopulated; the sharp decrease below 18 K is attributed to slow equilibration, which may be a combination of slow thermal equilibration of the sample and magnetic blocking.

The dynamic magnetic behaviour of "[{Dy(Cp*)$_2$}{Al[OC(CF$_3$)$_3$]$_4$}]" was examined *via* low temperature ac susceptibility measurements at zero-field; the in-phase (χ_M') and out-of-phase (χ_M'') ac susceptibility signals are indicative of slow relaxation between 2 and 22 K. Subsequently, the ac data has been fit to a generalised Debye model in CC-FIT2[22,23] to determine the dependence of relaxation rate on temperature. Raman relaxation processes ($\tau^{-1} = 10^R T^n$) are operative above approximately 8 K [R = –0.43(2) log[s^{-1} K^{-n}]; n = 3.0(1)]. Below 8 K, QTM processes dominate [$\tau_{QTM} = 10^Q = 10^{-3.08(1)}$ s].

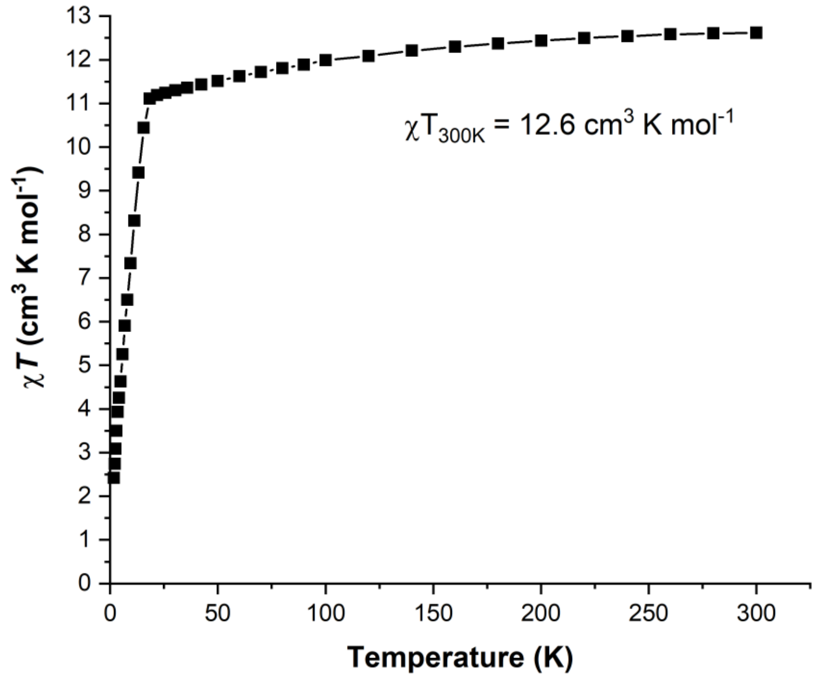

Figure S51. Temperature dependence of the molar magnetic susceptibility $\chi_M T$ products of "[{Dy(Cp*)$_2$}{Al[OC(CF$_3$)$_3$]$_4$}]" measured under a 0.1 T DC field.

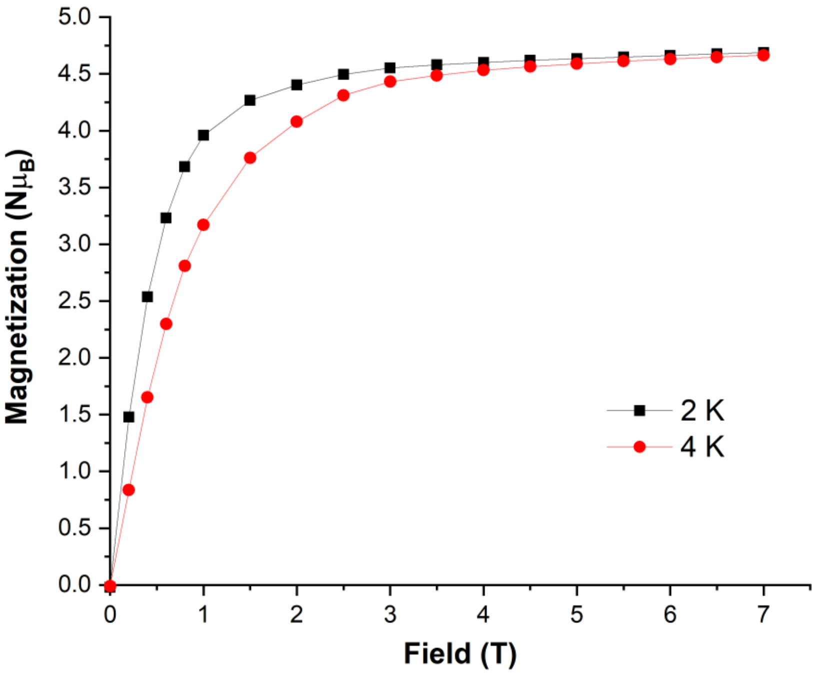

Figure S52. Magnetization *vs* field curves for "[{Dy(Cp*)$_2$}{Al[OC(CF$_3$)$_3$]$_4$}]".

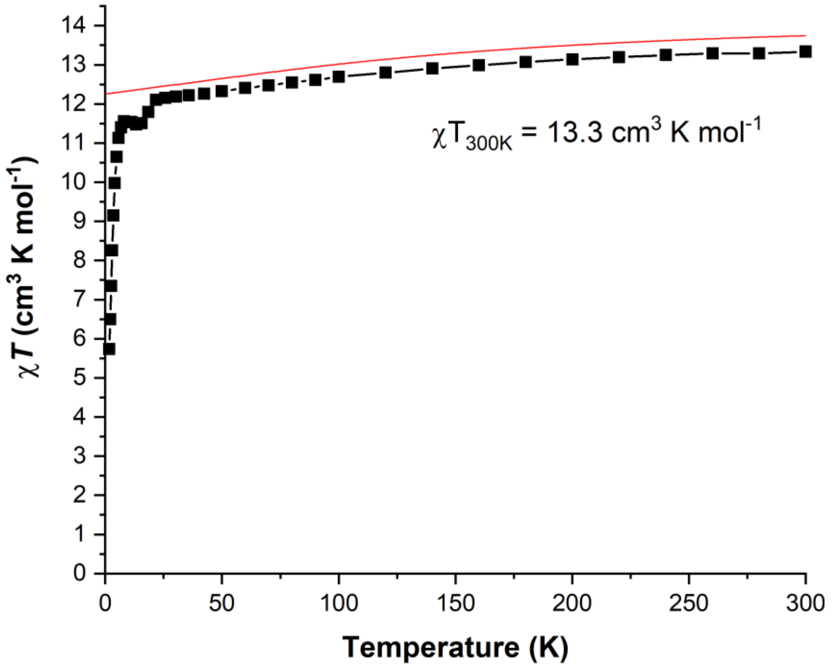

Figure S53. Temperature dependence of the molar magnetic susceptibility χ_MT product for powdered **2-Dy** measured under a 0.1 T applied magnetic field (black) and predicted values from CASSCF (red).

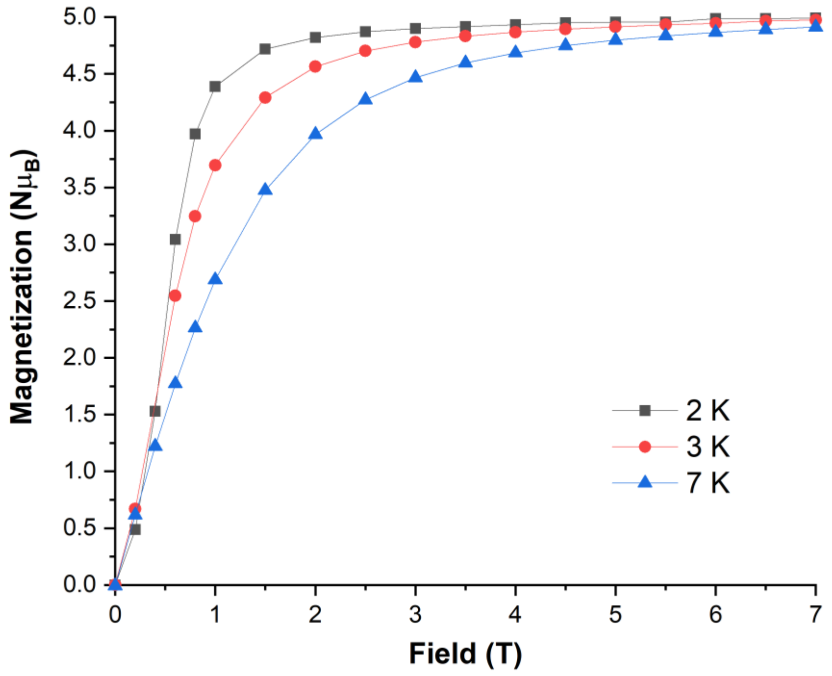

Figure S54. Magnetization *vs* field curves for **2-Dy**.

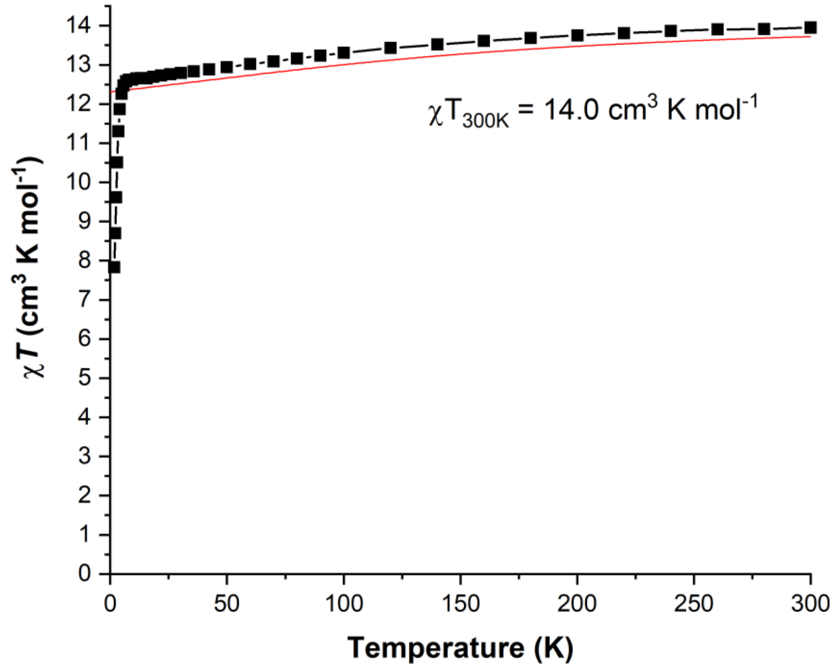

Figure S55. Temperature dependence of the molar magnetic susceptibility $\chi_M T$ product for powdered **3-Dy** measured under a 0.1 T applied magnetic field (black) and predicted values from CASSCF (red).

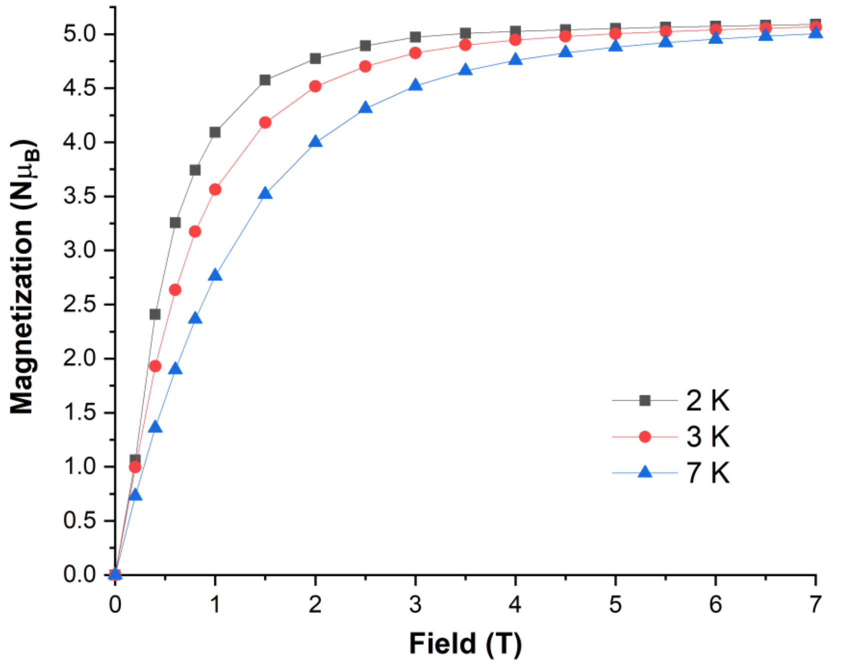

Figure S56. Magnetization *vs* field curves for **3-Dy**.

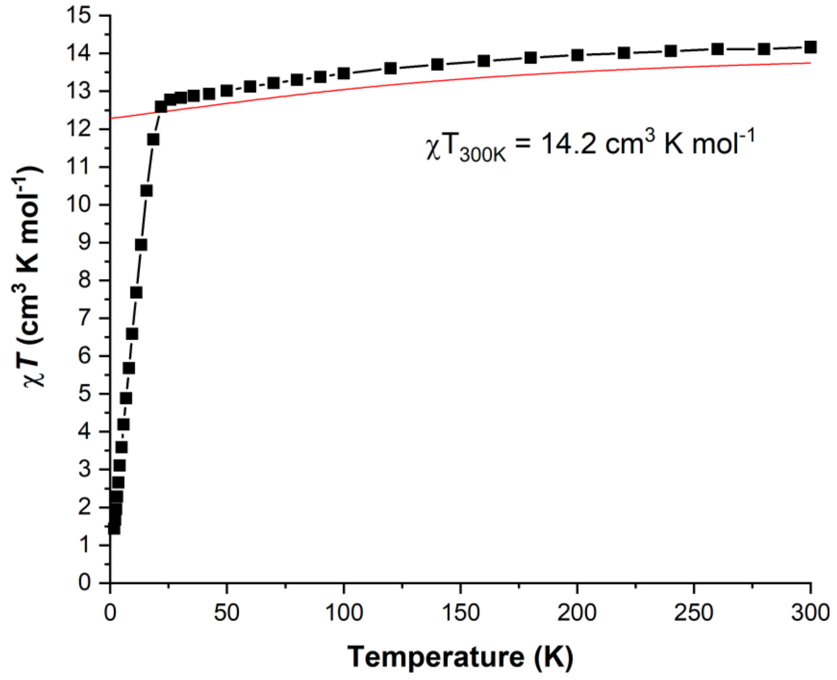

Figure S57. Temperature dependence of the molar magnetic susceptibility $\chi_M T$ product for powdered **4-Dy** measured under a 0.1 T applied magnetic field (black) and predicted values from CASSCF (red).

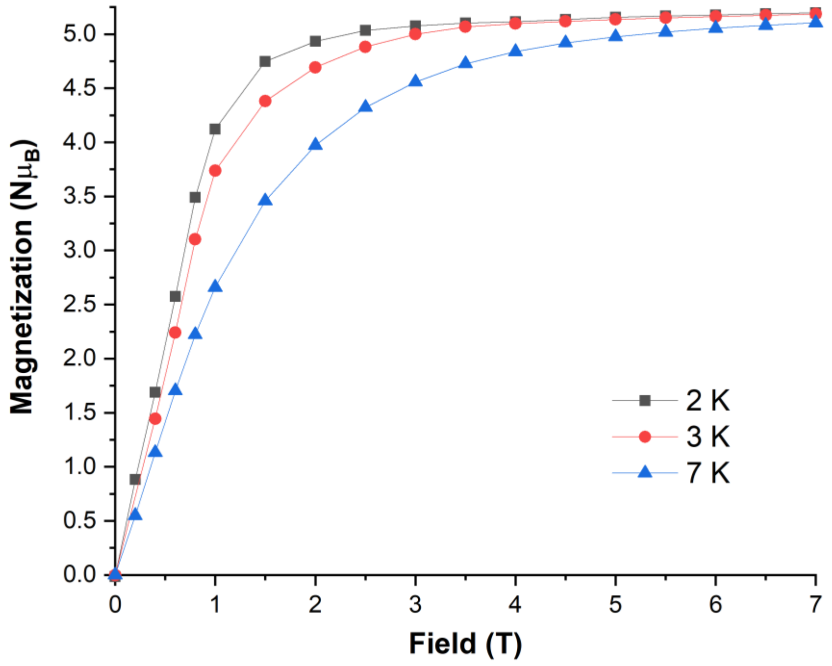

Figure S58. Magnetization *vs* field curves for **4-Dy**.

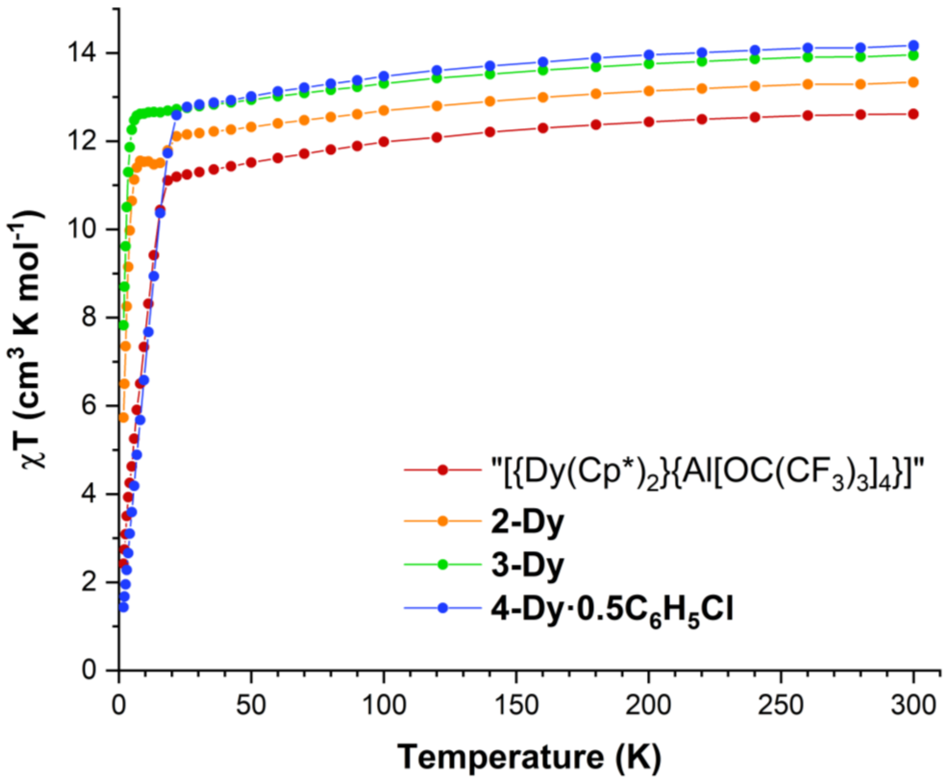

Figure S59. Combined temperature dependence of the molar magnetic susceptibility $\chi_M T$ products under a 0.1 T applied magnetic field.

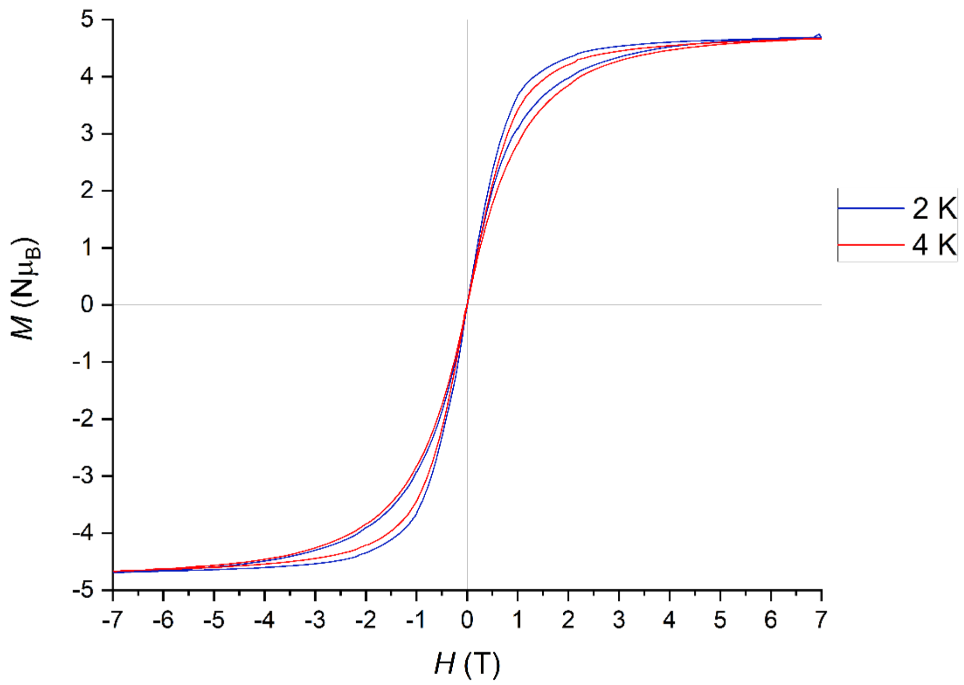

Figure S60. Temperature dependence of hysteresis loops of "[{Dy(Cp*)$_2$}{Al[OC(CF$_3$)$_3$]$_4$}]". Sweep rate is 22 Oe s^{-1} for |H| < 1 T, 54 Oe s^{-1} for 1 < |H| < 2 T, and 91 Oe s^{-1} for 2 < |H| < 7 T.

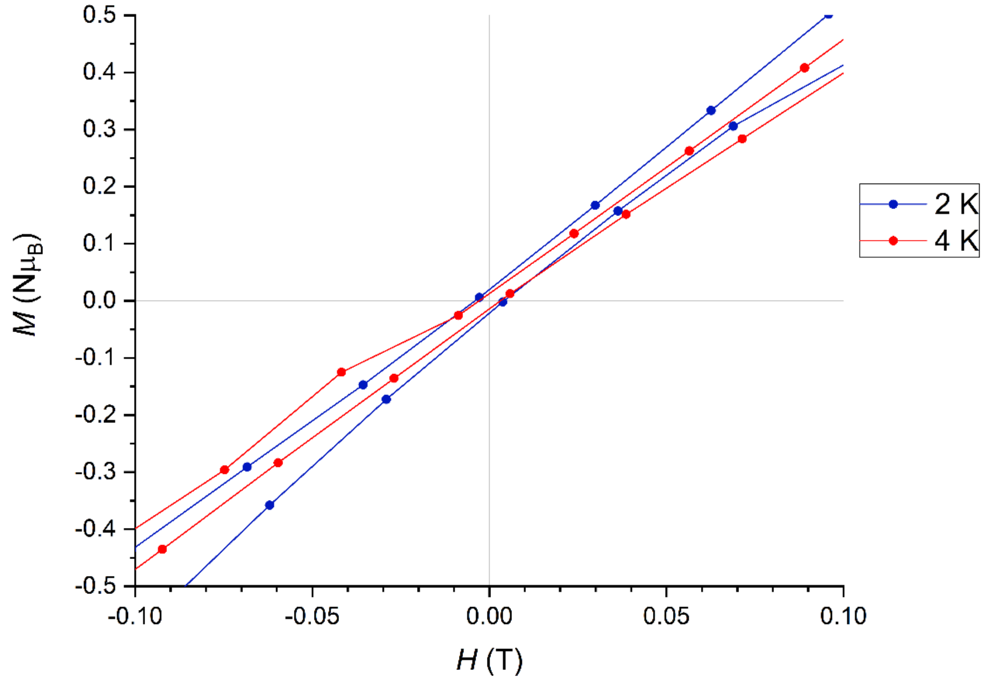

Figure S61. Hysteresis loops of "[{Dy(Cp*)$_2$}{Al[OC(CF$_3$)$_3$]$_4$}]" at 2 and 4 K zoomed to –0.1 T to +0.1 T. Sweep rate is 22 Oe s^{-1} for |H| < 1 T, 54 Oe s^{-1} for 1 < |H| < 2 T, and 91 Oe s^{-1} for 2 < |H| < 7 T.

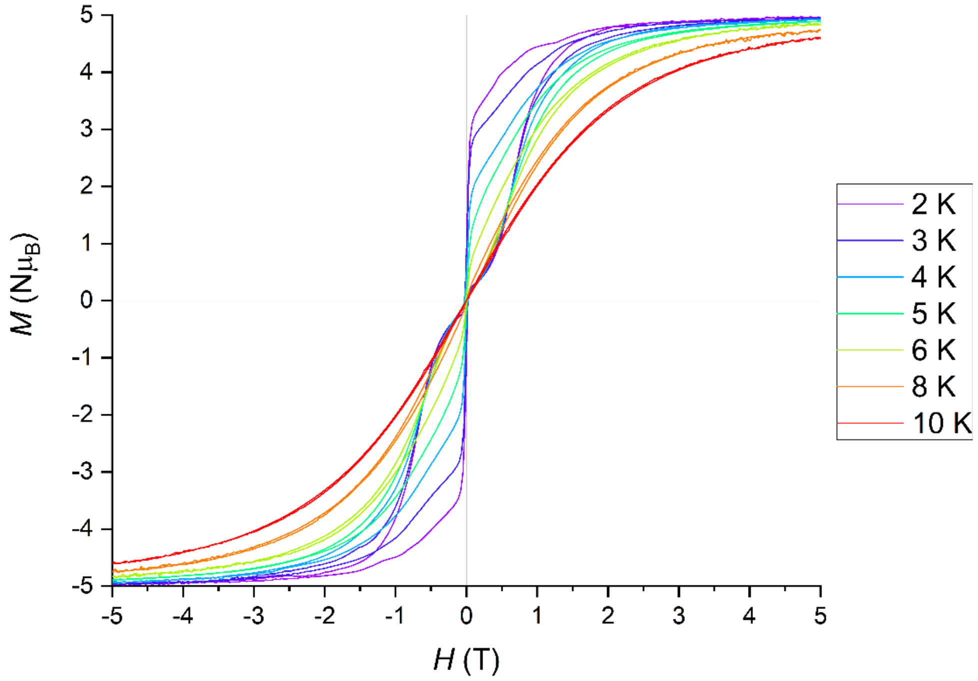

Figure S62. Hysteresis loops of **2-Dy** from 2 to 10 K and –5 T to +5 T. Sweep rate is 22 Oe s^{-1}.

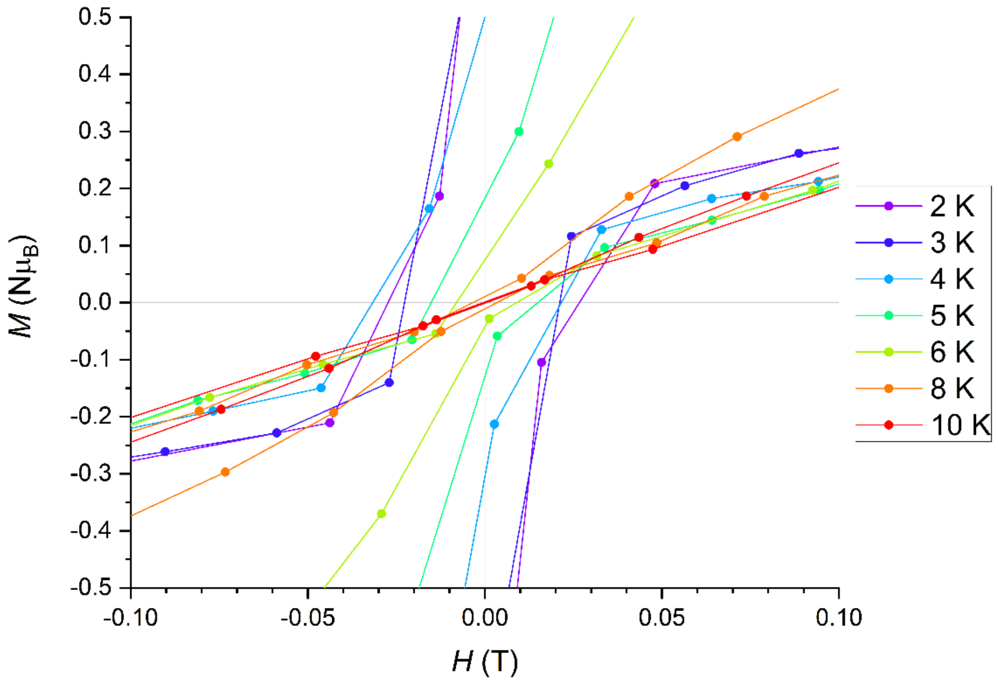

Figure S63. Hysteresis loops of **2-Dy** from 2 to 10 K zoomed to –0.1 T to +0.1 T. Sweep rate is 22 Oe s^{-1}.

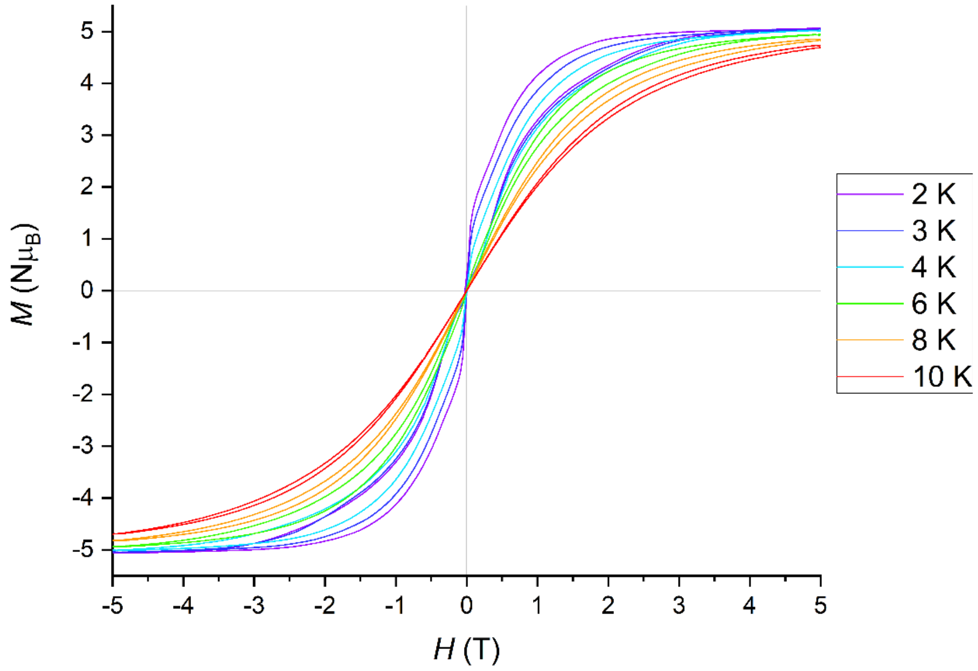

Figure S64. Hysteresis loops of **3-Dy** from 2 to 10 K and –5 T to +5 T. Sweep rate is 22 Oe s^{-1}.

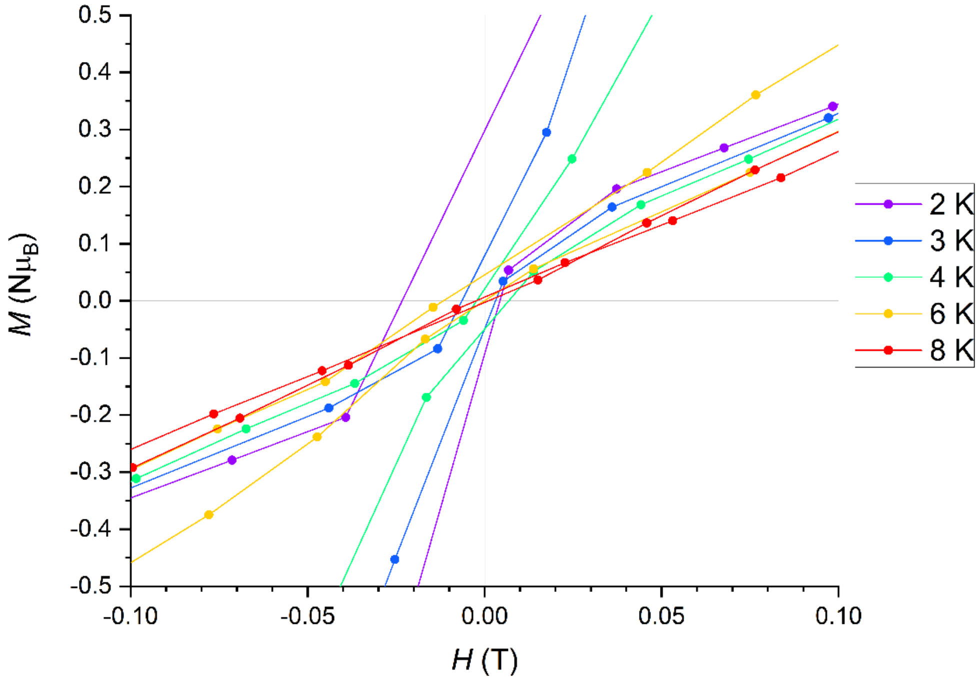

Figure S65. Hysteresis loops of **3-Dy** from 2 to 8 K zoomed to –0.1 T to +0.1 T. Sweep rate is 22 Oe s^{-1}.

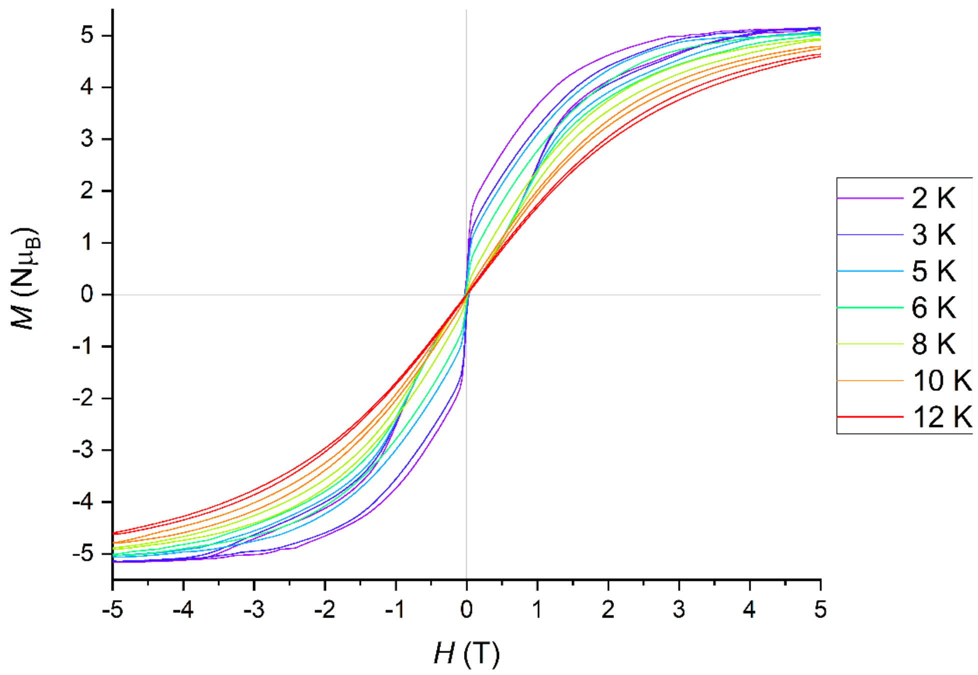

Figure S66. Hysteresis loops of **4-Dy** from 2 to 12 K and –5 T to +5 T. Sweep rate is 22 Oe s^{-1}.

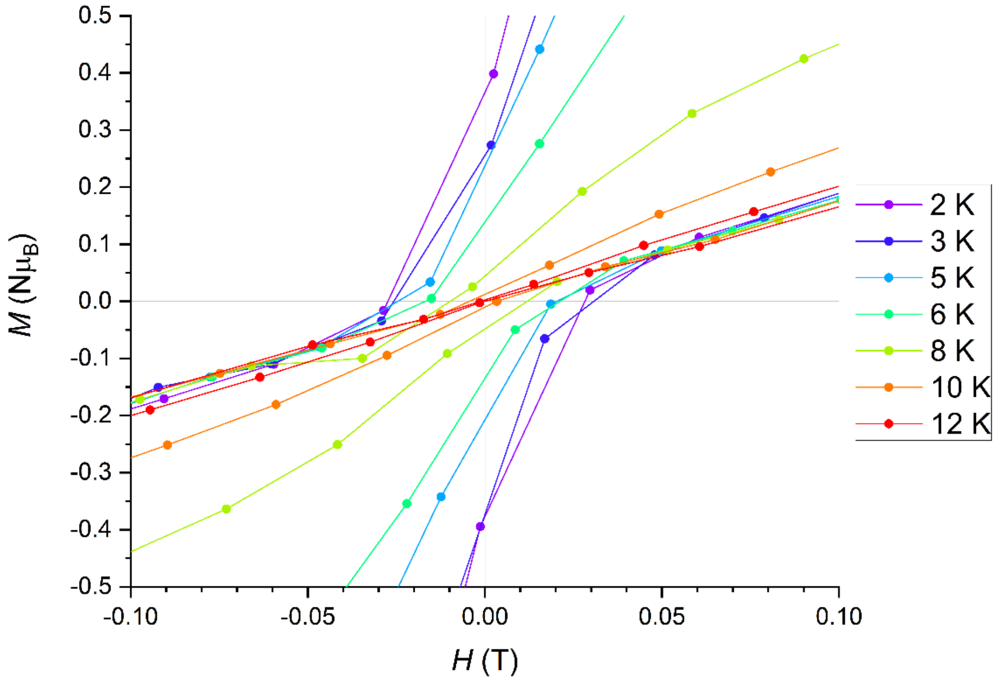

Figure S67. Hysteresis loops of **4-Dy** from 2 to 12 K zoomed to –0.1 T to +0.1 T. Sweep rate is 22 Oe s^{-1}.

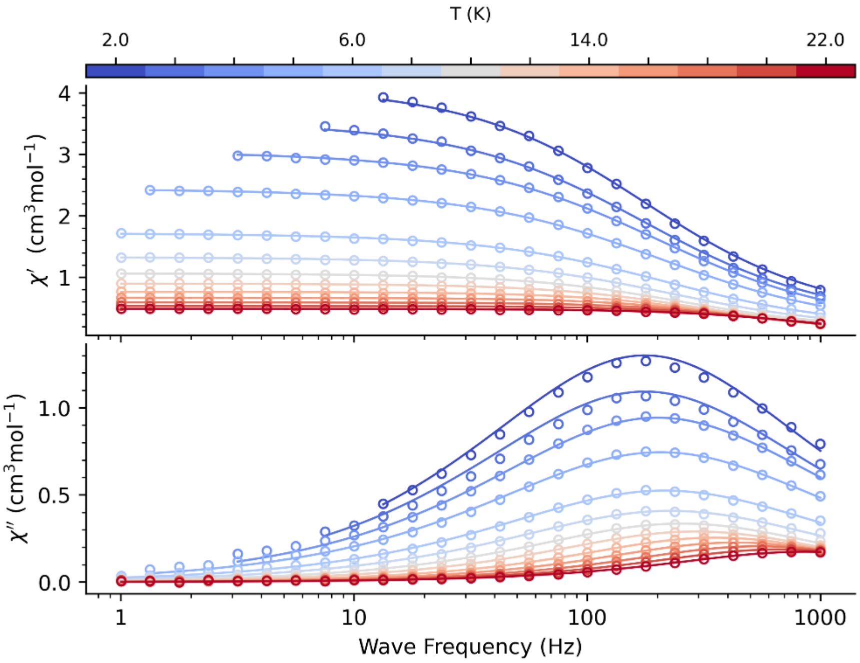

Figure S68. In-phase (top) and out-of-phase (bottom) ac susceptibilities of "[{Dy(Cp*)$_2$}{Al[OC(CF_3)$_3$]$_4$}]" in a zero field. Solid lines are fits to the generalized Debye model in CC-FIT2,[22,23] giving $0.122 \leq \alpha \leq 0.264$.

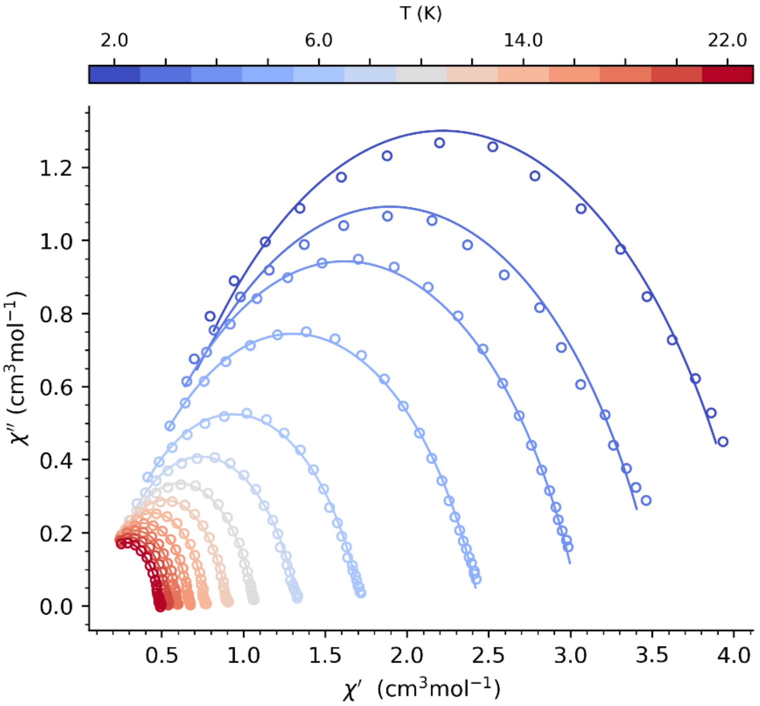

Figure S69. Cole-Cole plot showing fitting of ac data for "[{Dy(Cp*)$_2$}{Al[OC(CF_3)$_3$]$_4$}]" in a zero dc field. Solid lines are fits to the generalized Debye model in CC-FIT2,[22,23] giving $0.122 \leq \alpha \leq 0.264$.

Table S15. Best fit parameters to the generalized Debye model for "[{Dy(Cp*)$_2$}{Al[OC(CF$_3$)$_3$]$_4$}]" in zero dc field.

T	τ	τ^{err}	χ_S	χ_S^{err}	χ_T	χ_T^{err}	α	α^{err}	$\langle\ln\tau\rangle$	$\sigma_{\langle\ln\tau\rangle}$
(K)	(s)		(emu/mol)		(emu/mol)				ln (s)	
2.00	9.00E-4	1.57E-5	2.99E-1	3.25E-2	4.13	2.26E-2	2.41E-1	8.84E-3	-7.01	1.56
2.50	9.01E-4	1.90E-5	2.48E-1	3.27E-2	3.54	1.87E-2	2.55E-1	9.60E-3	-7.01	1.62
3.00	7.94E-4	1.02E-5	1.81E-1	1.69E-2	3.05	6.96E-3	2.60E-1	5.04E-3	-7.14	1.65
4.00	7.73E-4	9.75E-6	1.60E-1	1.29E-2	2.44	4.34E-3	2.64E-1	4.55E-3	-7.17	1.67
6.00	7.42E-4	9.08E-6	1.50E-1	8.80E-3	1.72	2.75E-3	2.49E-1	4.45E-3	-7.21	1.60
8.00	6.99E-4	8.46E-6	1.49E-1	6.79E-3	1.33	2.07E-3	2.29E-1	4.58E-3	-7.27	1.50
10.00	6.26E-4	7.44E-6	1.44E-1	5.53E-3	1.06	1.59E-3	1.98E-1	4.74E-3	-7.38	1.35
12.00	5.46E-4	7.13E-6	1.35E-1	5.34E-3	9.00E-1	1.41E-3	1.74E-1	5.35E-3	-7.51	1.24
14.00	4.48E-4	5.64E-6	1.23E-1	4.62E-3	7.65E-1	1.07E-3	1.46E-1	5.21E-3	-7.71	1.10
16.00	3.68E-4	5.93E-6	1.10E-1	5.37E-3	6.68E-1	1.06E-3	1.31E-1	6.45E-3	-7.91	1.03
18.00	2.94E-4	4.53E-6	9.34E-2	4.75E-3	5.94E-1	7.61E-4	1.24E-1	5.71E-3	-8.13	1.00
20.00	2.37E-4	6.15E-6	8.06E-2	7.34E-3	5.35E-1	9.42E-4	1.22E-1	8.66E-3	-8.35	0.99
22.00	1.87E-4	6.68E-6	5.88E-2	9.44E-3	4.87E-1	9.13E-4	1.32E-1	1.02E-2	-8.59	1.04

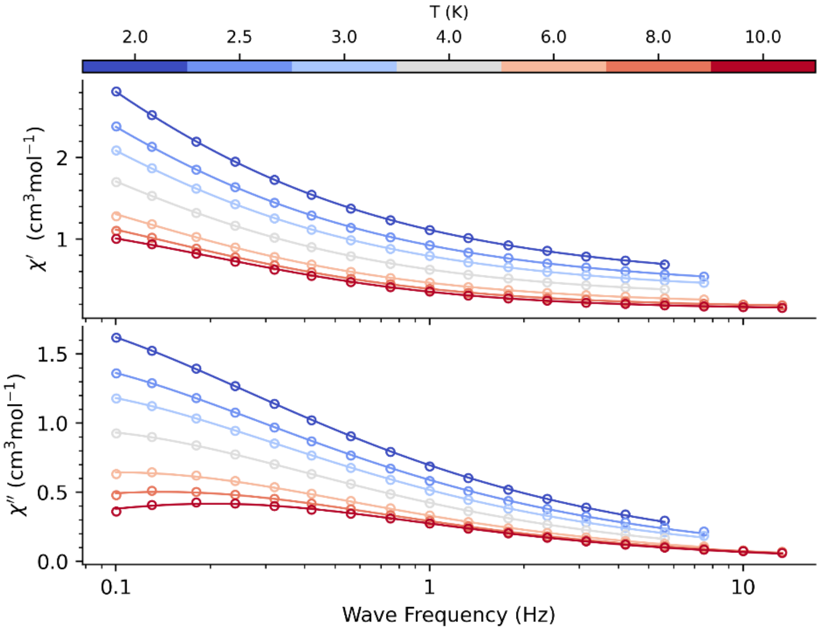

Figure S70. In-phase (top) and out-of-phase (bottom) ac susceptibilities of **2-Dy** in a zero field from 2–10 K. Solid lines are fits to the generalized Debye model in CC-FIT2,[22,23] giving 0.277 ≤ α ≤ 0.418.

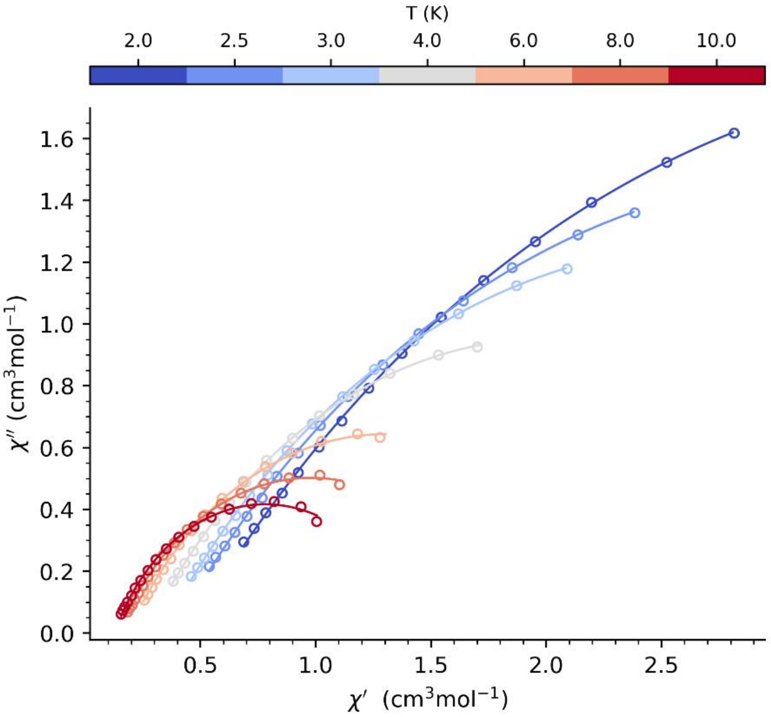

Figure S71. Cole-Cole plot showing fitting of ac data for **2-Dy** in a zero dc field from 2–10 K. Solid lines are fits to the generalized Debye model in CC-FIT2,[22,23] giving 0.277 ≤ α ≤ 0.418.

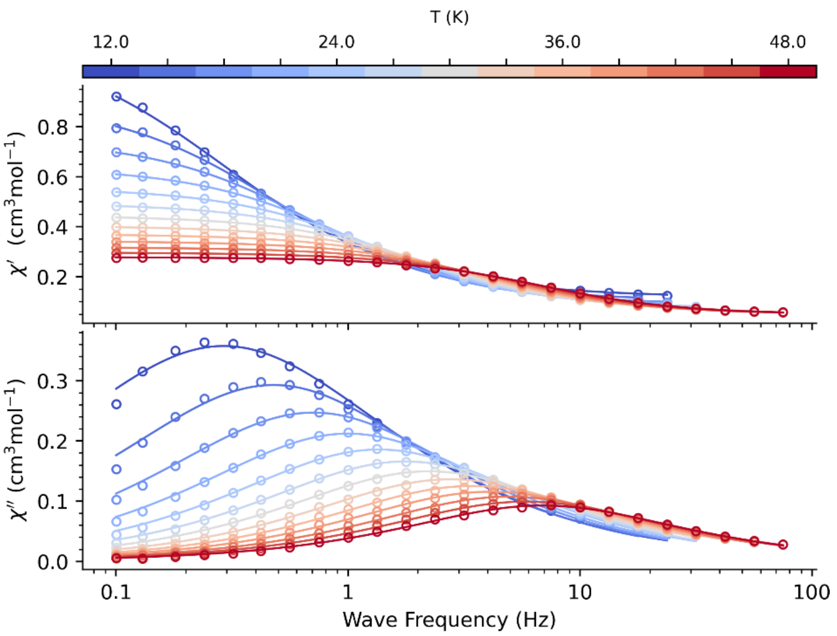

Figure S72. In-phase (top) and out-of-phase (bottom) ac susceptibilities of **2-Dy** in a zero field from 12–48 K. Solid lines are fits to the generalized Debye model in CC-FIT2,[22,23] giving 0.138 ≤ α ≤ 0.247.

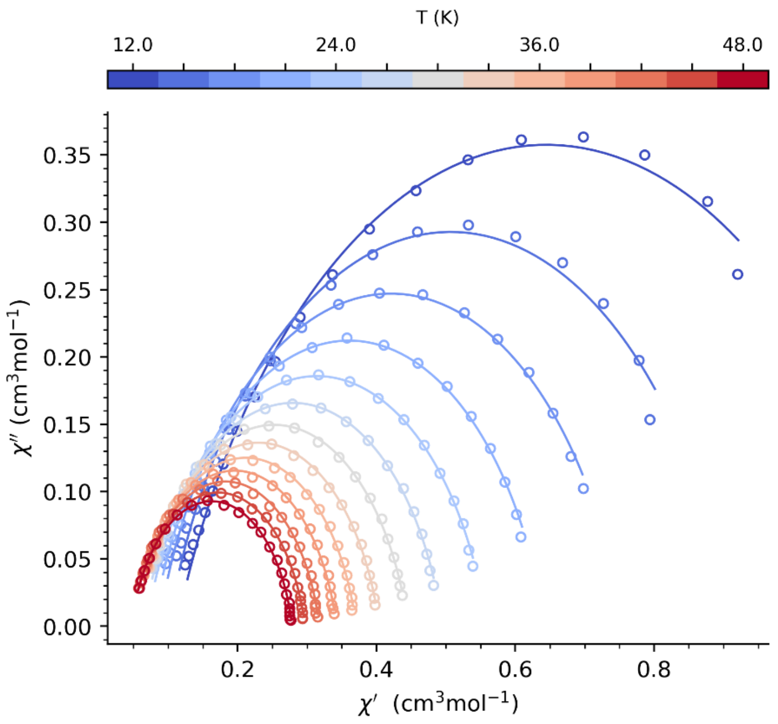

Figure S73. Cole-Cole plot showing fitting of ac data for **2-Dy** in a zero dc field from 12–48 K. Solid lines are fits to the generalized Debye model in CC-FIT2,[22,23] giving 0.138 ≤ α ≤ 0.247.

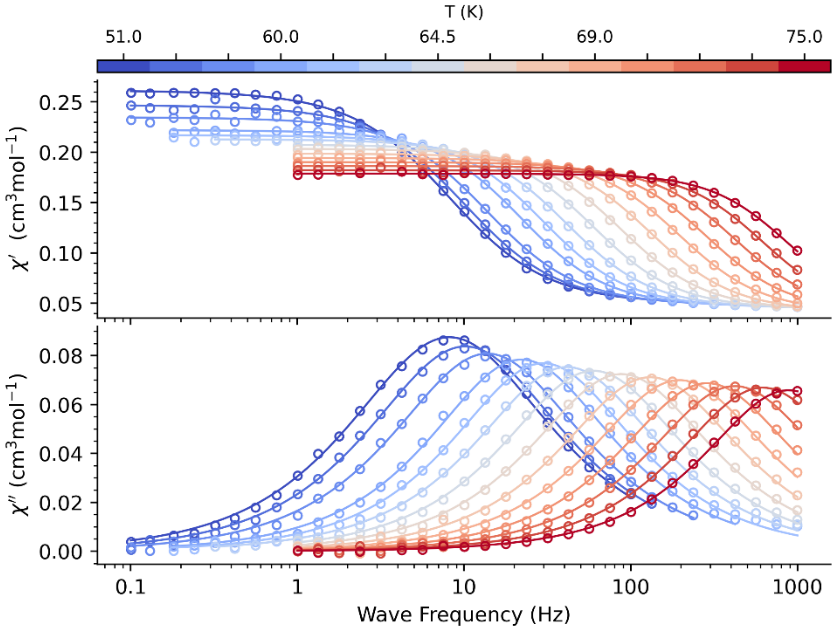

Figure S74. In-phase (top) and out-of-phase (bottom) ac susceptibilities of **2-Dy** in a zero field from 51–75 K. Solid lines are fits to the generalized Debye model in CC-FIT2,[22,23] giving 0.034 ≤ α ≤ 0.125.

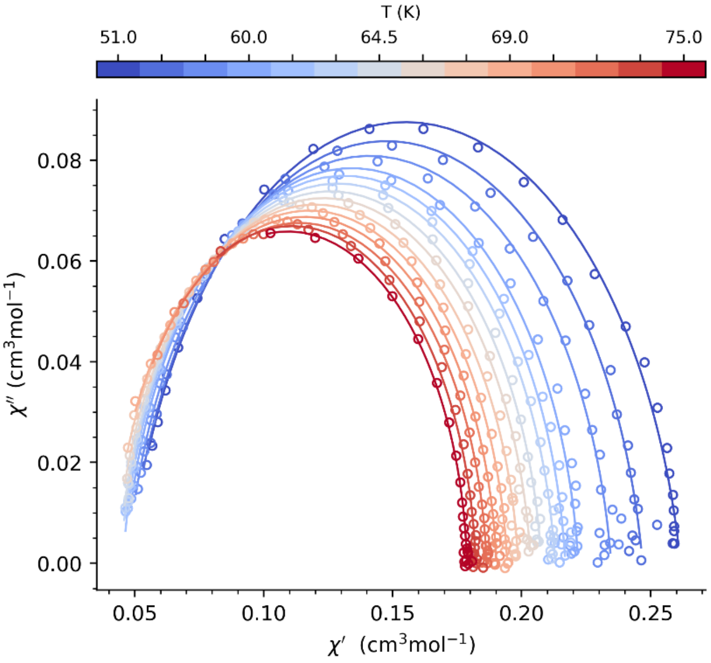

Figure S75. Cole-Cole plot showing fitting of ac data for **2-Dy** in a zero dc field from 51–75 K. Solid lines are fits to the generalized Debye model in CC-FIT2,[22,23] giving 0.034 ≤ α ≤

Table S16. Best fit parameters to the generalized Debye model for **2-Dy** in zero dc field.

T	τ	τ^{err}	χ_S	χ_S^{err}	χ_T	χ_T^{err}	α	α^{err}	$\langle \ln \tau \rangle$	$\sigma_{\langle \ln \tau \rangle}$
(K)	(s)		(emu/mol)		(emu/mol)				ln (s)	
2.00	4.53	1.08E-1	4.52E-1	3.83E-3	7.82	6.60E-2	4.18E-1	2.23E-3	1.51	2.53
2.50	3.85	1.11E-1	3.79E-1	3.99E-3	6.24	6.51E-2	4.03E-1	2.92E-3	1.35	2.44
3.00	3.23	7.14E-2	3.30E-1	2.95E-3	5.13	4.19E-2	3.88E-1	2.51E-3	1.17	2.34
4.00	2.41	4.22E-2	2.65E-1	2.47E-3	3.74	2.51E-2	3.62E-1	2.50E-3	0.88	2.19
6.00	1.58	5.54E-2	1.87E-1	4.61E-3	2.42	3.38E-2	3.34E-1	6.45E-3	0.46	2.03
8.00	1.13	2.21E-2	1.47E-1	2.42E-3	1.81	1.47E-2	3.10E-1	4.34E-3	0.12	1.90
10.00	7.82E-1	1.57E-2	1.27E-1	2.93E-3	1.43	1.26E-2	2.77E-1	5.81E-3	-0.25	1.73
12.00	5.47E-1	1.19E-2	1.12E-1	3.24E-3	1.18	1.14E-2	2.47E-1	7.53E-3	-0.60	1.59
15.00	3.34E-1	6.11E-3	9.98E-2	3.51E-3	9.14E-1	7.53E-3	2.06E-1	8.49E-3	-1.10	1.39
18.00	2.24E-1	2.67E-3	8.63E-2	2.22E-3	7.56E-1	3.78E-3	1.91E-1	5.98E-3	-1.49	1.32
21.00	1.59E-1	1.60E-3	7.86E-2	1.87E-3	6.43E-1	2.50E-3	1.79E-1	5.34E-3	-1.84	1.26
24.00	1.17E-1	1.09E-3	6.94E-2	1.57E-3	5.59E-1	1.86E-3	1.74E-1	4.97E-3	-2.14	1.24
27.00	9.00E-2	7.03E-4	6.46E-2	1.35E-3	4.95E-1	1.26E-3	1.64E-1	4.27E-3	-2.41	1.19
30.00	7.15E-2	4.37E-4	6.02E-2	1.04E-3	4.46E-1	8.14E-4	1.60E-1	3.36E-3	-2.64	1.17
33.00	5.74E-2	4.00E-4	5.57E-2	1.06E-3	4.04E-1	8.03E-4	1.55E-1	3.81E-3	-2.86	1.15
36.00	4.67E-2	3.70E-4	5.25E-2	1.16E-3	3.71E-1	7.75E-4	1.52E-1	4.29E-3	-3.06	1.13
39.00	3.89E-2	2.77E-4	4.95E-2	9.37E-4	3.43E-1	6.23E-4	1.50E-1	3.82E-3	-3.25	1.12
42.00	3.21E-2	2.28E-4	4.75E-2	8.45E-4	3.18E-1	5.60E-4	1.45E-1	3.80E-3	-3.44	1.10
45.00	2.71E-2	2.05E-4	4.67E-2	8.12E-4	2.97E-1	5.41E-4	1.46E-1	4.02E-3	-3.61	1.11
48.00	2.31E-2	1.82E-4	4.76E-2	8.14E-4	2.79E-1	4.98E-4	1.38E-1	4.19E-3	-3.77	1.07
51.00	1.95E-2	1.55E-4	4.83E-2	7.53E-4	2.62E-1	4.68E-4	1.25E-1	4.33E-3	-3.94	1.00
54.00	1.56E-2	1.59E-4	4.84E-2	8.91E-4	2.47E-1	5.51E-4	1.07E-1	5.61E-3	-4.16	0.92
57.00	1.15E-2	1.21E-4	4.74E-2	8.31E-4	2.35E-1	5.37E-4	9.33E-2	5.87E-3	-4.46	0.84
60.00	7.20E-3	6.31E-5	4.65E-2	6.50E-4	2.22E-1	4.44E-4	7.14E-2	5.08E-3	-4.93	0.72

61.50	5.32E-3	5.09E-5	4.55E-2	6.21E-4	2.17E-1	4.68E-4	6.89E-2	5.46E-3	-5.24	0.71
63.00	3.84E-3	2.10E-5	4.46E-2	3.71E-4	2.13E-1	2.68E-4	7.04E-2	3.16E-3	-5.56	0.72
64.50	2.66E-3	1.56E-5	4.40E-2	4.19E-4	2.08E-1	3.13E-4	6.53E-2	3.52E-3	-5.93	0.69
66.00	1.81E-3	1.03E-5	4.30E-2	4.38E-4	2.04E-1	2.71E-4	6.51E-2	3.39E-3	-6.31	0.69
67.50	1.21E-3	8.50E-6	4.17E-2	5.86E-4	1.99E-1	2.93E-4	6.20E-2	4.15E-3	-6.72	0.67
69.00	8.13E-4	6.35E-6	4.10E-2	7.08E-4	1.94E-1	2.91E-4	5.84E-2	4.55E-3	-7.11	0.65
70.50	5.53E-4	4.39E-6	4.17E-2	7.69E-4	1.90E-1	2.43E-4	4.90E-2	4.45E-3	-7.50	0.59
72.00	3.78E-4	3.32E-6	4.12E-2	8.90E-4	1.86E-1	1.98E-4	4.59E-2	4.44E-3	-7.88	0.57
73.50	2.57E-4	3.90E-6	3.99E-2	1.58E-3	1.82E-1	2.38E-4	3.90E-2	6.57E-3	-8.27	0.52
75.00	1.80E-4	2.72E-6	4.00E-2	1.56E-3	1.79E-1	1.63E-4	3.35E-2	5.40E-3	-8.62	0.48

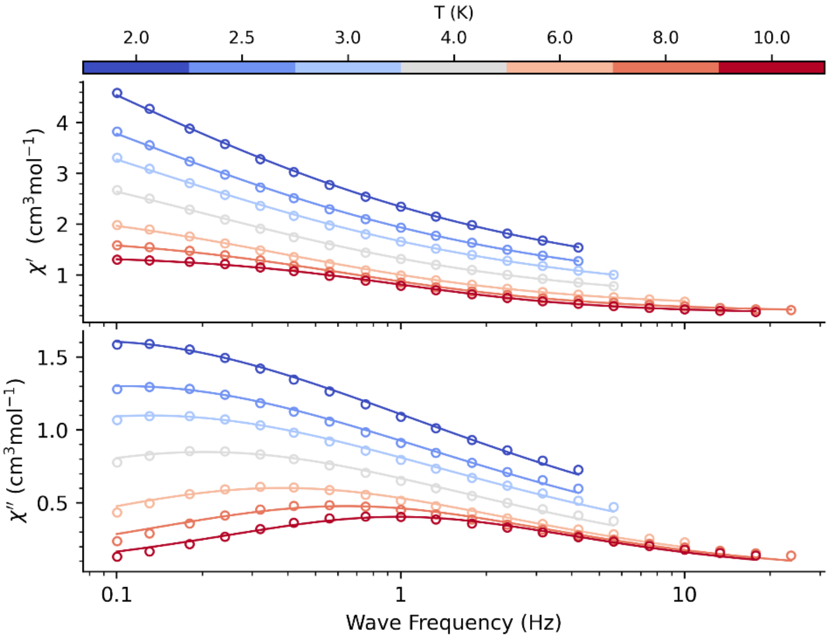

Figure S76. In-phase (top) and out-of-phase (bottom) ac susceptibilities of **3-Dy** in a zero field from 2–10 K. Solid lines are fits to the generalized Debye model in CC-FIT2,[22,23] giving 0.232 ≤ α ≤ 0.529.

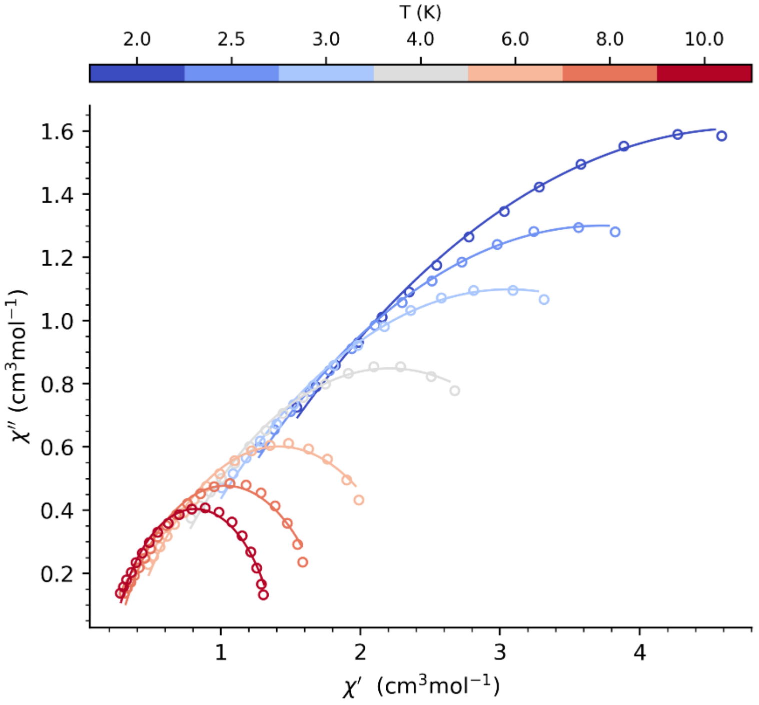

Figure S77. Cole-Cole plot showing fitting of ac data for **3-Dy** in a zero dc field from 2–10 K. Solid lines are fits to the generalized Debye model in CC-FIT2,[22,23] giving 0.232 ≤ α ≤ 0.529.

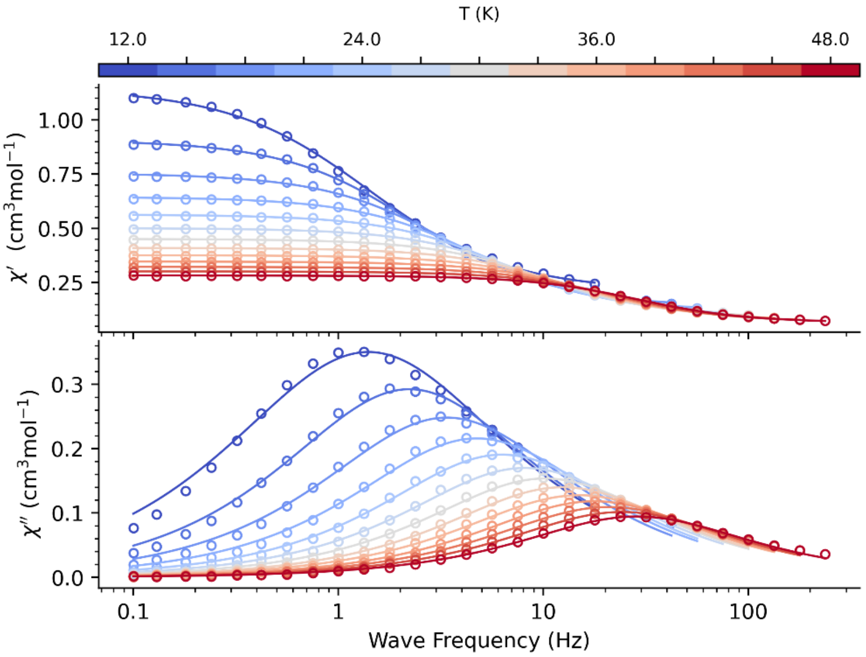

Figure S78. In-phase (top) and out-of-phase (bottom) ac susceptibilities of **3-Dy** in a zero field from 12–48 K. Solid lines are fits to the generalized Debye model in CC-FIT2,[22,23] giving $0.0927 \leq \alpha \leq 0.191$.

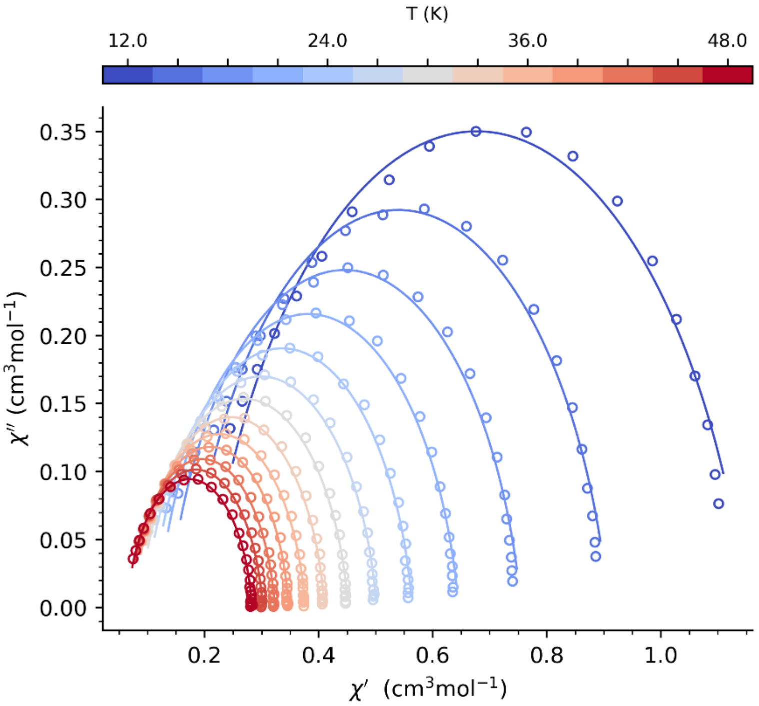

Figure S79. Cole-Cole plot showing fitting of ac data for **3-Dy** in a zero dc field from 12–48 K. Solid lines are fits to the generalized Debye model in CC-FIT2,[22,23] giving $0.0927 \leq \alpha \leq 0.191$.

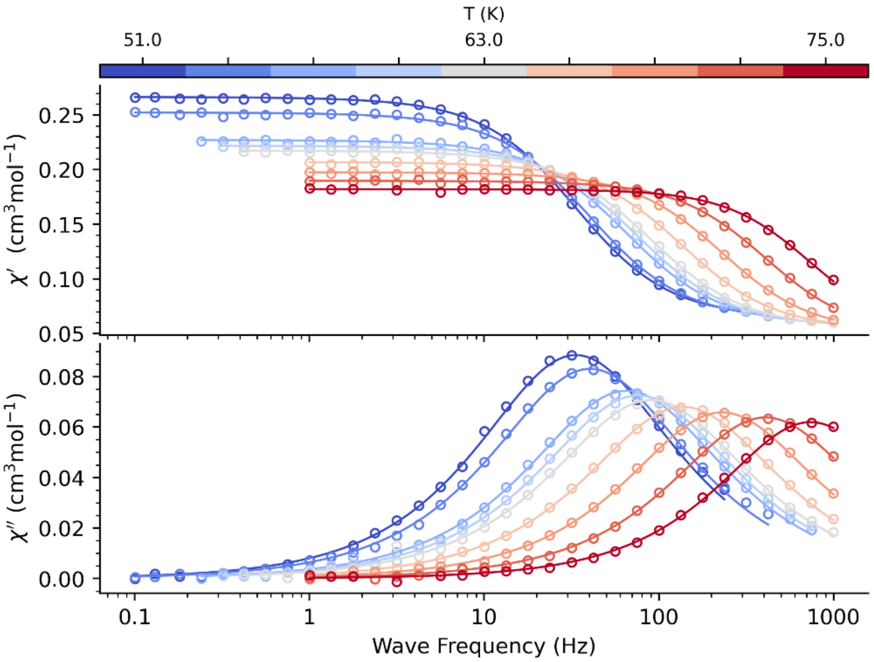

Figure S80. In-phase (top) and out-of-phase (bottom) ac susceptibilities of **3-Dy** in a zero field from 51–75 K. Solid lines are fits to the generalized Debye model in CC-FIT2,[22,23] giving $0.0492 \leq \alpha \leq 0.0871$.

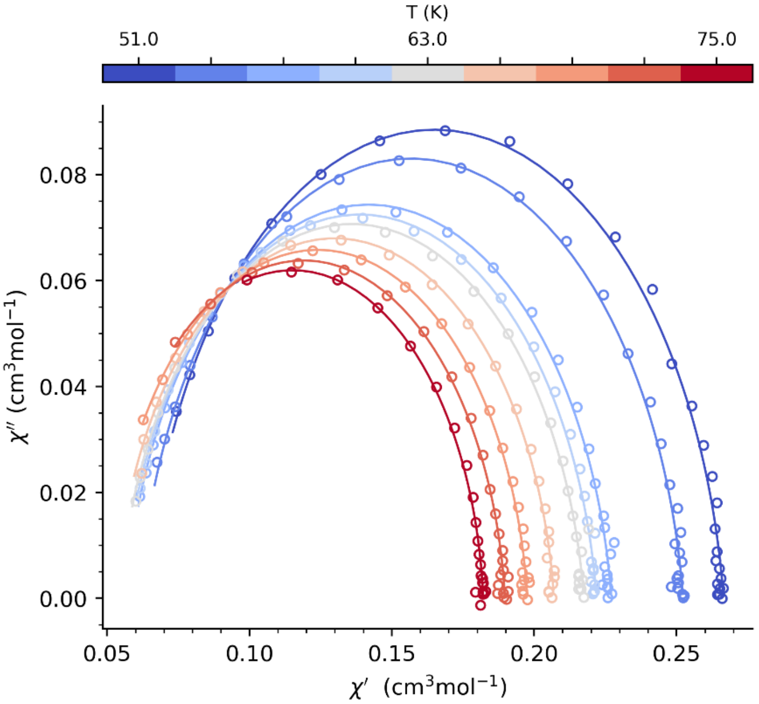

Figure S81. Cole-Cole plot showing fitting of ac data for **3-Dy** in a zero dc field from 51–75 K. Solid lines are fits to the generalized Debye model in CC-FIT2,[22,23] giving $0.0492 \leq \alpha \leq 0.0871$.

Table S17. Best fit parameters to the generalized Debye model for **3-Dy** in zero dc field.

T	τ	τ^{err}	χ_S	χ_S^{err}	χ_T	χ_T^{err}	α	α^{err}	$\langle\ln\tau\rangle$	$\sigma_{\langle\ln\tau\rangle}$
(K)	(s)		(emu/mol)		(emu/mol)				ln (s)	
2.00	1.97	1.69E-1	6.29E-1	4.05E-2	8.93	2.17E-1	5.29E-1	9.77E-3	0.68	3.40
2.50	1.51	1.11E-1	5.71E-1	3.36E-2	6.89	1.52E-1	5.03E-1	1.02E-2	0.41	3.17
3.00	1.22	8.27E-2	4.98E-1	2.76E-2	5.63	1.18E-1	4.85E-1	1.04E-2	0.20	3.02
4.00	7.82E-1	3.91E-2	4.42E-1	2.23E-2	3.98	6.90E-2	4.30E-1	1.13E-2	-0.25	2.62
6.00	4.16E-1	1.52E-2	3.35E-1	1.55E-2	2.50	3.45E-2	3.53E-1	1.22E-2	-0.88	2.14
8.00	2.48E-1	7.74E-3	2.57E-1	1.11E-2	1.81	2.10E-2	2.99E-1	1.24E-2	-1.40	1.85
10.00	1.63E-1	3.94E-3	2.31E-1	9.58E-3	1.40	1.20E-2	2.32E-1	1.21E-2	-1.82	1.51
12.00	1.13E-1	2.38E-3	2.02E-1	8.02E-3	1.15	7.74E-3	1.91E-1	1.13E-2	-2.18	1.32
15.00	7.17E-2	1.25E-3	1.72E-1	6.19E-3	9.09E-1	4.36E-3	1.47E-1	9.82E-3	-2.64	1.11
18.00	4.70E-2	8.05E-4	1.36E-1	4.58E-3	7.55E-1	3.43E-3	1.40E-1	9.44E-3	-3.06	1.08
21.00	3.36E-2	5.25E-4	1.18E-1	3.66E-3	6.46E-1	2.51E-3	1.27E-1	8.66E-3	-3.39	1.01
24.00	2.52E-2	3.39E-4	1.08E-1	2.96E-3	5.64E-1	1.73E-3	1.14E-1	7.49E-3	-3.68	0.95
27.00	1.96E-2	2.52E-4	9.60E-2	2.51E-3	5.02E-1	1.42E-3	1.12E-1	7.14E-3	-3.93	0.94
30.00	1.56E-2	2.02E-4	8.75E-2	2.24E-3	4.52E-1	1.24E-3	1.08E-1	7.15E-3	-4.16	0.92
33.00	1.28E-2	1.53E-4	8.28E-2	1.97E-3	4.10E-1	9.88E-4	9.95E-2	6.65E-3	-4.36	0.88
36.00	1.06E-2	1.21E-4	7.59E-2	1.68E-3	3.77E-1	8.49E-4	1.05E-1	6.27E-3	-4.54	0.90
39.00	8.97E-3	9.40E-5	7.34E-2	1.47E-3	3.47E-1	6.85E-4	9.40E-2	5.80E-3	-4.71	0.85

42.00	7.63E-3	8.74E-5	6.88E-2	1.45E-3	3.23E-1	6.90E-4	9.66E-2	6.30E-3	-4.88	0.86
45.00	6.57E-3	6.58E-5	6.69E-2	1.22E-3	3.02E-1	5.37E-4	9.27E-2	5.51E-3	-5.03	0.84
48.00	5.57E-3	5.16E-5	6.45E-2	1.02E-3	2.83E-1	4.61E-4	9.29E-2	5.09E-3	-5.19	0.84
51.00	4.75E-3	3.65E-5	6.35E-2	8.17E-4	2.67E-1	3.42E-4	8.71E-2	4.22E-3	-5.35	0.81
54.00	3.93E-3	3.08E-5	6.12E-2	7.14E-4	2.52E-1	3.41E-4	8.90E-2	4.32E-3	-5.54	0.82
60.00	2.44E-3	1.71E-5	5.67E-2	5.59E-4	2.27E-1	2.90E-4	8.65E-2	3.92E-3	-6.02	0.81
61.50	2.09E-3	1.18E-5	5.56E-2	4.54E-4	2.22E-1	2.27E-4	8.72E-2	3.15E-3	-6.17	0.81
63.00	1.76E-3	1.44E-5	5.44E-2	6.26E-4	2.18E-1	3.30E-4	9.06E-2	4.56E-3	-6.34	0.83
66.00	1.16E-3	8.09E-6	5.29E-2	5.68E-4	2.07E-1	2.73E-4	7.95E-2	4.00E-3	-6.76	0.77
69.00	6.93E-4	5.23E-6	5.04E-2	6.68E-4	1.98E-1	2.34E-4	7.11E-2	4.16E-3	-7.28	0.72
72.00	3.90E-4	3.92E-6	4.83E-2	9.52E-4	1.90E-1	2.21E-4	6.54E-2	4.91E-3	-7.85	0.69
75.00	2.16E-4	3.64E-6	4.82E-2	1.63E-3	1.82E-1	2.12E-4	4.92E-2	6.52E-3	-8.44	0.59

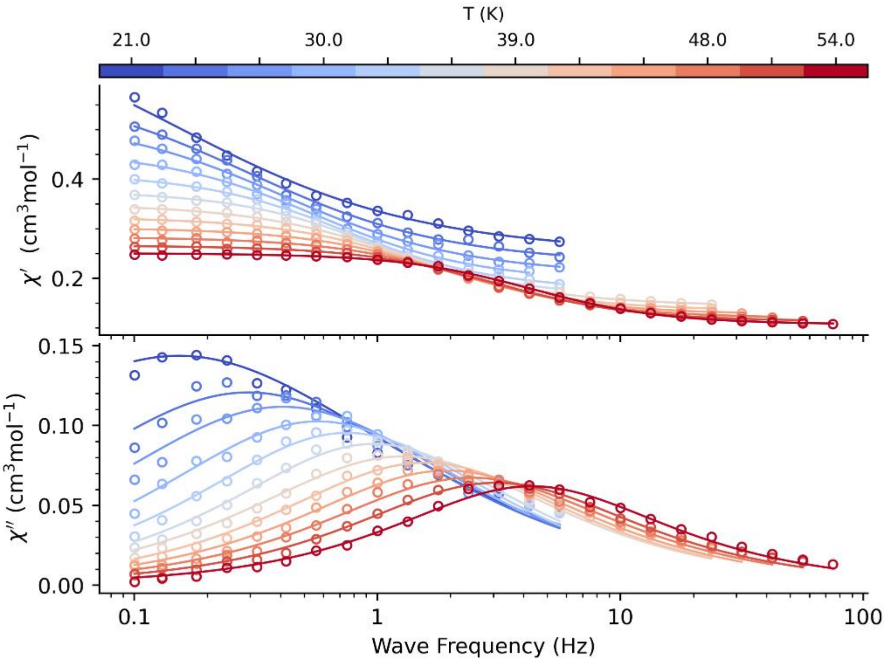

Figure S82. In-phase (top) and out-of-phase (bottom) ac susceptibilities of **4-Dy** in a zero field from 21–54 K. Solid lines are fits to the generalized Debye model in CC-FIT2,[22,23] giving $0.0978 \leq \alpha \leq 0.351$.

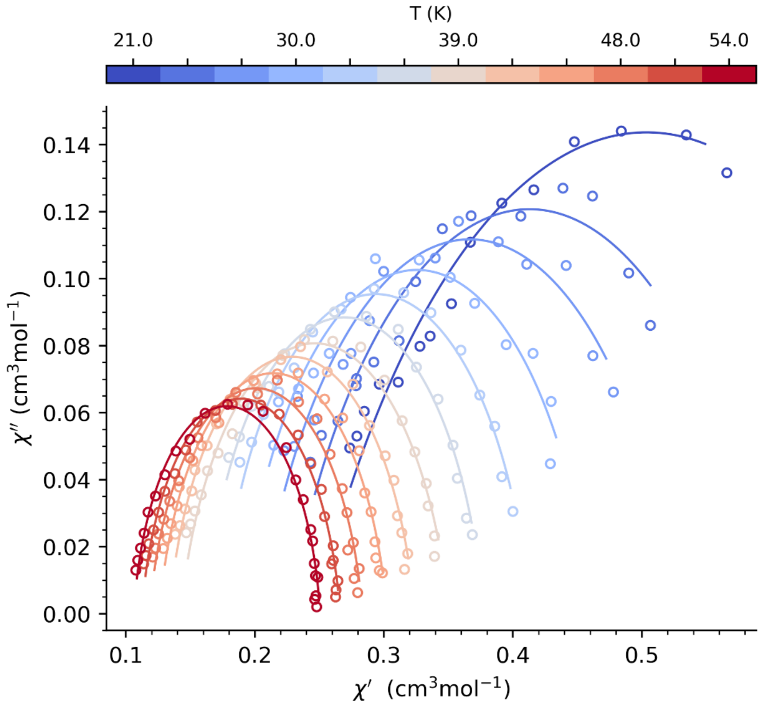

Figure S83. Cole-Cole plot showing fitting of ac data for **4-Dy** in a zero dc field from 21–54 K. Solid lines are fits to the generalized Debye model in CC-FIT2,[22,23] giving $0.0978 \leq \alpha \leq 0.351$.

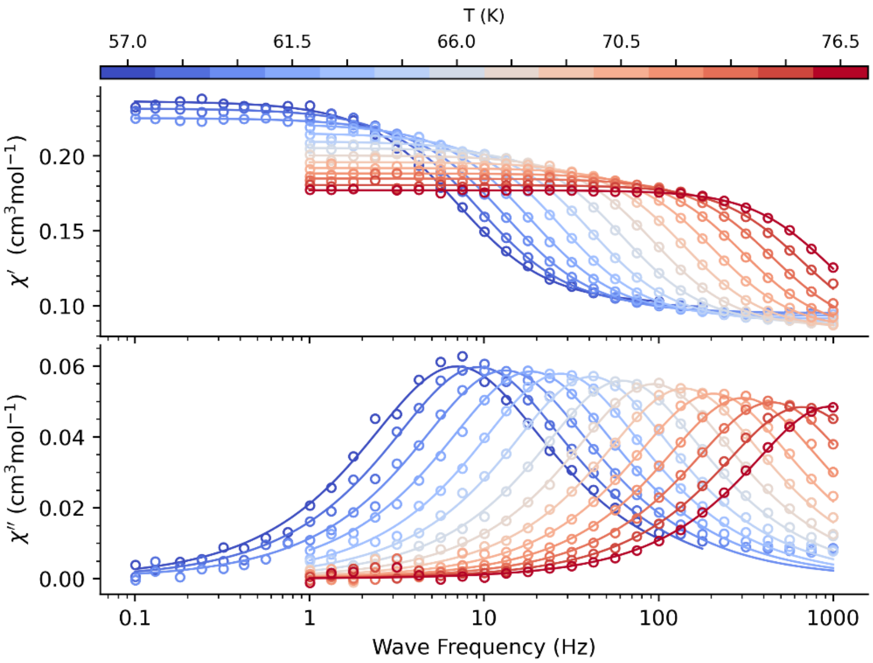

Figure S84. In-phase (top) and out-of-phase (bottom) ac susceptibilities of **4-Dy** in a zero field from 57–76.5 K. Solid lines are fits to the generalized Debye model in CC-FIT2,[22,23] giving $0.0231 \leq \alpha \leq 0.0803$.

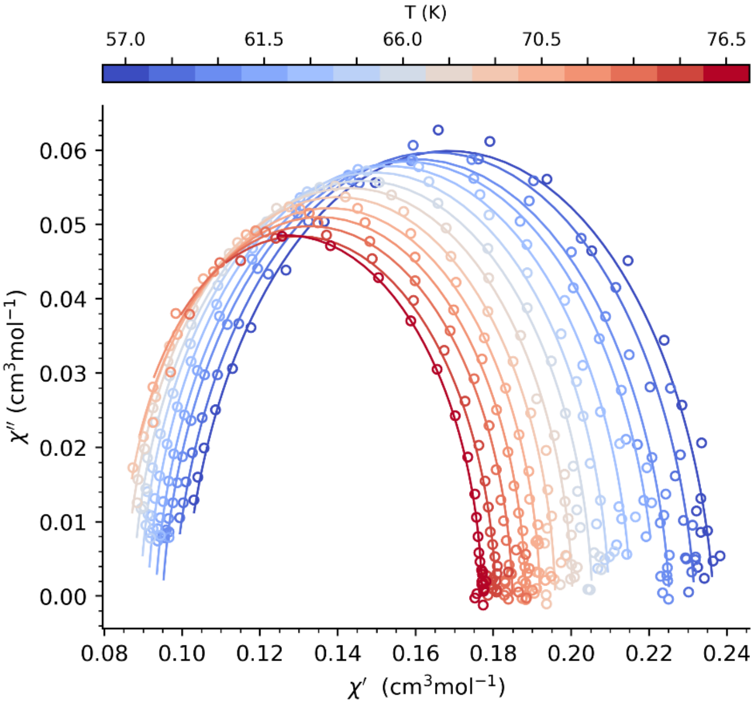

Figure S85. Cole-Cole plot showing fitting of ac data for **4-Dy** in a zero dc field from 57–76.5 K. Solid lines are fits to the generalized Debye model in CC-FIT2,[22,23] giving $0.0231 \leq \alpha \leq 0.0803$.

Table S18. Best fit parameters to the generalized Debye model for **4-Dy** in zero dc field.

T	τ	τ^{err}	χ_S	χ_S^{err}	χ_T	χ_T^{err}	α	α^{err}	$\langle\ln\tau\rangle$	$\sigma_{\langle\ln\tau\rangle}$
(K)	(s)		(emu/mol)		(emu/mol)				ln (s)	
21.00	1.04	1.39E-1	2.47E-1	6.80E-3	7.60E-1	2.96E-2	3.51E-1	3.09E-2	0.04	2.13
24.00	5.40E-1	3.66E-2	2.26E-1	5.47E-3	6.00E-1	1.29E-2	2.70E-1	2.70E-2	-0.62	1.70
27.00	3.89E-1	2.07E-2	2.03E-1	5.24E-3	5.30E-1	9.05E-3	2.36E-1	2.61E-2	-0.94	1.53
30.00	2.79E-1	9.79E-3	1.90E-1	4.30E-3	4.62E-1	4.86E-3	1.77E-1	2.11E-2	-1.28	1.25
33.00	2.12E-1	4.98E-3	1.74E-1	2.69E-3	4.14E-1	2.69E-3	1.45E-1	1.46E-2	-1.55	1.10
36.00	1.65E-1	3.49E-3	1.61E-1	2.44E-3	3.77E-1	1.97E-3	1.26E-1	1.33E-2	-1.80	1.01
39.00	1.24E-1	2.93E-3	1.42E-1	1.81E-3	3.51E-1	2.05E-3	1.60E-1	1.30E-2	-2.08	1.17
42.00	1.01E-1	2.01E-3	1.34E-1	1.53E-3	3.24E-1	1.51E-3	1.37E-1	1.14E-2	-2.29	1.06
45.00	8.22E-2	1.62E-3	1.26E-1	1.39E-3	3.03E-1	1.32E-3	1.31E-1	1.12E-2	-2.50	1.03
48.00	6.54E-2	1.23E-3	1.18E-1	1.25E-3	2.84E-1	1.13E-3	1.30E-1	1.06E-2	-2.73	1.03
51.00	5.15E-2	8.59E-4	1.12E-1	1.03E-3	2.66E-1	8.95E-4	1.15E-1	9.48E-3	-2.97	0.95
54.00	3.69E-2	6.00E-4	1.06E-1	9.76E-4	2.51E-1	7.78E-4	9.78E-2	9.36E-3	-3.30	0.87
57.00	2.29E-2	3.18E-4	1.01E-1	8.40E-4	2.37E-1	5.79E-4	8.03E-2	8.12E-3	-3.78	0.77
58.50	1.71E-2	1.87E-4	9.79E-2	6.16E-4	2.32E-1	4.41E-4	7.23E-2	6.37E-3	-4.07	0.73
60.00	1.21E-2	1.50E-4	9.50E-2	5.34E-4	2.25E-1	4.90E-4	6.54E-2	7.05E-3	-4.42	0.69
61.50	8.57E-3	1.01E-4	9.32E-2	5.09E-4	2.22E-1	6.48E-4	5.98E-2	7.12E-3	-4.76	0.66
63.00	5.78E-3	6.33E-5	9.12E-2	5.07E-4	2.15E-1	5.39E-4	4.44E-2	6.75E-3	-5.15	0.56
64.50	3.87E-3	3.72E-5	8.93E-2	4.78E-4	2.10E-1	4.26E-4	3.48E-2	6.00E-3	-5.55	0.49

66.00	2.57E-3	2.59E-5	8.74E-2	5.39E-4	2.05E-1	4.00E-4	3.47E-2	6.28E-3	-5.96	0.49
67.50	1.71E-3	1.16E-5	8.56E-2	3.96E-4	2.00E-1	2.42E-4	2.82E-2	4.28E-3	-6.37	0.44
69.00	1.14E-3	1.29E-5	8.42E-2	7.16E-4	1.96E-1	3.70E-4	2.53E-2	7.17E-3	-6.78	0.42
70.50	7.74E-4	9.75E-6	8.37E-2	8.54E-4	1.92E-1	3.38E-4	2.31E-2	7.76E-3	-7.16	0.40
72.00	5.24E-4	6.27E-6	8.25E-2	8.67E-4	1.88E-1	2.60E-4	2.38E-2	6.96E-3	-7.55	0.40
73.50	3.58E-4	7.55E-6	8.08E-2	1.59E-3	1.85E-1	3.43E-4	2.87E-2	1.09E-2	-7.93	0.44
75.00	2.41E-4	6.64E-6	7.92E-2	2.08E-3	1.81E-1	2.96E-4	2.95E-2	1.18E-2	-8.33	0.45
76.50	1.66E-4	4.84E-6	7.63E-2	2.23E-3	1.77E-1	2.03E-4	2.38E-2	1.00E-2	-8.70	0.40

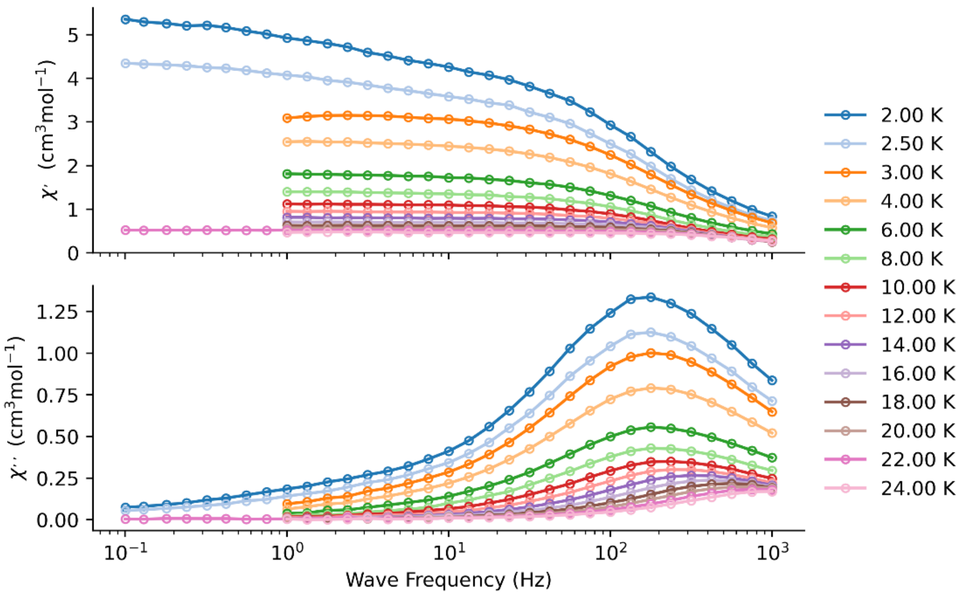

Figure S86. Unedited in-phase (top) and out-of-phase (bottom) ac susceptibilities of "[{Dy(Cp*)$_2$}{Al[OC(CF$_3$)$_3$]$_4$}]" in a zero field.

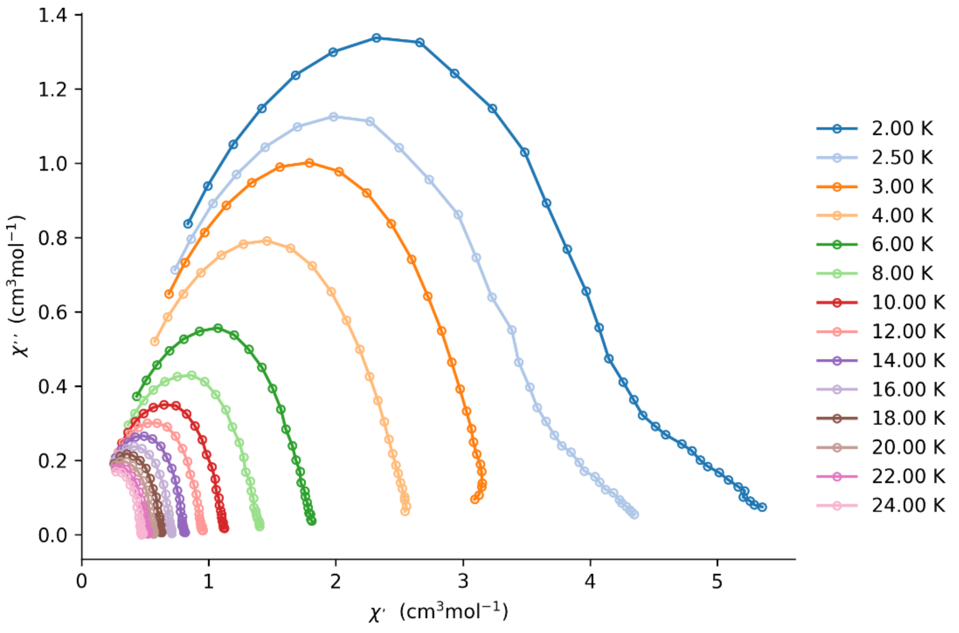

Figure S87. Unedited Cole-Cole plot showing ac data for "[{Dy(Cp*)$_2$}{Al[OC(CF$_3$)$_3$]$_4$}]" in a zero dc field.

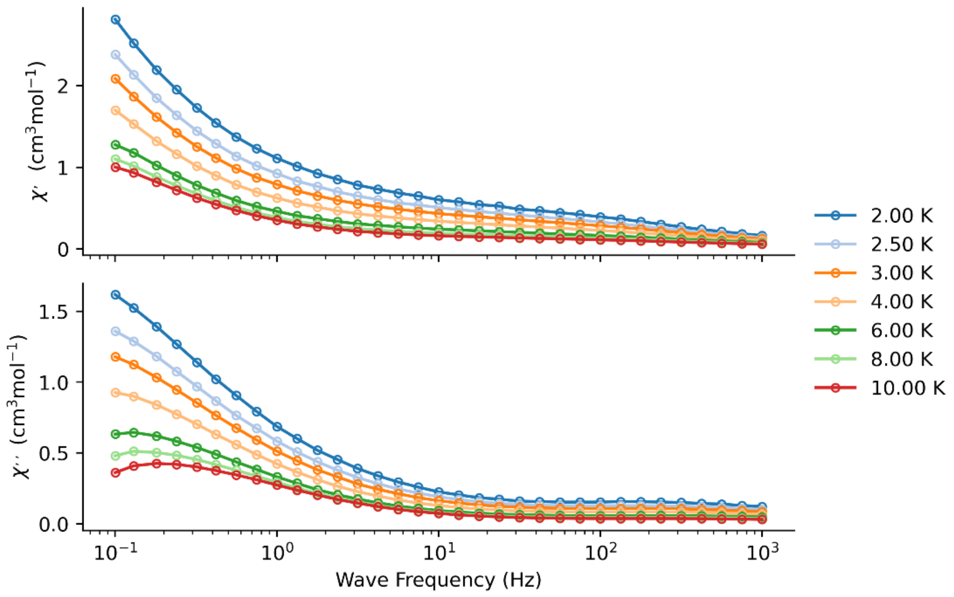

Figure S88. Unedited in-phase (top) and out-of-phase (bottom) ac susceptibilities of **2-Dy** in a zero field from 2–10 K.

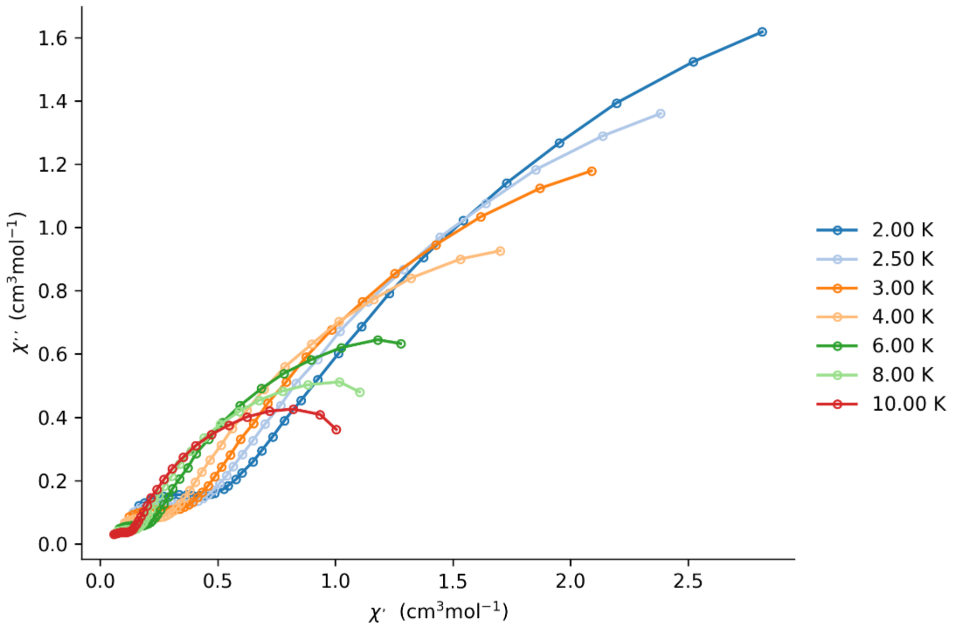

Figure S89. Unedited Cole-Cole plot showing ac data for **2-Dy** in a zero dc field from 2–10 K.

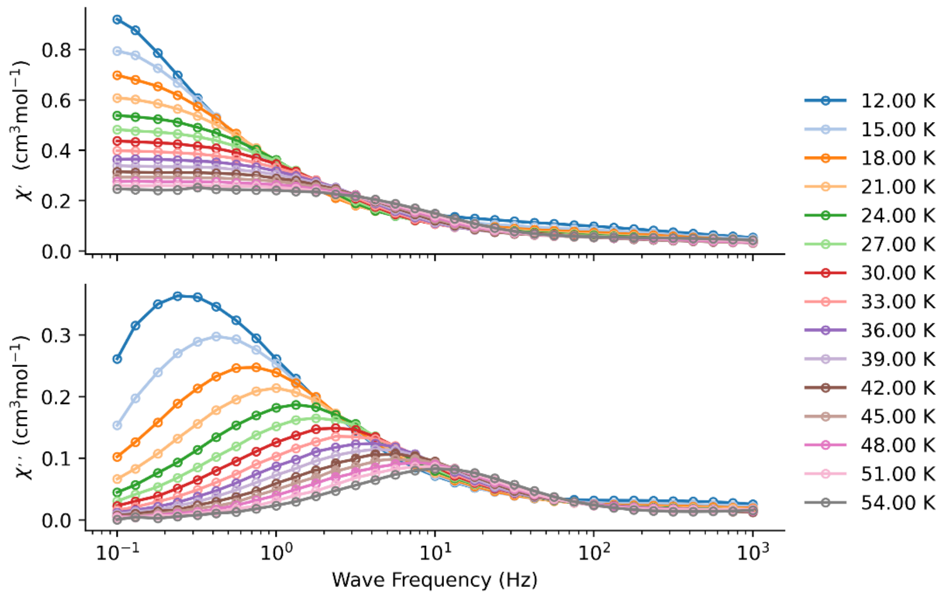

Figure S90. Unedited in-phase (top) and out-of-phase (bottom) ac susceptibilities of **2-Dy** in a zero field from 12–54 K.

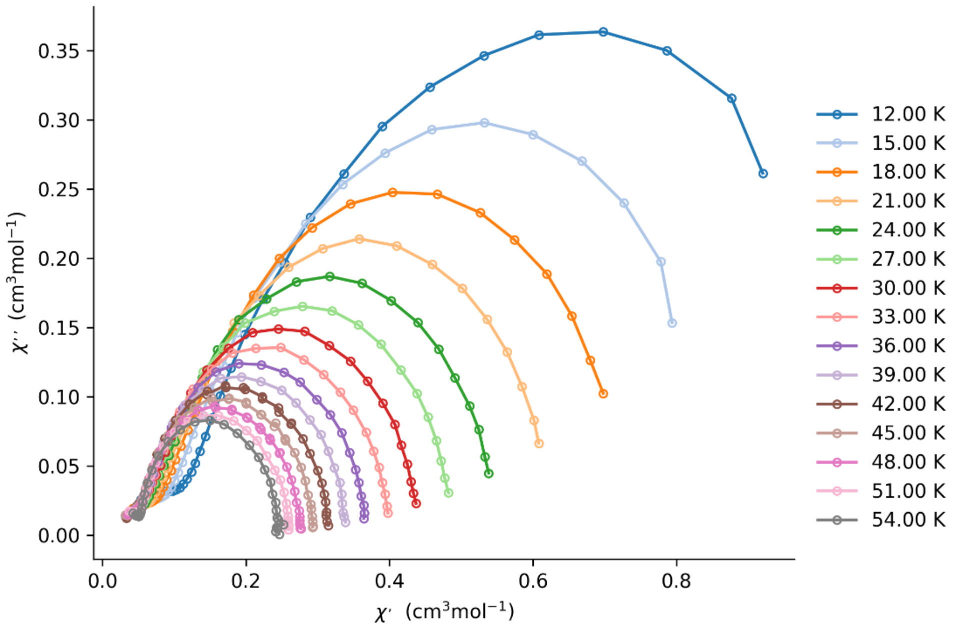

Figure S91. Unedited Cole-Cole plot showing ac data for **2-Dy** in a zero dc field from 12–54 K.

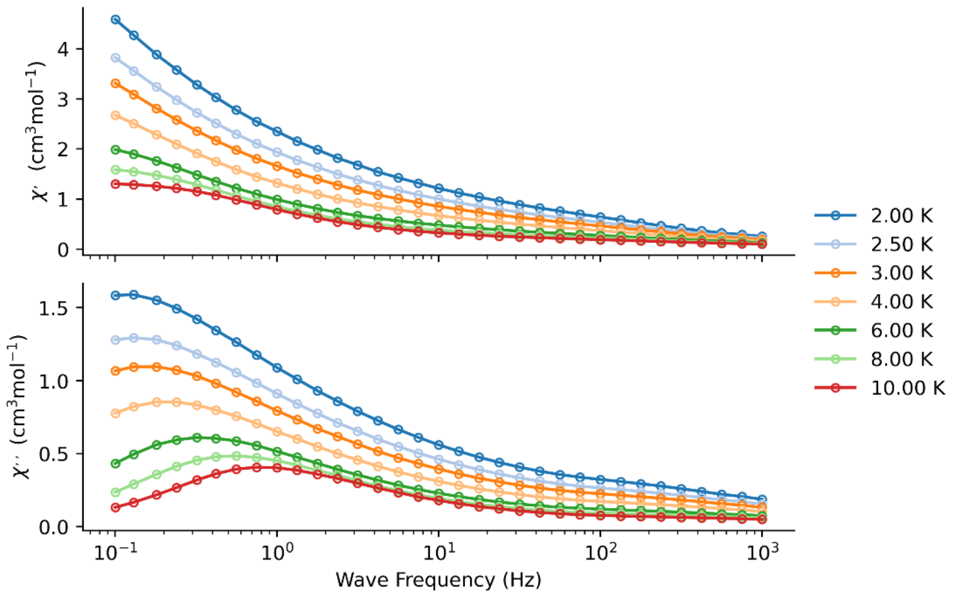

Figure S92. Unedited in-phase (top) and out-of-phase (bottom) ac susceptibilities of **3-Dy** in a zero field from 2–10 K.

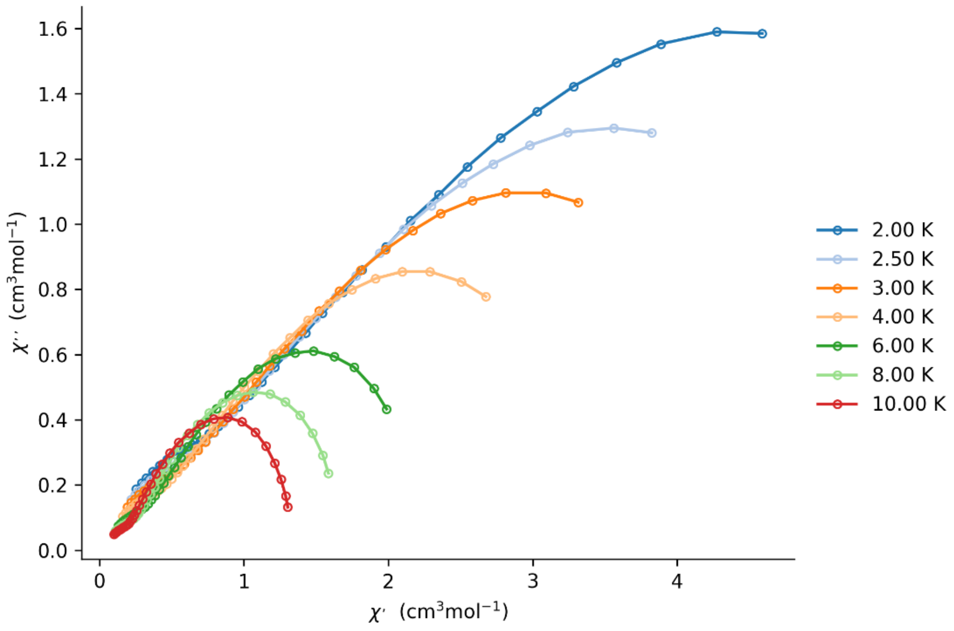

Figure S93. Unedited Cole-Cole plot showing ac data for **3-Dy** in a zero dc field from 2–10 K.

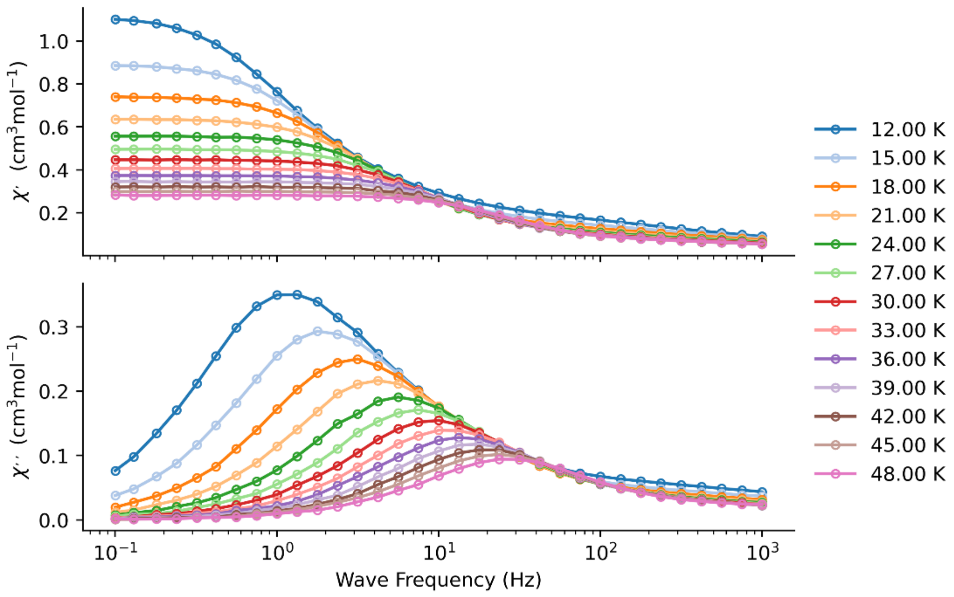

Figure S94. Unedited in-phase (top) and out-of-phase (bottom) ac susceptibilities of **3-Dy** in a zero field from 12–48 K.

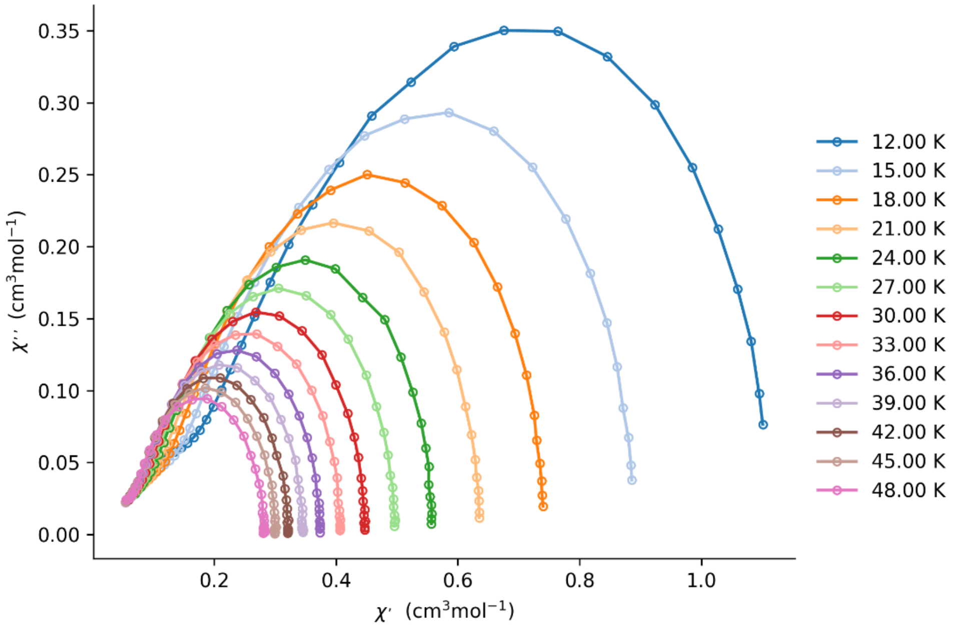

Figure S95. Unedited Cole-Cole plot showing ac data for **3-Dy** in a zero dc field from 12–48 K.

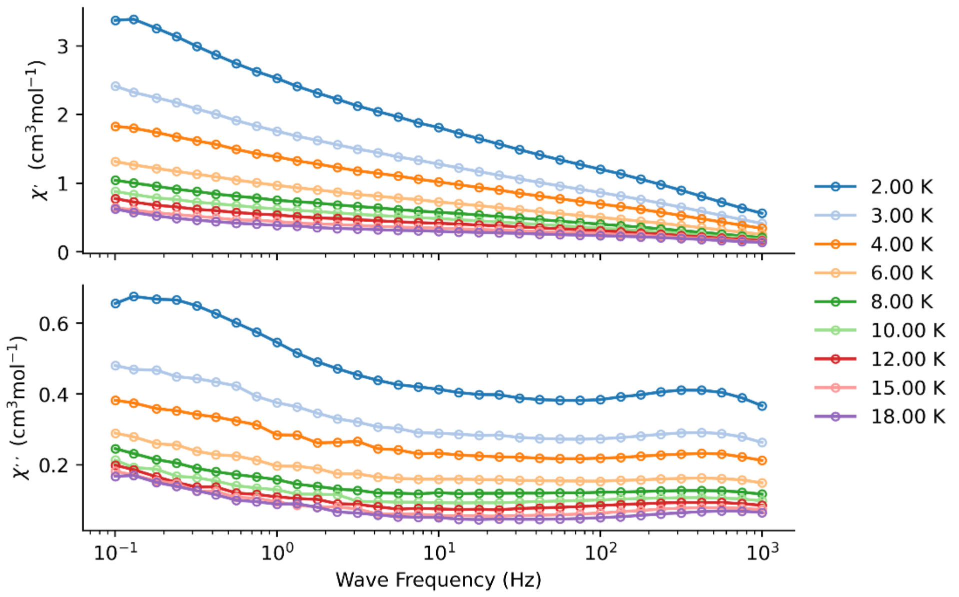

Figure S96. Unedited in-phase (top) and out-of-phase (bottom) ac susceptibilities of **4-Dy** in a zero field from 2–18 K.

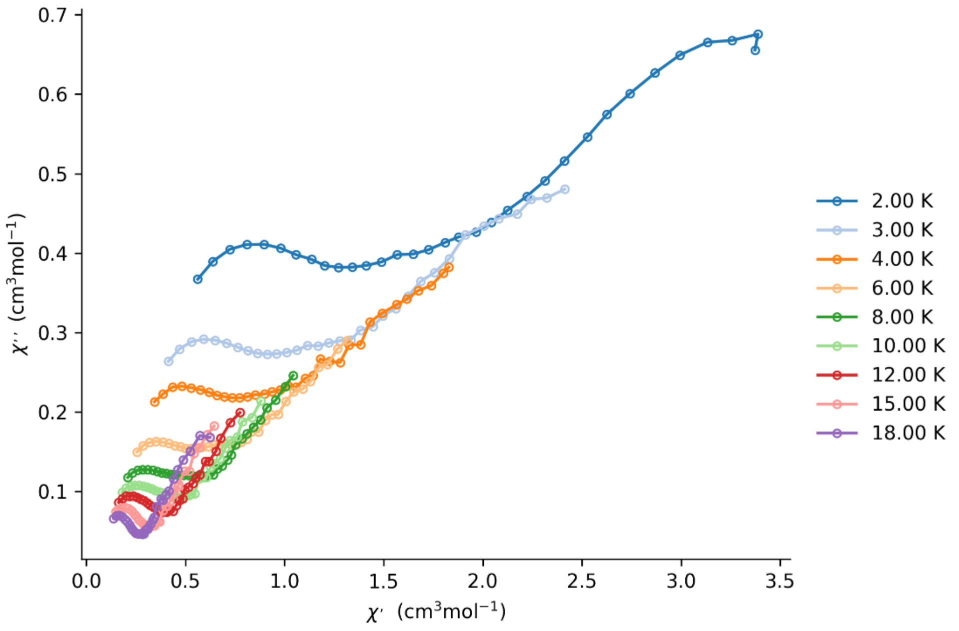

Figure S97. Unedited Cole-Cole plot showing ac data for **4-Dy** in a zero dc field from 2–18 K.

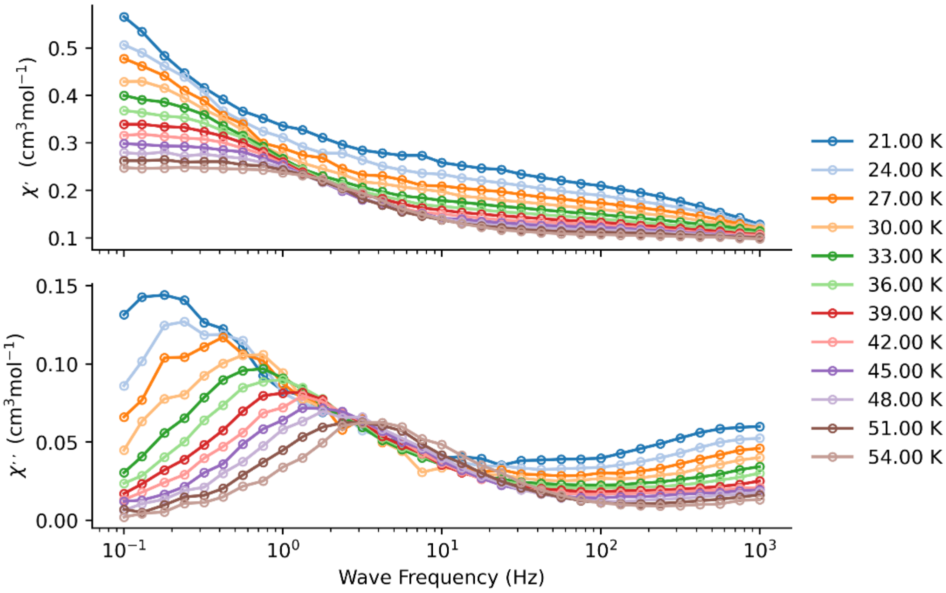

Figure S98. Unedited in-phase (top) and out-of-phase (bottom) ac susceptibilities of **4-Dy** in a zero field from 21–54 K.

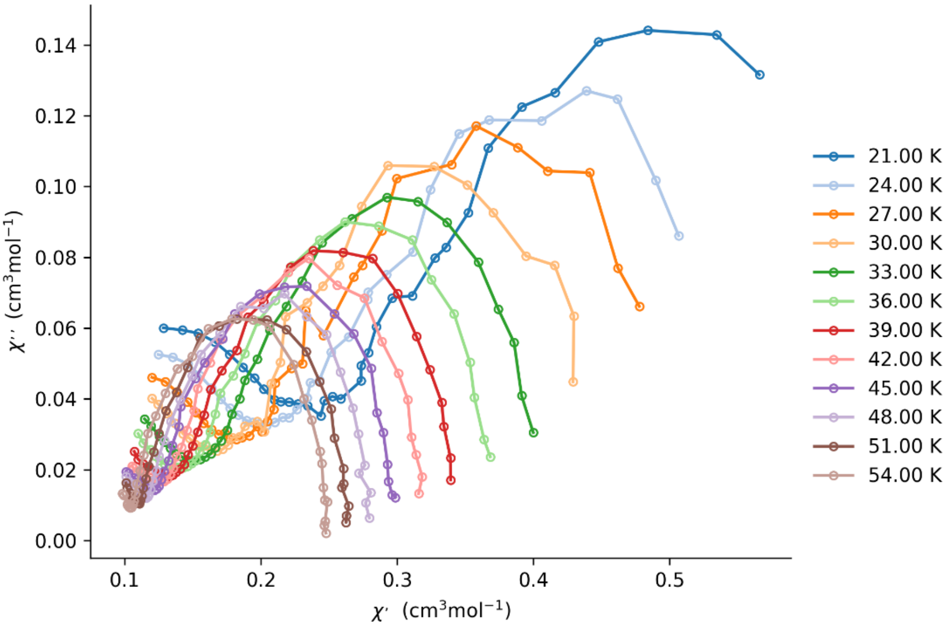

Figure S99. Unedited Cole-Cole plot showing fitting of ac data for **4-Dy** in a zero dc field from 21–54 K.

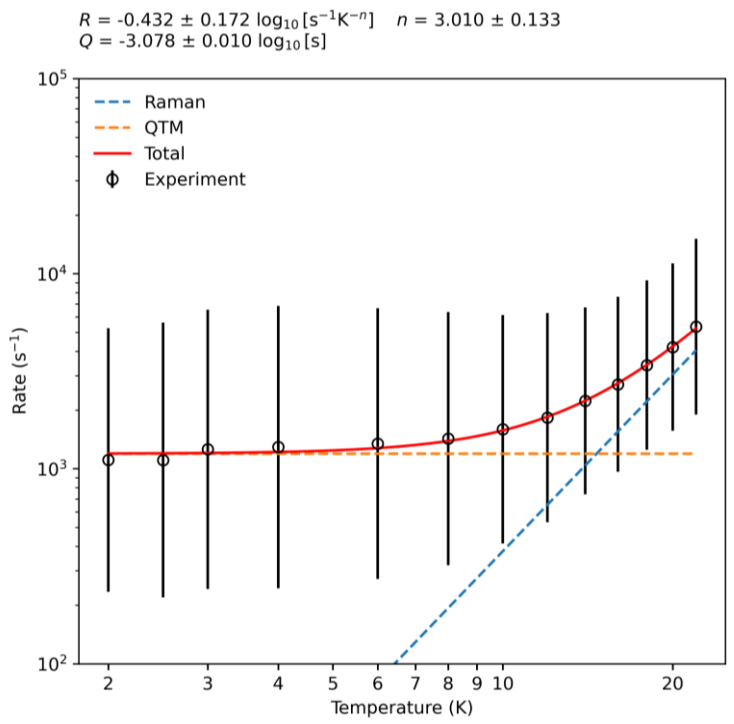

Figure S100. Fitting "[{Dy(Cp*)$_2$}{Al[OC(CF$_3$)$_3$]$_4$}]" relaxation profile using CC-FIT2,[22,23] showing Raman and QTM components. Error bars represent one ESD in the distribution of rates.

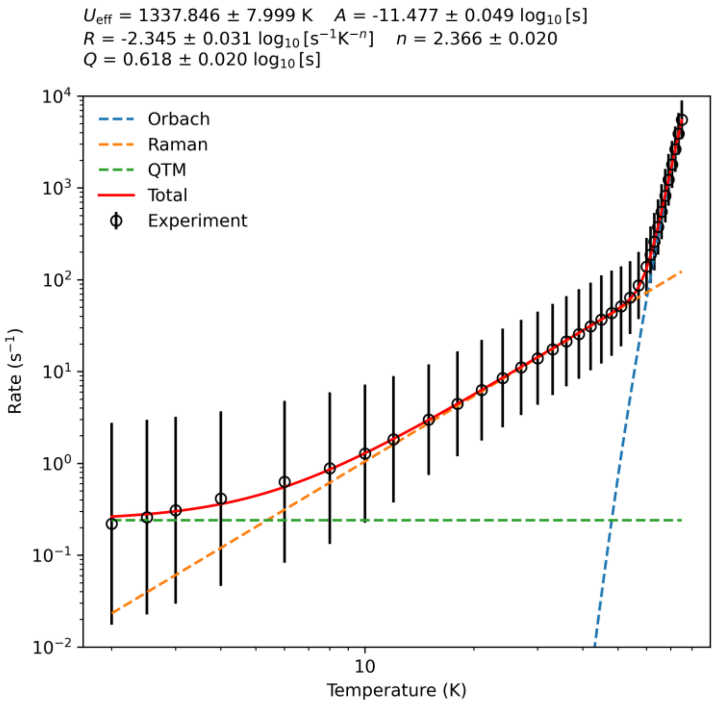

Figure S101. Fitting **2-Dy** relaxation profile using CC-FIT2,[22,23] showing Orbach and Raman components. Error bars represent one ESD in the distribution of rates.

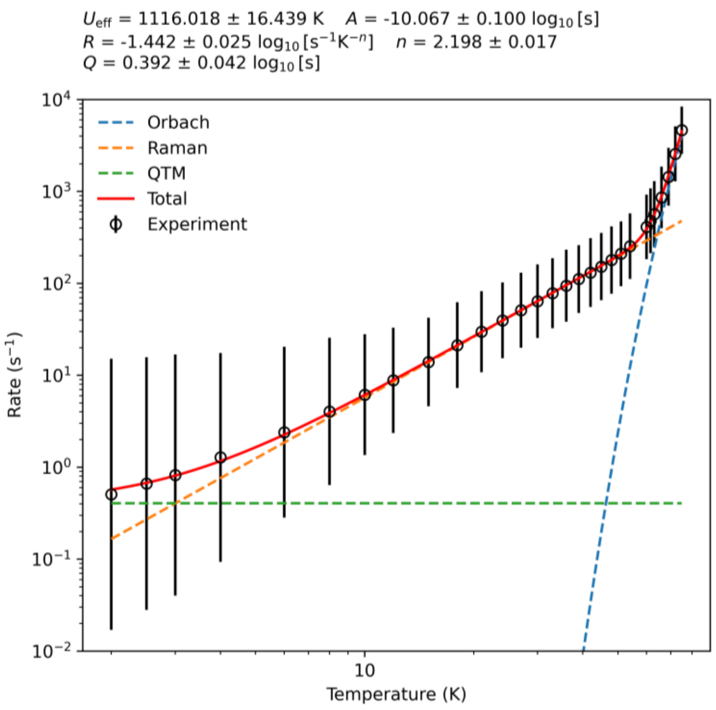

Figure S102. Fitting **3-Dy** relaxation profile using CC-FIT2,[22,23] showing Orbach and Raman components. Error bars represent one ESD in the distribution of rates.

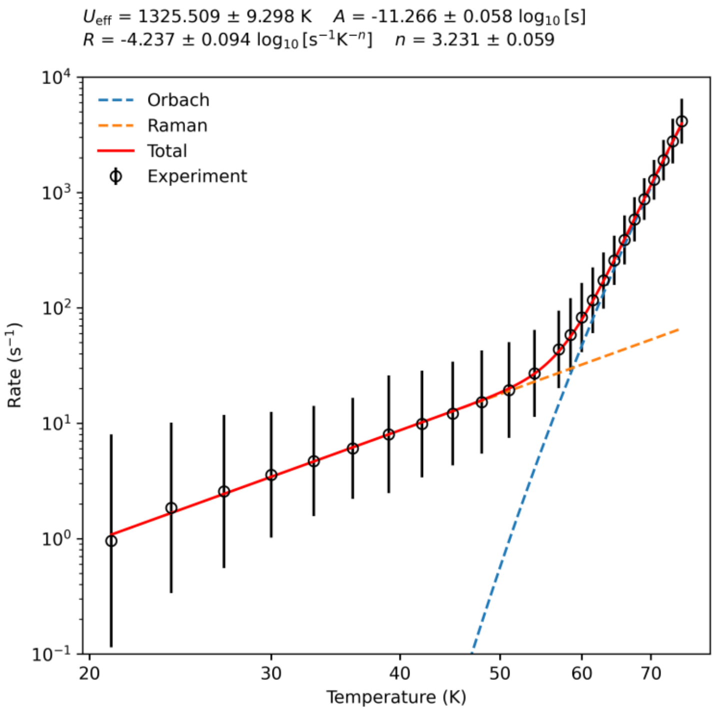

Figure S103. Fitting **4-Dy** relaxation profile using CC-FIT2,[22,23] showing Orbach and Raman components. Error bars represent one ESD in the distribution of rates.

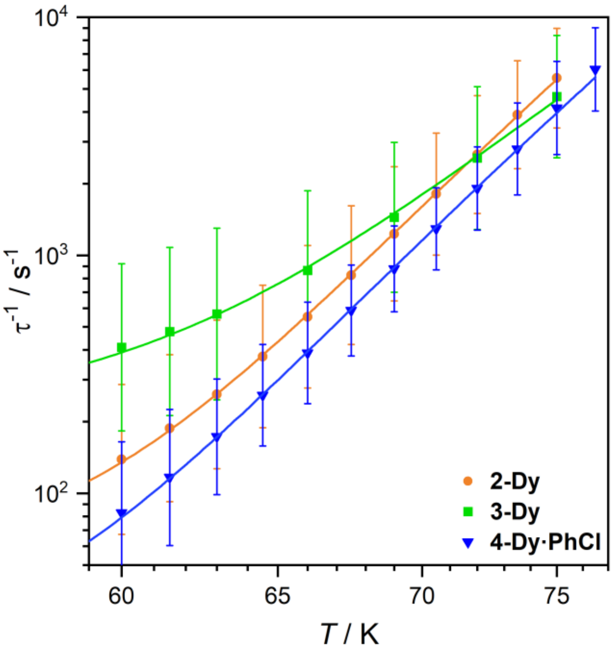

Figure S104. Comparison of high-temperature relaxation profiles of **2-4-Dy**. Error bars represent one ESD in the distribution of rates.

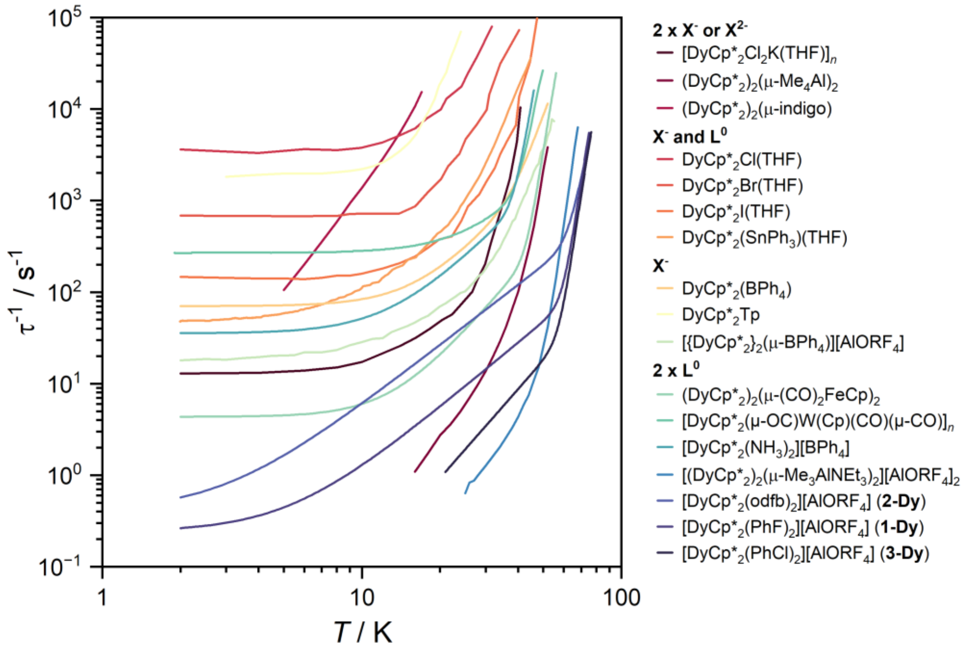

Figure S105. Comparison of rates for {DyCp*$_2$}-containing SMMs with neutral and anionic (non-radical) ligands.[24–32] A combination of rates as reported τ values, rates simulated from fitted relaxation profile parameters (if reported), and rates extracted from figures have been used.

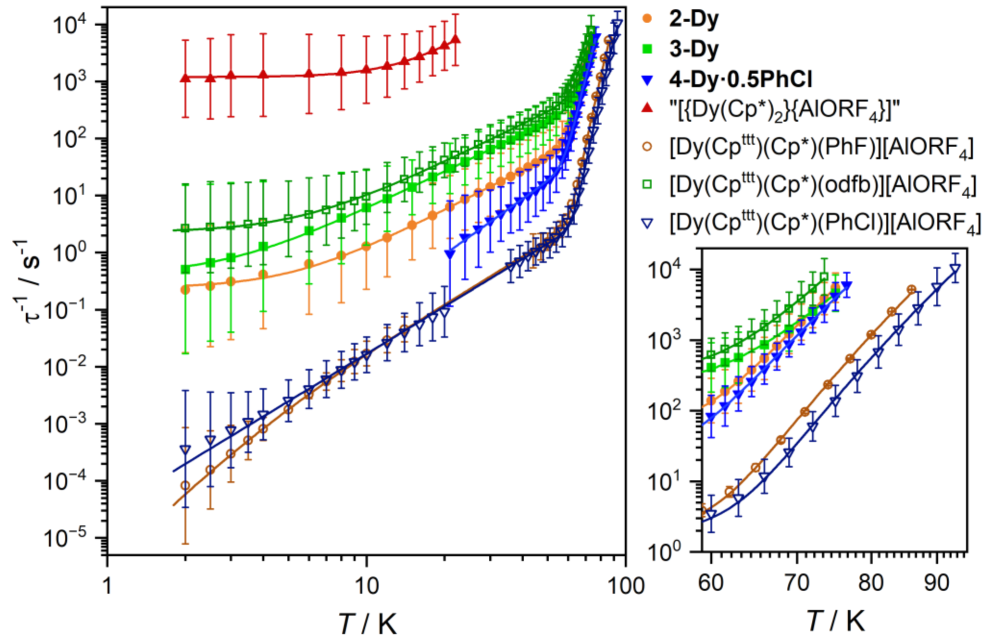

Figure S106. Relaxation Profiles of **2-Dy**, **3-Dy**, **4-Dy·0.5PhCl**, "[{Dy(Cp*)$_2$}{Al[OC(CF$_3$)$_3$]$_4$}]" and halobenzene-adducts of {Dy(Cp^{ttt})(Cp*)}.[33] Error bars represent one ESD in the distribution of rates.

9. CASSCF-SO Calculations

OpenMolcas[34] was used to perform CASSCF-SO calculations on $[Dy(Cp^*)_2(PhF\text{-}\kappa\text{-}F)_2]^+$ (**2′-Dy**), $[Dy(Cp^*)_2(C_6H_4F_2\text{-}\kappa^2\text{-}F,F)(C_6H_4F_2\text{-}\kappa\text{-}F)]^+$ (**3′-Dy**), $[Dy(Cp^*)_2(PhCl\text{-}\kappa\text{-}Cl)_2]^+$ (**4′-Dy**), and $[Dy(Cp^*)_2(PhBr\text{-}\kappa\text{-}Br)_2]^+$ (**5′-Dy)** to determine their electronic structures. For **2′-5′-Dy**, the molecular geometries from the single crystal XRD structures were used with no optimization, taking the largest disorder component only. For $[Dy(Cp^*)_2][Al\{OC(CF_3)_3\}_4]$ and $[Dy(Cp^*)_2(PhBr\text{-}\kappa\text{-}I)_2]^+$ (**15′-Dy**) the molecular geometries were obtained from DFT optimisation of their Y analogues, initially constructed from the atom coordinates of **4-Dy**. Integrals were performed in the SEWARD module using basis sets from ANO-RCC library[35–38] with VTZP quality for Dy atoms, VDZP quality for the cyclopentadienyl C atoms, the fluorobenzene F atoms (**2′-Dy**), the *ortho*-difluorobenzene F atoms (**3′-Dy**), the chlorobenzene Cl atoms (**4′-Dy**), bromobenzene Br atoms (**5′-Dy**) and iodobenzene I atoms (**15′-Dy**) and VDZ quality for all remaining atoms, employing the second-order DKH transformation. Cholesky decomposition of the two-electron integrals with a threshold of 10^{-8} was performed to save disk space and reduce computational demand. The molecular orbitals (MOs) were optimized in state-averaged CASSCF calculations in the RASSCF module, where the active space was defined by the nine 4f electrons in the seven 4f orbitals of Dy(III). Three such calculations were performed independently for each possible spin state, where 21 roots were included for $S = 5/2$, 224 roots were included for $S = 3/2$, and 490 roots were included for $S = 1/2$. The wavefunctions obtained from these CASSCF calculations were then mixed by spin orbit coupling in the RASSI module, where all 21 $S = 5/2$ states, 128 of the $S = 3/2$ states, and 130 of the $S = 1/2$ states were included**.** SINGLE_ANISO was used to decompose the resulting spin-orbit wave functions into the CF Hamiltonian formalism.[39] Diamond was employed for molecular graphics.[13]

Table S19. Electronic structure of [Dy(Cp*)$_2$][Al{OC(CF$_3$)$_3$}$_4$] calculated with the crystal field parameters obtained from CASSCF-SO using the atom coordinates obtained through DFT optimization. Each row corresponds to a Kramers doublet.

Energy (cm^{-1})	Energy (K)	g_x	g_y	g_z	Angle[a] (deg)	Wavefunction	$\langle J_z \rangle$
0.00	0.00	3E–6	3E–6	19.9	--	99.4% |± 15/2⟩	±7.489
454.94	654.64	0.0001	0.0001	17.0	1.2	99.8% |± 13/2⟩	±6.496
759.37	1092.71	0.001	0.002	14.4	1.4	99.0% |± 11/2⟩	±5.505
970.75	1396.88	0.03	0.03	11.7	2.1	99.2% |± 9/2⟩	±4.493
1146.88	1650.33	0.2	0.3	9.1	4.1	99.0% |± 7/2⟩	±3.489
1283.97	1847.61	2.3	3.8	6.1	12.4	91% |± 5/2⟩ + 7% |± 1/2⟩	±2.314
1368.64	1969.44	2.2	6.5	10.7	89.7	72% |± 3/2⟩ + 21% |∓ 1/2⟩ + 5% |∓ 5/2⟩	±0.870
1507.85	2169.77	0.1	0.2	18.3	89.4	57% |± 1/2⟩ + 19% |∓ 3/2⟩ + 14% |∓ 1/2⟩ + 7% |± 3/2⟩	±0.083

[a] The angle between the g_z value of the excited Kramers doublet and the ground Kramers doublet.

Table S20. Electronic structure of **2′-Dy** calculated with the crystal field parameters obtained from CASSCF-SO using the solid state geometry of **2-Dy** in zero-field. Each row corresponds to a Kramers doublet.

Energy (cm^{-1})	Energy (K)	g_x	g_y	g_z	Angle[a] (deg)	Wavefunction	$<J_z>$
0.00	0.00	1E–5	4E–5	19.8	--	98% |± 15/2⟩	±7.463
323.95	466.16	0.002	0.002	17.0	0.9	98.7% |± 13/2⟩	±6.474
540.94	778.41	0.03	0.03	14.5	1.5	98% |± 11/2⟩	±5.527
673.88	969.71	0.3	0.4	11.8	1.6	98% |± 9/2⟩	±4.500
766.32	1102.73	1.7	3.3	8.4	6.9	87% |± 7/2⟩ + 10% |± 3/2⟩	±3.177
810.11	1165.75	2.7	7.8	8.9	89.6	50% |± 5/2⟩ + 34% |± 1/2⟩ + 9% |∓ 3/2⟩ 6% |∓ 7/2⟩	±1.089
881.32	1268.21	0.4	1.2	15.3	89.3	44% |± 3/2⟩ + 35% |∓ 5/2⟩ + 10% |∓ 1/2⟩	±0.141
1089.51	1567.80	0.02	0.04	19.5	89.6	31% |± 1/2⟩ + 22% |∓ 1/2⟩ + 19% |∓ 3/2⟩ + 13% |± 3/2⟩ + 6% |± 5/2⟩	±0.054

[a] The angle between the g_z value of the excited Kramers doublet and the ground Kramers doublet.

Table S21. Electronic structure of **3′-Dy** calculated with the crystal field parameters obtained from CASSCF-SO using the solid state geometry of **3-Dy** in zero-field. Each row corresponds to a Kramers doublet.

Energy (cm^{-1})	Energy (K)	g_x	g_y	g_z	Angle[a] (deg)	Wavefunction	$\langle J_z \rangle$
0.00	0.00	0.0002	0.0003	19.8	--	98.9% $\|\pm 15/2\rangle$	±7.478
349.67	503.17	0.004	0.004	17.1	0.7	99.6% $\|\pm 13/2\rangle$	±6.491
575.56	828.23	0.03	0.03	14.5	0.8	98.7% $\|\pm 11/2\rangle$	± 5.517
720.97	1037.47	0.1	0.2	11.8	0.6	99.1% $\|\pm 9/2\rangle$	±4.493
836.75	1204.09	2.5	3.2	8.7	0.8	96% $\|\pm 7/2\rangle$	±3.345
915.59	1317.53	4.0	4.2	11.0	89.1	69% $\|\pm 5/2\rangle$ + 19% $\|\pm 1/2\rangle$ + 10% $\|\mp 3/2\rangle$	±1.613
982.21	1413.40	0.5	1.2	13.7	89.9	58% $\|\pm 3/2\rangle$ + 24% $\|\mp 5/2\rangle$ + 16% $\|\mp 1/2\rangle$	±0.240
1113.10	1601.75	0.06	0.2	18.7	89.9	61% $\|\pm 1/2\rangle$ + 28% $\|\mp 3/2\rangle$	±0.021

[a] The angle between the g_z value of the excited Kramers doublet and the ground Kramers doublet.

Table S22. Electronic structure of **4′-Dy** calculated with the crystal field parameters obtained from CASSCF-SO using the solid state geometry of **4-Dy** in zero-field. Each row corresponds to a Kramers doublet.

Energy (cm^{-1})	Energy (K)	g_x	g_y	g_z	Angle[a] (deg)	Wavefunction	$<J_z>$
0.00	0.00	2E–5	3E–5	19.8	--	98.5% |± 15/2⟩	±7.470
317.57	456.99	0.0002	0.0002	17.1	0.3	99.2% |± 13/2⟩	±6.484
549.26	790.38	0.006	0.006	14.5	0.6	98% |± 11/2⟩	±5.526
707.83	1018.57	0.2	0.2	11.8	0.9	98.7% |± 9/2⟩	±4.504
825.27	1187.56	2.0	2.6	8.6	5.0	91% |± 7/2⟩ + 7% |± 3/2⟩	±3.272
888.74	1278.90	3.3	7.0	9.2	89.0	56% |± 5/2⟩ + 31% |± 1/2⟩ + 9% |∓ 5/2⟩	±1.295
964.34	1387.68	0.2	0.7	14.7	89.7	27% |± 3/2⟩ + 25% |∓ 3/2⟩ + 19% |∓ 5/2⟩ + 15% |∓ 5/2⟩ + 6% |± 1/2⟩ + 6% |∓ 1/2⟩	±0.057
1161.07	1670.78	0.03	0.07	19.3	90.0	56% |± 1/2⟩ + 32% |∓ 3/2⟩ + 9% |± 5/2⟩	±0.013

[a] The angle between the g_z value of the excited Kramers doublet and the ground Kramers doublet.

Table S23. Electronic structure of **5′-Dy** calculated with the crystal field parameters obtained from CASSCF-SO using the solid state geometry of **5-Dy** in zero-field. Each row corresponds to a Kramers doublet.

Energy (cm^{-1})	Energy (K)	g_x	g_y	g_z	Angle[a] (deg)	Wavefunction	$<J_z>$
0.00	0.00	2E–5	3E–5	19.8	--	98% |± 15/2⟩	±7.468
315.21	453.59	0.0003	0.0003	17.0	0.4	99.0% |± 13/2⟩	±6.480
546.71	786.72	0.003	0.003	14.5	0.6	98% |± 11/2⟩	±5.526
705.24	1014.84	0.2	0.2	11.8	1.0	98% |± 9/2⟩	±4.501
821.24	1181.76	2.2	2.9	8.5	6.6	89% |± 7/2⟩ + 8% |± 3/2⟩	±3.222
884.36	1272.59	3.1	7.1	9.1	89.1	55% |± 5/2⟩ + 31% |± 1/2⟩ + 9% |∓ 3/2⟩ + 5% |∓ 7/2⟩	±1.239
966.42	1390.67	0.2	0.6	14.9	89.7	30% |± 3/2⟩+ 22% |∓ 5/2⟩+ 21% |∓ 3/2⟩ + 13% |± 5/2⟩+ 6% |∓ 1/2⟩ + 5% |± 1/2⟩+	±0.064
1164.66	1675.94	0.03	0.06	19.3	90.0	54% |± 1/2⟩ + 31% |∓ 3/2⟩ + 9% |± 5/2⟩	±0.013

[a] The angle between the g_z value of the excited Kramers doublet and the ground Kramers doublet.

Table S24. Electronic structure of **15′-Dy** calculated with the crystal field parameters obtained from CASSCF-SO using the atom coordinates obtained through DFT optimisation. Each row corresponds to a Kramers doublet.

Energy (cm^{-1})	Energy (K)	g_x	g_y	g_z	Angle[a] (deg)	Wavefunction	$\langle J_z \rangle$
0.00	0.00	4E–5	5E–5	19.8	--	98% |± 15/2⟩	±7.468
310.19	446.36	0.0009	0.0009	17.1	0.5	99.0% |± 13/2⟩	±6.481
525.31	755.92	0.008	0.009	14.5	1.0	98% |± 11/2⟩	±5.528
668.92	962.57	0.1	0.2	11.8	0.7	98% |± 9/2⟩	±4.507
778.09	1119.67	2.0	2.7	8.6	3.5	91% |± 7/2⟩ + 7% |± 3/2⟩	±3.268
842.77	1212.75	3.4	7.6	8.2	88.6	59% |± 5/2⟩ + 27% |± 1/2⟩ + 9% |∓ 3/2⟩ + 5% |∓ 7/2⟩	±1.337
920.50	1324.61	0.08	0.3	14.6	89.2	40% |± 3/2⟩ + 23% |∓ 5/2⟩ + 13% |∓ 3/2⟩ + 10% |∓ 1/2⟩ + 9% |± 5/2⟩	±0.098
1098.95	1581.39	0.04	0.08	19.2	89.8	43% |± 1/2⟩ + 24% |∓ 3/2⟩ + 16% |∓ 1/2⟩ + 8% |± 3/2⟩ + 6% |± 5/2⟩	±0.026

[a] The angle between the g_z value of the excited Kramers doublet and the ground Kramers doublet.

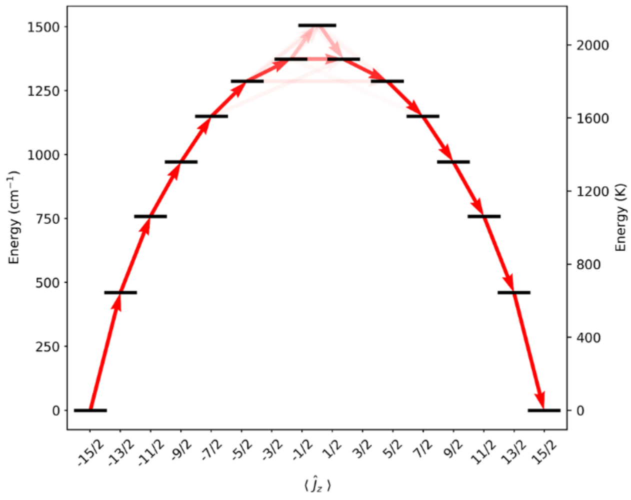

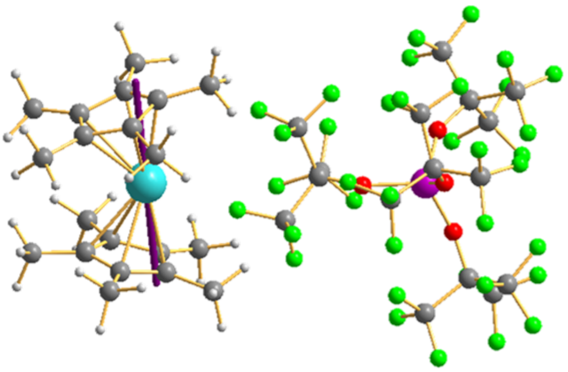

Figure S107. Energy barrier to magnetic relaxation for a model of [Dy(Cp*)$_2$][Al{OC(CF$_3$)$_3$}$_4$]. Electronic states from CASSCF-SO calculations, labelled with their dominant m_J composition in the J = 15/2 basis. Arrows represent the Orbach relaxation pathway, where the opacity of the arrows is proportional to the transition probability approximated with the average matrix elements of magnetic moment connecting the states, $\gamma_{ij} = (1/3)[|\langle i|\mu_x|j\rangle|^2 + |\langle i|\mu_y|j\rangle|^2 + |\langle i|\mu_z|j\rangle|^2]$, normalized from each departing state and commencing from |–15/2> (left). Denotation of the g_z axis (purple) within the solid state structure at the ground state (right).

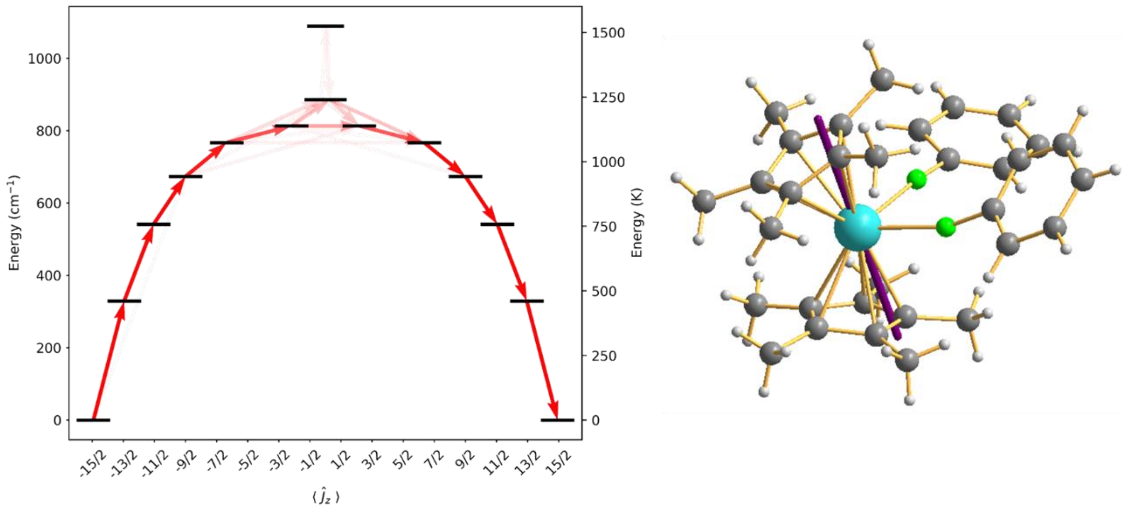

Figure S108. Energy barrier to magnetic relaxation for a model of **2′-Dy**. Electronic states from CASSCF-SO calculations, labelled with their dominant m_J composition in the J = 15/2 basis. Arrows represent the Orbach relaxation pathway, where the opacity of the arrows is proportional to the transition probability approximated with the average matrix elements of magnetic moment connecting the states, $\gamma_{ij} = (1/3)[|\langle i|\mu_x|j\rangle|^2 + |\langle i|\mu_y|j\rangle|^2 + |\langle i|\mu_z|j\rangle|^2]$, normalized from each departing state and commencing from |–15/2> (left). Denotation of the g_z axis (purple) within the solid state structure at the ground state (right).

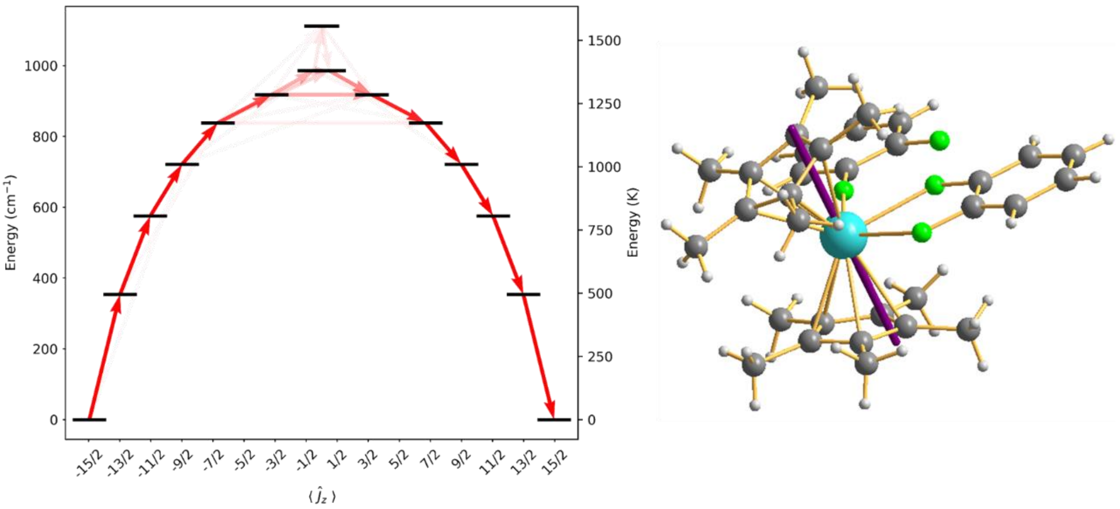

Figure S109. Energy barrier to magnetic relaxation for a model of **3′-Dy**. Electronic states from CASSCF-SO calculations, labelled with their dominant m_J composition in the J = 15/2 basis. Arrows represent the Orbach relaxation pathway, where the opacity of the arrows is proportional to the transition probability approximated with the average matrix elements of magnetic moment connecting the states, $\gamma_{ij} = (1/3)[|<i|\mu_x|j>|^2 + |<i|\mu_y|j>|^2 + |<i|\mu_z|j>|^2]$, normalized from each departing state and commencing from $|-15/2>$ (left). Denotation of the g_z axis (purple) within the solid state structure at the ground state (right).

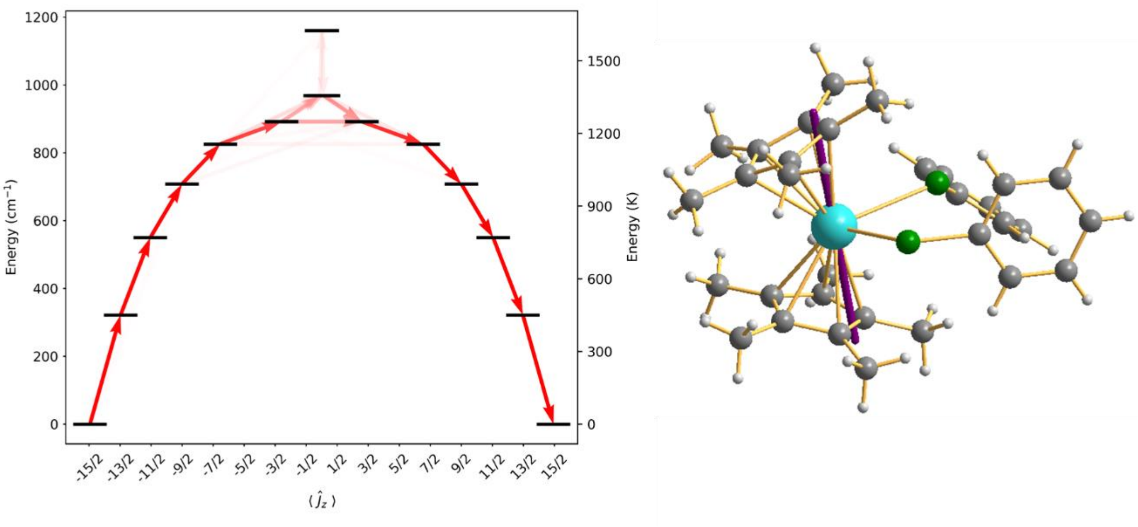

Figure S110. Energy barrier to magnetic relaxation for a model of **4′-Dy**. Electronic states from CASSCF-SO calculations, labelled with their dominant m_J composition in the J = 15/2 basis. Arrows represent the Orbach relaxation pathway, where the opacity of the arrows is proportional to the transition probability approximated with the average matrix elements of magnetic moment connecting the states, $\gamma_{ij} = (1/3)[|\langle i|\mu_x|j\rangle|^2 + |\langle i|\mu_y|j\rangle|^2 + |\langle i|\mu_z|j\rangle|^2]$, normalized from each departing state and commencing from |–15/2> (left). Denotation of the g_z axis (purple) within the solid state structure at the ground state (right).

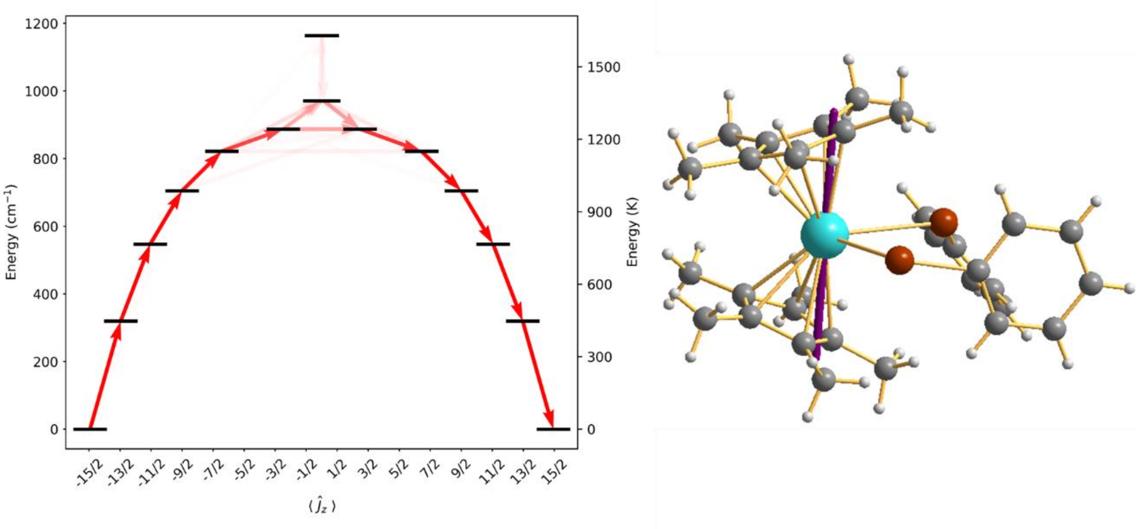

Figure S111. Energy barrier to magnetic relaxation for a model of **5′-Dy**. Electronic states from CASSCF-SO calculations, labelled with their dominant m_J composition in the J = 15/2 basis. Arrows represent the Orbach relaxation pathway, where the opacity of the arrows is proportional to the transition probability approximated with the average matrix elements of magnetic moment connecting the states, $\gamma_{ij} = (1/3)[|\langle i|\mu_x|j\rangle|^2 + |\langle i|\mu_y|j\rangle|^2 + |\langle i|\mu_z|j\rangle|^2]$, normalized from each departing state and commencing from |–15/2> (left). Denotation of the g_z axis (purple) within the solid state structure at the ground state (right).

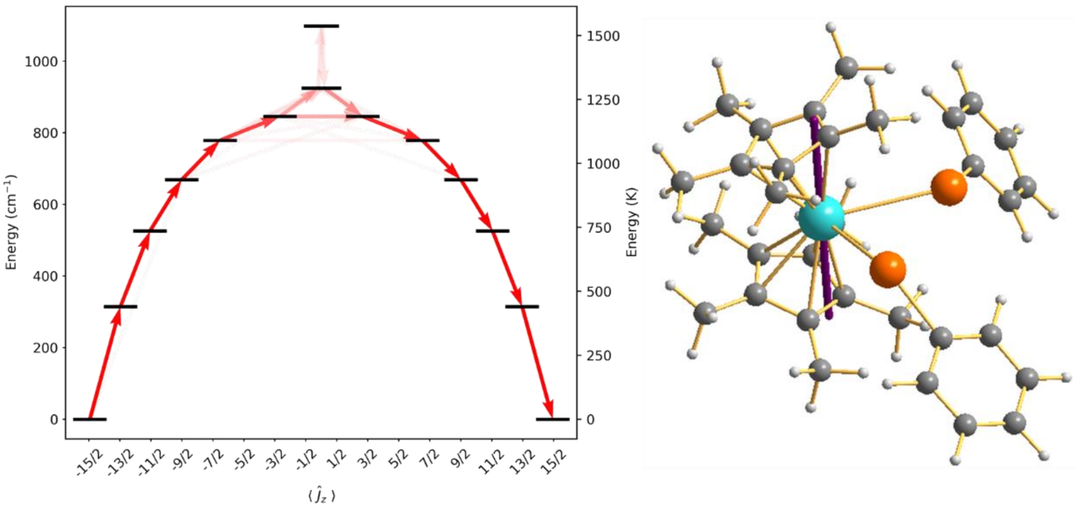

Figure S112. Energy barrier to magnetic relaxation for a model of **15′-Dy**. Electronic states from CASSCF-SO calculations, labelled with their dominant m_J composition in the J = 15/2 basis. Arrows represent the Orbach relaxation pathway, where the opacity of the arrows is proportional to the transition probability approximated with the average matrix elements of magnetic moment connecting the states, $\gamma_{ij} = (1/3)[|<i|\mu_x|j>|^2 + |<i|\mu_y|j>|^2 + |<i|\mu_z|j>|^2]$, normalized from each departing state and commencing from |–15/2> (left). Denotation of the g_z axis (purple) within the solid state structure at the ground state (right).

10. References

(1) Ortu, F.; Packer, D.; Liu, J.; Burton, M.; Formanuik, A.; Mills, D. P. Synthesis and Structural Characterization of Lanthanum and Cerium Substituted Cyclopentadienyl Borohydride Complexes. *J. Organomet. Chem.* **2018**, *857,* 45–51. https://doi.org/10.1016/j.jorganchem.2017.09.010.

(2) Rabe, G.; Roesky, H. W.; Stalke, D.; Pauer, F.; Sheldrick, G. M. The Preparation and Crystal Structures of Sodium and Potassium Pentamethylcyclopentadienyl Pyridine Solvates. *J. Organomet. Chem.* **1991**, *403* (1–2), 11–19. https://doi.org/10.1016/0022-328X(91)83082-F.

(3) Krossing, I.; Brands, H.; Feuerhake, R.; Koenig, S. New Reagents to Introduce Weakly Coordinating Anions of Type $Al(ORF)_4^-$: Synthesis, Structure and Characterization of Cs and Trityl Salts. *J. Fluor. Chem.* **2001**, *112* (1), 83–90. https://doi.org/10.1016/S0022-1139(01)00490-0.

(4) Chien, J. C. W.; Tsai, W. M.; Rausch, M. D. Isospecific Polymerization of Propylene Catalyzed by Rac-Ethylenebis(Indenyl)Methylzirconium Cation. *J. Am. Chem. Soc.* **1991**, *113* (22), 8570–8571. https://doi.org/10.1021/ja00022a081.

(5) Kabova, E. A.; Blundell, C. D.; Muryn, C. A.; Whitehead, G. F. S.; Vitorica-Yrezabal, I. J.; Ross, M. J.; Shankland, K. SDPD-SX: Combining a Single Crystal X-Ray Diffraction Setup with Advanced Powder Data Structure Determination for Use in Early Stage Drug Discovery. *CrystEngComm* **2022**, *24* (24), 4337–4340. https://doi.org/10.1039/D2CE00387B.

(6) *CrysAlis Pro*; Agilent Technologies: Yarnton, England, 2010.

(7) Pawley, G. S. Unit-Cell Refinement from Powder Diffraction Scans. *J. Appl. Crystallogr.* **1981**, *14* (6), 357–361. https://doi.org/10.1107/S0021889881009618.

(8) Coelho, A. A. An Indexing Algorithm Independent of Peak Position Extraction for X-

Ray Powder Diffraction Patterns. *J. Appl. Crystallogr.* **2017**, *50* (5), 1323–1330. https://doi.org/10.1107/S1600576717011359.

(9) Sheldrick, G. M. Crystal Structure Refinement with SHELXL. *Acta Crystallogr. Sect. C Struct. Chem.* **2015**, *71* (1), 3–8. https://doi.org/10.1107/S2053229614024218.

(10) Dolomanov, O. V.; Bourhis, L. J.; Gildea, R. J.; Howard, J. A. K.; Puschmann, H. OLEX2 : A Complete Structure Solution, Refinement and Analysis Program. *J. Appl. Crystallogr.* **2009**, *42* (2), 339–341. https://doi.org/10.1107/S0021889808042726.

(11) Farrugia, L. J. WinGX and ORTEP for Windows : An Update. *J. Appl. Crystallogr.* **2012**, *45* (4), 849–854. https://doi.org/10.1107/S0021889812029111.

(12) *POV-Ray*; Persistence of Vision Raytracer Pty. Ltd.: Williamstown, Australia, 2004.

(13) *Diamond - Crystal and Molecular Structure Visualization*; Crystal Impact - Dr. H. Putz & Dr. K. Brandenburg GbR: Bonn, Germany.

(14) Neese, F. The ORCA Program System. *WIREs Comput. Mol. Sci.* **2012**, *2* (1), 73–78. https://doi.org/10.1002/wcms.81.

(15) Neese, F. Software Update: The ORCA Program System, Version 4.0. *WIREs Comput. Mol. Sci.* **2018**, *8* (1), e1327. https://doi.org/10.1002/wcms.1327.

(16) Lehtola, S.; Steigemann, C.; Oliveira, M. J. T.; Marques, M. A. L. Recent Developments in LIBXC — A Comprehensive Library of Functionals for Density Functional Theory. *SoftwareX* **2018**, *7*, 1–5. https://doi.org/10.1016/j.softx.2017.11.002.

(17) Caldeweyher, E.; Bannwarth, C.; Grimme, S. Extension of the D3 Dispersion Coefficient Model. *J. Chem. Phys.* **2017**, *147* (3), 034112. https://doi.org/10.1063/1.4993215.

(18) Caldeweyher, E.; Ehlert, S.; Hansen, A.; Neugebauer, H.; Spicher, S.; Bannwarth, C.; Grimme, S. A Generally Applicable Atomic-Charge Dependent London Dispersion Correction. *J. Chem. Phys.* **2019**, *150* (15), 154122. https://doi.org/10.1063/1.5090222.

(19) Weigend, F.; Ahlrichs, R. Balanced Basis Sets of Split Valence, Triple Zeta Valence and Quadruple Zeta Valence Quality for H to Rn: Design and Assessment of Accuracy. *Phys. Chem. Chem. Phys.* **2005**, *7* (18), 3297–3305. https://doi.org/10.1039/b508541a.

(20) Weigend, F. Accurate Coulomb-Fitting Basis Sets for H to Rn. *Phys. Chem. Chem. Phys.* **2006**, *8* (9), 1057. https://doi.org/10.1039/b515623h.

(21) Andrae, D.; Häußermann, U.; Dolg, M.; Stoll, H.; Preuß, H. Energy-Adjusted Ab Initio Pseudopotentials for the Second and Third Row Transition Elements. *Theor. Chim. Acta* **1990**, *77* (2), 123–141. https://doi.org/10.1007/BF01114537.

(22) Reta, D.; Chilton, N. F. Uncertainty Estimates for Magnetic Relaxation Times and Magnetic Relaxation Parameters. *Phys. Chem. Chem. Phys.* **2019**, *21* (42), 23567–23575. https://doi.org/10.1039/C9CP04301B.

(23) Blackmore, W. J. A.; Gransbury, G. K.; Evans, P.; Kragskow, J. G. C.; Mills, D. P.; Chilton, N. F. Characterisation of Magnetic Relaxation on Extremely Long Timescales. *Phys. Chem. Chem. Phys.* **2023**, *25* (25), 16735–16744. https://doi.org/10.1039/D3CP01278F.

(24) Demir, S.; Zadrozny, J. M.; Long, J. R. Large Spin-Relaxation Barriers for the Low-Symmetry Organolanthanide Complexes [$Cp^*{}_2Ln(BPh_4)$] (Cp* = Pentamethylcyclopentadienyl; Ln = Tb, Dy). *Chem. Eur. J.* **2014**, *20* (31), 9524–9529. https://doi.org/10.1002/chem.201403751.

(25) Pugh, T.; Chilton, N. F.; Layfield, R. A. A Low-Symmetry Dysprosium Metallocene Single-Molecule Magnet with a High Anisotropy Barrier. *Angew. Chem. Int. Ed.* **2016**, *55* (37), 11082–11085. https://doi.org/10.1002/anie.201604346.

(26) Meng, Y. S.; Zhang, Y. Q.; Wang, Z. M.; Wang, B. W.; Gao, S. Weak Ligand-Field Effect from Ancillary Ligands on Enhancing Single-Ion Magnet Performance. *Chem. Eur. J.* **2016**, *22* (36), 12724–12731. https://doi.org/10.1002/chem.201601934.

(27) Guo, F.-S.; Layfield, R. A. Strong Direct Exchange Coupling and Single-Molecule Magnetism in Indigo-Bridged Lanthanide Dimers. *Chem. Commun.* **2017**, *53* (21), 3130–3133. https://doi.org/10.1039/C7CC01046J.

(28) Demir, S.; Boshart, M. D.; Corbey, J. F.; Woen, D. H.; Gonzalez, M. I.; Ziller, J. W.; Meihaus, K. R.; Long, J. R.; Evans, W. J. Slow Magnetic Relaxation in a Dysprosium Ammonia Metallocene Complex. *Inorg. Chem.* **2017**, *56* (24), 15049–15056. https://doi.org/10.1021/acs.inorgchem.7b02390.

(29) Chen, S. M.; Xiong, J.; Zhang, Y. Q.; Ma, F.; Sun, H. L.; Wang, B. W.; Gao, S. Dysprosium Complexes Bearing Unsupported Dy^{III}-Ge^{II}/Sn^{II} Metal-Metal Bonds as Single-Ion Magnets. *Chem. Commun.* **2019**, *55* (57), 8250–8253. https://doi.org/10.1039/c9cc00388f.

(30) Evans, P.; Reta, D.; Goodwin, C. A. P.; Ortu, F.; Chilton, N. F.; Mills, D. P. A Double-Dysprosocenium Single-Molecule Magnet Bound Together with Neutral Ligands. *Chem. Commun.* **2020**, *56*, 5677–5680. https://doi.org/10.1039/C9CC08945D.

(31) Errulat, D.; Gabidullin, B.; Mansikkamäki, A.; Murugesu, M. Two Heads Are Better than One: Improving Magnetic Relaxation in the Dysprosium Metallocene $DyCp^*_2BPh_4$ Upon Dimerization by Use of an Exceptionally Weakly-Coordinating Anion. *Chem. Commun.* **2020**, *56*, 5937–5940. https://doi.org/10.1039/d0cc01980a.

(32) Collins, R.; Heras Ojea, M. J.; Mansikkamäki, A.; Tang, J.; Layfield, R. A. Carbonyl Back-Bonding Influencing the Rate of Quantum Tunnelling in a Dysprosium Metallocene Single-Molecule Magnet. *Inorg. Chem.* **2020**, *59* (1), 642–647. https://doi.org/10.1021/acs.inorgchem.9b02895.

(33) Corner, S. C.; Gransbury, G. K.; Vitorica-Yrezabal, I. J.; Whitehead, G. F. S.; Chilton, N. F.; Mills, D. P. Monohalobenzene adducts of a dysprosocenium single-molecule magnet. *ChemRxiv*, **2023**, DOI: 10.26434/chemrxiv-2023-9l4wm.

(34) Fdez. Galván, I.; Vacher, M.; Alavi, A.; Angeli, C.; Aquilante, F.; Autschbach, J.; Bao, J. J.; Bokarev, S. I.; Bogdanov, N. A.; Carlson, R. K.; Chibotaru, L. F.; Creutzberg, J.; Dattani, N.; Delcey, M. G.; Dong, S. S.; Dreuw, A.; Freitag, L.; Frutos, L. M.; Gagliardi, L.; Gendron, F.; Giussani, A.; González, L.; Grell, G.; Guo, M.; Hoyer, C. E.; Johansson, M.; Keller, S.; Knecht, S.; Kovačević, G.; Källman, E.; Li Manni, G.; Lundberg, M.; Ma, Y.; Mai, S.; Malhado, J. P.; Malmqvist, P. Å.; Marquetand, P.; Mewes, S. A.; Norell, J.; Olivucci, M.; Oppel, M.; Phung, Q. M.; Pierloot, K.; Plasser, F.; Reiher, M.; Sand, A. M.; Schapiro, I.; Sharma, P.; Stein, C. J.; Sørensen, L. K.; Truhlar, D. G.; Ugandi, M.; Ungur, L.; Valentini, A.; Vancoillie, S.; Veryazov, V.; Weser, O.; Wesołowski, T. A.; Widmark, P. O.; Wouters, S.; Zech, A.; Zobel, J. P.; Lindh, R. OpenMolcas: From Source Code to Insight. *J. Chem. Theory Comput.* **2019**, *15* (11), 5925–5964. https://doi.org/10.1021/acs.jctc.9b00532.

(35) Roos, B. O.; Veryazov, V.; Widmark, P.-O. Relativistic Atomic Natural Orbital Type Basis Sets for the Alkaline and Alkaline-Earth Atoms Applied to the Ground-State Potentials for the Corresponding Dimers. *Theor. Chem. Acc.* **2004**, *111* (2–6), 345–351. https://doi.org/10.1007/s00214-003-0537-0.

(36) Roos, B. O.; Lindh, R.; Malmqvist, P. Å.; Veryazov, V.; Widmark, P. O. Main Group Atoms and Dimers Studied with a New Relativistic ANO Basis Set. *J. Phys. Chem. A* **2004**, *108* (15), 2851–2858. https://doi.org/10.1021/jp031064+.

(37) Roos, B. O.; Lindh, R.; Malmqvist, P.-Å.; Veryazov, V.; Widmark, P.-O. New Relativistic ANO Basis Sets for Transition Metal Atoms. *J. Phys. Chem. A* **2005**, *109* (29), 6575–6579. https://doi.org/10.1021/jp0581126.

(38) Roos, B. O.; Lindh, R.; Malmqvist, P.-Å.; Veryazov, V.; Widmark, P.-O.; Borin, A. C. New Relativistic Atomic Natural Orbital Basis Sets for Lanthanide Atoms with Applications to the Ce Diatom and LuF_3. *J. Phys. Chem. A* **2008**, *112* (45), 11431–

11435. https://doi.org/10.1021/jp803213j.

(39) Chibotaru, L. F.; Ungur, L. Ab Initio Calculation of Anisotropic Magnetic Properties of Complexes. I. Unique Definition of Pseudospin Hamiltonians and Their Derivation. *J. Chem. Phys.* **2012**, *137* (6), 064112. https://doi.org/10.1063/1.4739763.
